# Conditional Gaussian Systems for Multiscale Nonlinear Stochastic Systems: Prediction, State Estimation and Uncertainty Quantification

**DOI:** 10.3390/e20070509

**Published:** 2018-07-04

**Authors:** Nan Chen, Andrew J. Majda

**Affiliations:** 1 Department of Mathematics and Center for Atmosphere Ocean Science, Courant Institute of Mathematical Sciences, New York University, New York, NY 10012, USA; 2 Center for Prototype Climate Modeling, New York University Abu Dhabi, Saadiyat Island, Abu Dhabi 129188, UAE

**Keywords:** conditional Gaussian systems, multiscale nonlinear stochastic systems, physics-constrained nonlinear stochastic models, stochastically coupled reaction–diffusion models, conditional Gaussian mixture, superparameterization, conformation theory, model error, hybrid strategy, parameter estimation

## Abstract

A conditional Gaussian framework for understanding and predicting complex multiscale nonlinear stochastic systems is developed. Despite the conditional Gaussianity, such systems are nevertheless highly nonlinear and are able to capture the non-Gaussian features of nature. The special structure of the system allows closed analytical formulae for solving the conditional statistics and is thus computationally efficient. A rich gallery of examples of conditional Gaussian systems are illustrated here, which includes data-driven physics-constrained nonlinear stochastic models, stochastically coupled reaction–diffusion models in neuroscience and ecology, and large-scale dynamical models in turbulence, fluids and geophysical flows. Making use of the conditional Gaussian structure, efficient statistically accurate algorithms involving a novel hybrid strategy for different subspaces, a judicious block decomposition and statistical symmetry are developed for solving the Fokker–Planck equation in large dimensions. The conditional Gaussian framework is also applied to develop extremely cheap multiscale data assimilation schemes, such as the stochastic superparameterization, which use particle filters to capture the non-Gaussian statistics on the large-scale part whose dimension is small whereas the statistics of the small-scale part are conditional Gaussian given the large-scale part. Other topics of the conditional Gaussian systems studied here include designing new parameter estimation schemes and understanding model errors.

## Contents



**1 Introduction**


**3**


**2 Overview: Data vs. Model, Data-Driven Modeling Framework, and Efficient Data Assimilation and Prediction Strategies with Solvable Conditional Statistics**



**6**


**3 A Summary of the General Mathematical Structure of Nonlinear Conditional Gaussian Systems**


**8**


3.1 Conditional Gaussian Nonlinear Dynamical Systems . . . . . . . . . . . . . . . . . . . .

8

3.2 Kalman–Bucy Filter: The Simplest and Special Data Assimilation Example within Conditional Gaussian Framework . . . . . . . . . . . . . . . . . . . . . . . . . . . . . . .

9

3.3 Physics-Constrained Nonlinear Models with Conditional Gaussian Statistics . . . . . . .

9

3.4 Multiscale Conditional Gaussian with MTV Stochastic Modeling Strategy . . . . . . . .

12

**4 A Gallery of Examples of Conditional Gaussian Systems**


**13**


4.1 Physics-Constrained Nonlinear Low-Order Stochastic Models . . . . . . . . . . . . . . .

13


4.1.1 The Noisy Versions of Lorenz Models . . . . . . . . . . . . . . . . . . . . . . . . .


13



4.1.2 Nonlinear Stochastic Models for Predicting Intermittent MJO and Monsoon Indices



17


4.1.3 A Simple Stochastic Model with Key Features of Atmospheric Low-Frequency Variability . . . . . . . . . . . . . . . . . . . . . . . . . . . . . . . . . . . . . . . . .


18


4.1.4 A Nonlinear Triad Model with Multiscale Features . . . . . . . . . . . . . . . . .


19


4.1.5 Conceptual Models for Turbulent Dynamical Systems. . . . . . . . . . . . . . . .


21


4.1.6 A Conceptual Model of the Coupled Atmosphere and Ocean . . . . . . . . . . .


23


4.1.7 A Low-Order Model of Charney–DeVore Flows . . . . . . . . . . . . . . . . . . .


25


4.1.8 A Paradigm Model for Topographic Mean Flow Interaction . . . . . . . . . . . .


25

4.2 Stochastically Coupled Reaction–Diffusion Models in Neuroscience and Ecology . . . .

27


4.2.1 Stochastically Coupled FitzHugh–Nagumo (FHN) Models . . . . . . . . . . . . .


27


4.2.2 The Predator–Prey Models . . . . . . . . . . . . . . . . . . . . . . . . . . . . . . .


30


4.2.3 A Stochastically Coupled SIR Epidemic Model . . . . . . . . . . . . . . . . . . .


32


4.2.4 A Nutrient-Limited Model for Avascular Cancer Growth . . . . . . . . . . . . . .


33

4.3 Large-Scale Dynamical Models in Turbulence, Fluids and Geophysical Flows . . . . . .

34


4.3.1 The MJO Stochastic Skeleton Model . . . . . . . . . . . . . . . . . . . . . . . . . .


34


4.3.2 A Coupled El Niño Model Capturing Observed El Niño Diversity . . . . . . . .


37


4.3.3 The Boussinesq Equation . . . . . . . . . . . . . . . . . . . . . . . . . . . . . . . .


38


4.3.4 Darcy–Brinkman–Oberbeck–Boussinesq System—Convection Phenomena in Porous Media. . . . . . . . . . . . . . . . . . . . . . . . . . . . . . . . . . . . . . .


40


4.3.5 The Rotating Shallow Water Equations . . . . . . . . . . . . . . . . . . . . . . . .


41

4.4 Coupled Observation-Filtering Systems for Filtering Turbulent Ocean Flows Using Lagrangian Tracers . . . . . . . . . . . . . . . . . . . . . . . . . . . . . . . . . . . . . . . .

42

4.5 Other Low-Order Models for Filtering and Prediction . . . . . . . . . . . . . . . . . . . .

43


4.5.1 Stochastic Parameterized Extended Kalman Filter Model . . . . . . . . . . . . . .


43


4.5.2 An Idealized Surface Wind Model . . . . . . . . . . . . . . . . . . . . . . . . . . .


44

**5 Algorithms Which Beat the Curse of Dimension for Fokker–Planck Equation for Conditional Gaussian Systems: Application to Statistical Prediction**



**45**


5.1 The Basic Algorithm with a Hybrid Strategy . . . . . . . . . . . . . . . . . . . . . . . . .

45

5.2 Beating the Curse of Dimension with Block Decomposition . . . . . . . . . . . . . . . . .

46

5.3 Statistical Symmetry . . . . . . . . . . . . . . . . . . . . . . . . . . . . . . . . . . . . . . .

48

5.4 Quantifying the Model Error Using Information Theory . . . . . . . . . . . . . . . . . . .

49

5.5 Applications to Statistical Prediction . . . . . . . . . . . . . . . . . . . . . . . . . . . . . .

49


5.5.1 Application to the Stochastically Coupled FHN Model with Multiplicative Noise Using Statistical Symmetry . . . . . . . . . . . . . . . . . . . . . . . . . . . . . . .


50



5.5.2 Application to the Two-Layer L-96 Model with Inhomogeneous Spatial Structures



51

**6 Multiscale Data Assimilation, Particle Filters, Conditional Gaussian Systems and Information Theory for Model Errors**



**55**


6.1 Parameter Estimation . . . . . . . . . . . . . . . . . . . . . . . . . . . . . . . . . . . . . . .

55


6.1.1 Direct Parameter Estimation Algorithm . . . . . . . . . . . . . . . . . . . . . . . .


56


6.1.2 Parameter Estimation Using Stochastic Parameterized Equations . . . . . . . . .


57


6.1.3 Estimating Parameters in the Unresolved Processes . . . . . . . . . . . . . . . . .


59

6.2 Data Assimilation with Physics-Constrained Forecast Models and Information Theory for Quantifying Model Errors . . . . . . . . . . . . . . . . . . . . . . . . . . . . . . . . . .

62


6.2.1 An Information Theoretical Framework for Data Assimilation and Prediction . .


62


6.2.2 Important Roles of Physics-Constrained Forecast Models in Data Assimilation .


63

6.3 Multiscale Data Assimilation with Particles Interacting with Conditional Gaussian Statistics . . . . . . . . . . . . . . . . . . . . . . . . . . . . . . . . . . . . . . . . . . . . . .

66


6.3.1 A General Description . . . . . . . . . . . . . . . . . . . . . . . . . . . . . . . . . .


66


6.3.2 Particle Filters with Superparameterization . . . . . . . . . . . . . . . . . . . . . .


67


6.3.3 Clustered Particle Filters and Mutiscale Data Assimilation . . . . . . . . . . . . .


68


6.3.4 Blended Particle Methods with Adaptive Subspaces for Filtering Turbulent Dynamical Systems . . . . . . . . . . . . . . . . . . . . . . . . . . . . . . . . . . . .


70


6.3.5 Extremely Efficient Multi-Scale Filtering Algorithms: SPEKF and Dynamic Stochastic Superresolution (DSS) . . . . . . . . . . . . . . . . . . . . . . . . . . . .


70

**7 Conclusions**


**71**


**References**


**72**


## 1. Introduction

Multiscale nonlinear dynamical systems are ubiquitous in different areas, including geoscience, engineering, neural science and material science [1,2,3,4,5,6,7,8]. They are characterized by a large dimensional phase space and a large dimension of strong instabilities which transfer energy throughout the system. Many non-Gaussian characteristics, such as extreme and rare events, intermittency and fat-tailed probability density functions (PDFs), are often observed in these multiscale nonlinear dynamical systems [1,9,10]. Key mathematical issues are their basic mathematical structural properties and qualitative features [2,3,11,12], short- and long-range forecasting [13,14,15], uncertainty quantification (UQ) [16,17,18], and state estimation, filtering or data assimilation [15,19,20,21,22]. Due to the lack of physical understanding or the inadequate resolution in the models because of the current computing power, coping with the inevitable model errors that arise in approximating such complex systems becomes a necessary and crucial issue in dealing with these multiscale nonlinear dynamical systems [16,23,24,25,26]. Understanding and predicting complex multiscale turbulent dynamical systems involve the blending of rigorous mathematical theory, qualitative and quantitative modelling, and novel numerical procedures [2,27].

Despite the fully nonlinearity in many multiscale turbulent dynamical systems and the non-Gaussian features in both the marginal and joint PDFs, these systems have conditional Gaussian structures [1,15,28,29]. Writing the state variables as $u=(u_{I},u_{\mathrm{II}})$, the conditional Gaussianity means that once the trajectories of $u_{I}$ are given, the dynamics of $u_{\mathrm{II}}$ conditioned on these highly nonlinear observed trajectories become Gaussian processes. In this article, we develop a general conditional Gaussian framework for mathematical modeling, prediction, state estimation and UQ. One of the desirable features of such conditional Gaussian system is that it allows closed analytical formulae for updating the conditional statistics of $u_{\mathrm{II}}$ given the trajectories of $u_{I}$ [28,30]. This facilitates the development of both rigorous mathematical theories and efficient numerical algorithms for these complex multiscale turbulent dynamical systems.

With the great progress in developing and improving the observational networks, a vast amount of observational data is now available. An important scientific issue is to make use of these observational data to build data-driven models that advance the understanding of the underlying physical processes and improve the prediction skill. Physics-constrained nonlinear stochastic models are the recent development of data-driven statistical-dynamical models for the time series of a partial subset of observed variables, which arise from observations of nature or from an extremely complex physical model [31,32]. The physics-constrained nonlinear stochastic modeling framework builds on early works for single layer models without memory effects, which uses physical analytic properties to constrain data-driven methods. They succeed in overcoming both the finite-time blowup and the lack of physical meaning issues in various ad hoc multi-layer regression models [31,33]. Many of the physics-constrained nonlinear stochastic models belong to the conditional Gaussian framework, including the noisy version of the famous Lorenz 63 and 84 models as well as a two-layer Lorenz 96 model [34,35,36]. In addition, two important conceptual models for turbulent dynamical systems [37,38] and a conceptual model of the coupled atmosphere and ocean [39] also fit perfectly into the physics-constrained nonlinear stochastic modeling framework with conditional Gaussian structures. These models are extremely useful for testing various new multiscale data assimilation and prediction schemes [1,38,40]. Other physics-constrained nonlinear stochastic models with conditional Gaussian structures include a low-order model of Charney–DeVore flows [29] and a paradigm model for topographic mean flow interaction [32]. Notably, the physics-constrained nonlinear stochastic models combining with the conditional Gaussian framework have been successfully applied for the real-time prediction and data assimilation of the Madden–Julian oscillation (MJO) and monsoon [41,42,43,44], which are the dominant modes of intraseasonal variabilities in nature.

In many multiscale turbulent dynamical systems, there is a natural time scale separation between different variables. Therefore, the MTV strategy [45,46,47,48] (named after Majda, Timofeyev and Vanden-Eijnden) can be applied to these systems for stochastic mode reduction. Using the MTV strategy, the equations of motion for the unresolved fast modes are modified by representing the nonlinear self-interactions terms between unresolved modes by damping and stochastic terms. The resulting systems then naturally belong to the conditional Gaussian framework and they also preserve physics-constrained properties. Examples include a simple stochastic model with key features of atmospheric low-frequency variability [49] and a simple prototype nonlinear stochastic model with triad interactions that mimics structural features of low-frequency variability of global circulation models (GCM) [50] .

In addition to the data-driven physics-constrained nonlinear stochastic models, many large-scale dynamical models in turbulence, fluids and geophysical flows also have conditional Gaussian structures. These stochastic partial differential equation (SPDE) models include the stochastic skeleton model for the MJO [51] and a coupled El Niño dynamical model capturing the El Niño diversity [52], where nonlinearity interacts with state-dependent (multiplicative) noise and many salient non-Gaussian features such as intermittency and fat-tailed PDFs are observed in these systems. The stochastically forced Boussinesq equation [11] for the Rayleigh–Bénard convection [53,54] and strongly stratified flows in geophysics [55] as well as its generalized version, namely the Darcy–Brinkman–Oberbeck–Boussinesq system [56] for the convection phenomena in porous media, also belong to the conditional Gaussian framework. Likewise, the multiscale shallow water equation [11] with coupled geostrophically balanced (GB) and gravity modes fits into the conditional Gaussian model family. On the other hand, quite a few stochastically coupled reaction–diffusion models in neuroscience and ecology illustrate conditional Gaussian structures as well. Examples include the stochastically coupled FitzHugh–Nagumo (FHN) models [57], the predator–prey models [58], a stochastically coupled Susceptible-Infectious-Recovered (SIR) epidemic model [59] and a nutrient-limited model for avascular cancer growth [60]. Furthermore, the conditional Gaussian framework can be applied to model the coupled observation-filtering systems for the state estimation of turbulent ocean flows using Lagrangian tracers [61,62,63]. The framework can also be utilized to develop cheap and effective stochastic parameterizations for turbulent dynamical systems [64,65].

One of the key issues in studying the complex multiscale nonlinear turbulent dynamical systems is to solve the time evolution of the associated PDFs, which is extremely useful in ensemble prediction, data assimilation as well as understanding the intermittency and extreme events. The time evolution of the PDFs associated with the underlying turbulent dynamical systems is described by the so-called Fokker–Planck equation [66,67]. Due to the complexity of many multiscale turbulent dynamical systems, the dimension of the systems can be quite large. However, solving the Fokker–Planck equation in high dimensions is a well-known notoriously difficult problem. Traditional numerical methods such as finite element and finite difference as well as the direct Monte Carlo simulations all suffer from the curse of dimension [68,69]. Nevertheless, for the conditional Gaussian systems, efficient statistically accurate algorithms can be developed for solving the Fokker–Planck equation in high dimensions and thus beat the curse of dimension. The algorithms involve a hybrid strategy that requires only a small number of ensembles [38]. Specifically, a conditional Gaussian mixture in a high-dimensional subspace via an extremely efficient parametric method is combined with a judicious non-parametric Gaussian kernel density estimation in the remaining low-dimensional subspace. The parametric method provides closed analytical formulae for determining the conditional Gaussian distributions in the high-dimensional subspace and is therefore computationally efficient and accurate. The full non-Gaussian PDF of the system is then given by a Gaussian mixture [38]. For even larger dimensional systems, a judicious block decomposition and statistical symmetry are further applied that facilitate an extremely efficient parallel computation and a significant reduction of sample numbers [36]. These algorithms are applied here to the statistical prediction of a stochastically coupled FHN model with 1500 dimensions and an inhomogeneous two-layer Lorenz 96 model with 240 dimensions. Significant prediction skill shows the advantages of these algorithms in terms of both accuracy and efficiency.

The conditional Gaussian framework also provides a power tool for the multiscale data assimilation. In fact, data assimilation of turbulent signals is an important challenging problem because of the extremely complicated large dimension of the signals and incomplete partial noisy observations that usually mix the large scale mean flow and small scale fluctuations. Due to the limited computing power, it is desirable to use multiscale forecast models which are cheap and fast to mitigate the curse of dimensionality in turbulent systems. An extremely cheap multiscale data assimilation scheme, called stochastic superparameterization [70,71,72,73], resolves the large-scale mean flow on a coarse grid and replaces the nonlinear small-scale eddy terms by quasilinear stochastic processes on formally infinite embedded domains where the stochastic processes are Gaussian conditional to the large scale mean flow. The key ingredient of such a multiscale data assimilation method is the systematic use of conditional Gaussian mixtures which make the methods efficient by filtering a subspace whose dimension is smaller than the full state. This conditional Gaussian closure approximation results in a seamless algorithm without using the high resolution space grid for the small scales and is much cheaper than the conventional superparameterization, with significant success in difficult test problems [71,72,74] including the Majda-McLaughlin-Tabak (MMT) model [71,75] and ocean turbulence [76,77,78]. The method uses particle filters [20,79] or ensemble filters on the large scale part [75,76] whose dimension is small enough so that the non-Gaussian statistics of the large scale part can be calculated from a particle filter, whereas the statistics of the small scale part are conditionally Gaussian given the large scale part. This framework is not restricted to superparameterization as the forecast model and other cheap forecast models can also be employed. In fact, another multiscale filtering algorithm with quasilinear Gaussian dynamically orthogonality method as the forecast method in an adaptively evolving low dimensional subspace has been developed [80]. The multiscale filtering also provides a mathematical framework for representation errors, which are due to the contribution of unresolved scales [81,82] in the observations. Other theoretic and applied studies of the conditional Gaussian framework include effective parameter estimation, model reduction and the understanding of various model errors using information theory.

The remaining of the article is organized as follows. Section 2 provides an overview of data, model and data-driven modeling framework as well as efficient data assimilation and prediction strategies with solvable conditional statistics. Section 3 summarizes the general mathematical structure of nonlinear conditional Gaussian systems, the physics-constrained nonlinear stochastic models and the application of the MTV strategy to the conditional Gaussian systems. Then, a gallery of examples of conditional Gaussian systems is shown in Section 4. Section 5 involves the effective statistically accurate algorithms that beat the curse of dimension for Fokker–Planck equation for conditional Gaussian systems together with their applications to statistical prediction. The topics related to the parameter estimation, model error and multiscale data assimilation utilizing the conditional Gaussian framework are shown in Section 6. The article is concluded in Section 7.

## 2. Overview: Data vs. Model, Data-Driven Modeling Framework, and Efficient Data Assimilation and Prediction Strategies with Solvable Conditional Statistics

A central contemporary issue for complex turbulent systems is to use data to improve scientific understanding of the underlying physics, make real-time predictions, and quantify the uncertainty in long range forecasting [1,27]. Recently, with the great progress in developing and improving the observational networks, vast amount of observational data are now available. Many purely data-driven statistical methods (regression, clustering and analog forecast etc.) [83,84,85,86,87] are thus developed and have become popular approaches in attempting to understand and predict nature. Despite the success in a crude understanding of nature in terms of explaining and forecasting some of the coarse-grained variables in the largest scale to some extent, these purely data-driven statistical methods usually cannot discover the nonlinear and intermittent nature in the underlying complex dynamics. They often fail to reveal the non-trivial interactions between physical processes in different scales either. In addition, these purely data-driven statistical methods typically require a large training dataset in order to obtain complete and unbiased information from nature, which is however infeasible in many areas, including climate, atmosphere and ocean science. In fact, satellite observations are only available for a few decades [88,89] which are far from enough in understanding decadal or interannual variabilities containing rare and extreme events. Note that many complex turbulent systems in nature are in high dimensions, and therefore most purely data driven statistical methods are extremely expensive to use.

Therefore, combining model with data becomes necessary in understanding and predicting nature. Suitable models involve important physical mechanisms and they can be used for real-time prediction by incorporating only a small amount of data. In many simple but natural scenarios, some low-dimensional physical variables are observed and low-order nonlinear stochastic models are preferred for describing their behavior. Using data outcome to fit a quadratic regression model [90,91] is a data-driven modeling strategy which outweighs the linear regression models and allows nonlinearity and memory in time. However, there is no physical information in such quadratic regression models. In fact, it has been shown in [33] via rigorous mathematical theory that such ad hoc nonlinear regression strategies can exhibit finite time blow-up and pathological invariant measures even though they fit the data with high precision. To include physical information into the models, systematic physics-constrained multi-level quadratic regression models have been developed [31,32]. These models avoid pathological behavior by incorporating physical constraints with nonlinear energy-conserving principles developed in the earlier stochastic climate modeling strategy [45]. Meanwhile, these physics-constrained models allow memory in effects. Although the number of the parameters in physics-constrained nonlinear models can be large, the models are in general robust with respect to the perturbation of the parameters around their optimal values [41]. This is crucial in practice because it requires only a crude estimation of the parameters for the model, which greatly reduces the computational cost for searching in high-dimensional parameter space. A striking real-world application of these physics-constrained nonlinear models is to assess the predictability limits of time series associated with the tropical intraseasonal variability such as the the Madden–Julian oscillation (MJO) and monsoon. It has been shown in [41,42,43] that these physics-constrained nonlinear models are able to reach the predictability limits of the large-scale MJO and monsoon and improves prediction skill using the low-order models. Notably, the physics-constrained nonlinear stochastic models require only a short training period [42,44] because the model development automatically involves a large portion of the information of nature. This is extremely important since practically only a limited observational data is available. In fact, comparable and even slightly more skillful prediction results have been found using the physics-constrained nonlinear model compared with those using non-parametric methods in predicting the MJO and monsoon intermittent time series [92,93], but the prediction using the physics-constrained nonlinear model adopted a much shorter training period [44].

Another important reason that entails the model in understanding and predicting nature is due to the fundamental limitations in measuring the observations. In fact, only partial observations are available in most applications and observational noise is inevitable. For example, the sea surface temperature is easy to measure, but the temperature in the deep ocean is hard to estimate. In addition, although the surface wind in the entire earth can be measured by satellites, the measured signals usually contain large noise. Therefore, models are combined with the available observations for the state estimation of the unobserved variables as well as reducing the noise in the observed ones. This is known as data assimilation or filtering [15,20,22]. Note that most of the complex multiscale dynamical systems involve strong nonlinearity and extreme events. Thus, the classical Kalman filter that works only for linear models [94] fails to capture the nonlinear and non-Gaussian features in nature. Although the so-called particle filter [95,96] is able to recover all the non-Gaussian statistics, it is computationally expensive and can only be applied to low-dimensional scenarios, which are typically not the case in most applications. In fact, even a global weather model with very coarse grid points at every 200 km already has about $10^{6}$ state variables. Other direct strategies in handling large dimensional systems, such as the ensemble Kalman filters [97,98], can also be problematic. For example, they are not immune from “catastrophic filter divergence” (diverge beyond machine infinity) when observations are sparse, even when the true signal is a dissipative system [99,100]. Therefore, designing new efficient strategies for data assimilation and prediction that are accurate and can overcome the curse of dimension is crucial in studying complex multiscale nonlinear dynamical systems with only noisy partial observations [20,27,65,101,102]. Since both the data assimilation and prediction involve running forward the dynamical models (known as the forecast models), the development of new strategies entails the understanding and utilization of model structures.

Due to the complexity in many complex multiscale turbulent dynamical systems, developing cheap and effective approximate models which nevertheless capture the main characteristics of the underlying dynamics becomes necessary in data assimilation, prediction and the understanding of nature. In particular, novel stochastic parameterization schemes play a significant role in reducing the model complexity while retaining the key features of various multiscale turbulent dynamical systems. These key features include the statistical feedback from small to large scales, accurate dynamical and statistical behavior in large scale variables, and the main effect of the large-scale variables on the statistical evolution of small scale processes. Then, efficient hybrid strategy can be developed for dealing with large and small scale variables, respectively.

A simple yet practically useful strategy in filtering nonlinear and intermittent signals is via the so-called stochastic parameterized extended Kalman filter (SPEKF) type of the forecast model [64,65]. The idea of the SKEPF model is that the small or unresolved scale variables are stochastically parameterized by cheap linear and Gaussian processes, representing stochastic damping, stochastic phase and stochastic forcing. Despite the model error in using such Gaussian approximations for the unresolved nonlinear dynamics, these Gaussian processes succeed in providing accurate statistical feedback from the unresolved scales to the resolved ones and thus the intermittency and non-Gaussian features as observed in the resolved variables can be accurately recovered. The statistics in the SPEKF model can also be solved with exact and analytic formulae, which allow an accurate and efficient estimation of the model states. The SPEKF type of model has been used for filtering mutiscale turbulent dynamical systems [20], stochastic superresolution [103] and filtering Navier–Stokes equations with model error [104]. A detailed description of the SPEKF model can be found in Section 4.5.1. Notably, the SPEKF model involves a hybrid approach, where certain cheap and statistically solvable Gaussian approximations are used to describe the statistical features of the unresolved (or small scale) variables in a large dimensional subspace while the highly nonlinear dynamical features of the resolved (or large scale) variables as well as their interactions with the unresolved variables are retained in a relatively low dimensional subspace. Such a strategy can be used as a guideline for designing suitable approximate forecast models for various complex multiscale turbulent dynamical systems.

For multiscale data assimilation of many realistic and complex turbulent dynamical systems, an extremely cheap scheme, called stochastic superparameterization [70,71,72,73], has been developed, which makes use of a similar hybrid strategy as discussed above but with a more refined setup. In the stochastic superparameterization, the large-scale mean flow is resolved on a coarse grid and the nonlinear small-scale eddy terms are replaced by quasilinear stochastic processes on formally infinite embedded domains where these stochastic processes are Gaussian conditional to the large scale mean flow. The key ingredient of such a multiscale data assimilation method is the systematic use of conditional Gaussian mixtures which make the methods efficient by filtering a subspace whose dimension is smaller than the full state. This conditional Gaussian closure approximation results in a seamless algorithm without using the high resolution space grid for the small scales and is thus extremely efficient. The method uses particle filters [20,79] or ensemble filters on the large scale part [75,76] whose dimension is small enough so that the non-Gaussian statistics of the large scale part can be calculated from a particle filter, whereas the statistics of the small scale part are conditionally Gaussian given the large scale part. See Section 6.3 for more details. Note that such ideas can also be applied to other cheap forecast models in addition to the superparameterization. For example, another multiscale filtering algorithm with quasilinear Gaussian dynamically orthogonality method as the forecast method in an adaptively evolving low dimensional subspace has been developed in [80].

Due to the significance in model approximation, data assimilation and prediction, it is necessary to develop a general framework for the complex multiscale nonlinear turbulent dynamical systems with conditional Gaussian structures. The key idea of such conditional Gaussian systems is that a hybrid strategy can be applied to deal with the state variables $u=(u_{I},u_{\mathrm{II}})$, where particle methods are used to solve the non-Gaussian statistics in the relatively low dimensional subspace associated with $u_{I}$ and extremely cheap algorithms with closed analytical formulae are adopted to solve the conditional Gaussian statistics of $u_{\mathrm{II}}$ conditioned on $u_{I}$ in the remaining high dimensional subspace. The Gaussianity in the conditional statistics are usually quite accurate as approximations since the small or unresolved scales with the given (or fixed) large-scale variables usually represent fluctuations in multiscale systems and the statistics are close to Gaussian. Nevertheless, the marginal statistics of the small-scale variables $u_{\mathrm{II}}$ themselves can be highly non-Gaussian, which is one of the salient features as in nature [37]. Notably, developing such computationally efficient models that explores conditional statistics also involves the mutual feedback between large scale and small scale variables. Thus, the full system is completely nonlinear and allows physics-constrained nonlinear interactions. The general conditional Gaussian nonlinear modeling framework provides a powerful tool in multiscale data assimilation, statistical prediction, solving high-dimensional PDFs as well as parameter estimation, causality analysis and understanding model errors.

## 3. A Summary of the General Mathematical Structure of Nonlinear Conditional Gaussian Systems

### 3.1. Conditional Gaussian Nonlinear Dynamical Systems

The conditional Gaussian systems have the following abstract form [28]:

(1) $$\begin{array}{cc} du_{I} & =\left[A_{0}\left(t,u_{I}\right)+A_{1}\left(t,u_{I}\right)u_{\mathrm{II}}\right]dt+\Sigma_{I}\left(t,u_{I}\right)dW_{I}\left(t\right), \end{array}$$

(2) $$\begin{array}{cc} du_{\mathrm{II}} & =\left[a_{0}\left(t,u_{I}\right)+a_{1}\left(t,u_{I}\right)u_{\mathrm{II}}\right]dt+\Sigma_{\mathrm{II}}\left(t,u_{I}\right)dW_{\mathrm{II}}\left(t\right). \end{array}$$

Once $u_{I}\left(s\right)$ for s≤t is given, $u_{\mathrm{II}}\left(t\right)$ conditioned on $u_{I}\left(s\right)$ becomes a Gaussian process:

(3) $$p\left(u_{\mathrm{II}}\left(t\right)|u_{I}\left(s\leq t\right)\right)∼N\left({\bar{u}}_{\mathrm{II}}\left(t\right),R_{\mathrm{II}}\left(t\right)\right).$$

Despite the conditional Gaussianity, the coupled system (1) and (2) remains highly nonlinear and is able to capture the non-Gaussian features as in nature. The conditional Gaussian distribution $p\left(u_{\mathrm{II}}\left(t\right)|u_{I}\left(s\leq t\right)\right)$ in (3) has closed analytic form [30]

(4) $$\begin{array}{c} d{\bar{u}}_{\mathrm{II}}\left(t\right)=\left[a_{0}\left(t,u_{I}\right)+a_{1}\left(t,u_{I}\right){\bar{u}}_{\mathrm{II}}\right]dt+\left(R_{\mathrm{II}}A_{1}^{*}\left(t,u_{I}\right)\right){\left(\Sigma_{I}\Sigma_{I}^{*}\right)}^{-1}\left(t,u_{I}\right)\times \\ \left[du_{I}-\left(A_{0}\left(t,u_{I}\right)+A_{1}\left(t,u_{I}\right){\bar{u}}_{\mathrm{II}}\right)dt\right], \end{array}$$

(5) $$\begin{array}{c} dR_{\mathrm{II}}\left(t\right)=\{a_{1}\left(t,u_{I}\right)R_{\mathrm{II}}+R_{\mathrm{II}}a_{1}^{*}\left(t,u_{I}\right)+\left(\Sigma_{\mathrm{II}}\Sigma_{\mathrm{II}}^{*}\right)\left(t,u_{I}\right)\\ -\left(R_{\mathrm{II}}A_{1}^{*}\left(t,u_{I}\right)\right){\left(\Sigma_{I}\Sigma_{I}^{*}\right)}^{-1}\left(t,u_{I}\right){\left(R_{\mathrm{II}}A_{1}^{*}\left(t,u_{I}\right)\right)}^{*}\}dt, \end{array}$$

which can be solved in an exact and efficient way. The conditional Gaussian framework (1)-(2)-(5) is useful in the parameter estimation, data assimilation, prediction and uncertainty quantification of complex turbulent dynamical systems as will be discussed throughout this article.

In this article, many conditional Gaussian systems with complex turbulent dynamical structures will be studied. For the convenience of the reader, we will always use a blue color to represent the variables that belong to $u_{I}$ and use a magenta color to denote those for $u_{\mathrm{II}}$.

### 3.2. Kalman–Bucy Filter: The Simplest and Special Data Assimilation Example within Conditional Gaussian Framework

A special case of the general conditional Gaussian framework (1) and (2) is the so-called Kalman–Bucy filter [105,106,107,108]. The Kalman–Bucy filter is a continuous time version of the Kalman filter [21,94] and it deals with the linear coupled systems:

(6) $$\begin{array}{cc} du_{I} & =\left[A_{0}\left(t\right)+A_{1}\left(t\right)u_{\mathrm{II}}\right]dt+\Sigma_{I}\left(t\right)dW_{I}\left(t\right) \end{array}$$

(7) $$\begin{array}{cc} du_{\mathrm{II}} & =\left[a_{0}\left(t\right)+a_{1}\left(t\right)u_{\mathrm{II}}\right]dt+\Sigma_{\mathrm{II}}\left(t\right)dW_{\mathrm{II}}\left(t\right) \end{array}$$

In (6) and (7), all the vectors and matrices $A_{0},A_{1},a_{0},a_{1},\Sigma_{I}$ and $\Sigma_{\mathrm{II}}$ are functions of only *t* and they have no dependence on $u_{I}$ in order to guarantee the linearity in the coupled system. In the Kalman–Bucy filter, (7) is the underlying dynamics and (6) is the observational process. The observation cannot change the underlying dynamics and therefore no $u_{I}$ appears in (7).

The filter estimate (also known as the posterior distribution) is the conditional distribution of $u_{\mathrm{II}}\left(t\right)$ given the observation $u_{I}\left(s\leq t\right)$, i.e., $p(u_{\mathrm{II}}\left(t\right)|u_{I}\left(s\leq t\right))$. In light of (4) and (5), the mean and variance of $p(u_{\mathrm{II}}|u_{I})$ has the following explicit expressions:

$$\begin{array}{cc} d{\bar{u}}_{\mathrm{II}}\left(t\right)= & \left[a_{0}\left(t\right)+a_{1}\left(t\right){\bar{u}}_{\mathrm{II}}\right]dt+\left(R_{\mathrm{II}}A_{1}^{*}\left(t\right)\right){\left(\Sigma_{I}\Sigma_{I}^{*}\right)}^{-1}\left(t\right)\left[du_{I}-\left(A_{0}\left(t\right)+A_{1}\left(t\right){\bar{u}}_{\mathrm{II}}\right)dt\right],\\ dR_{\mathrm{II}}\left(t\right)= & \left\{a_{1}\left(t\right)R_{\mathrm{II}}+R_{\mathrm{II}}a_{1}^{*}\left(t\right)+\left(\Sigma_{\mathrm{II}}\Sigma_{\mathrm{II}}^{*}\right)\left(t\right)-\left(R_{\mathrm{II}}A_{1}^{*}\left(t\right)\right){\left(\Sigma_{I}\Sigma_{I}^{*}\right)}^{-1}\left(t\right){\left(R_{\mathrm{II}}A_{1}^{*}\left(t\right)\right)}^{*}\right\}dt. \end{array}$$

Corresponding to (4) and (5), Chapter 6 of Bensoussan’s book [109] includes rigorous mathematical derivations of the exact solutions of the Kalman–Bucy filter and some other more general conditional Gaussian filters. It is also pointed out in [109] that all these filters belong to the general conditional Gaussian filtering framework in (1)-(2)-(4)-(5) introduced in [110], which is an early version of the book authored by Liptser and Shiryaev [30].

### 3.3. Physics-Constrained Nonlinear Models with Conditional Gaussian Statistics

Physics-constrained nonlinear stochastic models are the recent development of data driven statistical-dynamical models for the time series of a partial subset of observed variables, which arise from observations of nature or from an extremely complex physical model [31,32]. The physics-constrained nonlinear stochastic modeling framework builds on early works for single layer models without memory effects, which uses physical analytic properties to constrain data driven methods. These physics-constrained nonlinear stochastic models succeed in overcoming both the finite-time blowup and the lack of physical meaning issues in various ad hoc multi-layer regression models [31,33].

The physics-constrained nonlinear stochastic models contain energy-conserving quadratic nonlinear interactions [1,31,32], namely

(8) $$\begin{array}{cc} du & =\left[(L+D)u+B(u,u)+F(t)\right]dt+\Sigma \left(t,u\right)dW\left(t\right),\\ & \mathrm{with}\;u\cdot B(u,u)=0, \end{array}$$

where $u=(u_{I},u_{\mathrm{II}})$ and the dimensions of $u_{I}$ and $u_{\mathrm{II}}$ are $N_{I}$ and $N_{II}$, respectively. In (8), L+D is a linear operator representing dissipation and dispersion. Here, L is skew symmetric representing dispersion and D is a negative definite symmetric operator representing dissipative process such as surface drag, radiative damping, viscosity, etc. $B\left(u,u\right)$ is a bilinear term and it satisfies energy conserving property with $u\cdot B\left(u,u\right)=0$.

Many of the physics-constrained nonlinear stochastic models belong to the conditional Gaussian framework. The goal here is to derive a general class of conditional Gaussian physics-constrained nonlinear stochastic models. To this end, we rewrite the Equation (8) in the following way:

(9) $$\begin{array}{cc} du_{I} & =\left(L_{I,1}u_{I}+L_{I,2}u_{\mathrm{II}}+B_{I}\left(u,u\right)+F_{I}\right)dt+\Sigma_{I}\left(u_{I}\right)dW_{I},\\ du_{\mathrm{II}} & =\left(L_{\mathrm{II},1}u_{I}+L_{\mathrm{II},2}u_{\mathrm{II}}+B_{\mathrm{II}}\left(u,u\right)+F_{\mathrm{II}}\right)dt+\Sigma_{\mathrm{II}}\left(u_{I}\right)dW_{\mathrm{II}}, \end{array}$$

where the explicit dependence of the coefficients on time *t* has been omitted for notation simplicity. In (9), $L_{I,1}u_{I}$, $L_{I,2}u_{\mathrm{II}}$, $L_{\mathrm{II},1}u_{I}$ and $L_{\mathrm{II},2}u_{\mathrm{II}}$ correspond to the the linear term L+D in (8) while $B_{I}\left(u,u\right)$ and $B_{\mathrm{II}}\left(u,u\right)$ represent the nonlinear terms in the processes associated with the variables in (1) and those in (2), respectively. Since the conditional Gaussian systems do not allow quadratic nonlinear interactions between $u_{\mathrm{II}}$ and itself, both $B_{I}\left(u,u\right)$ and $B_{\mathrm{II}}\left(u,u\right)$ can be written down as follows:

(10) $$\begin{array}{cc} B_{I}\left(u,u\right) & =B_{I,1}\left(u_{I},u_{I}\right)+B_{I,2}\left(u_{I},u_{\mathrm{II}}\right),\\ B_{\mathrm{II}}\left(u,u\right) & =B_{\mathrm{II},1}\left(u_{I},u_{I}\right)+B_{\mathrm{II},2}\left(u_{I},u_{\mathrm{II}}\right), \end{array}$$

where $B_{\cdot ,1}\left(u_{I},u_{I}\right)$ stands for the quadratic terms involving only $u_{I}$ and $B_{\cdot ,2}\left(u_{I},u_{\mathrm{II}}\right)$ represents the quadratic interactions between $u_{I}$ and $u_{\mathrm{II}}$. Given the nonlinear terms in (10), the energy-conserving quadratic nonlinearity in (8) implies

(11) $$u_{I}\cdot \left(B_{I,1}\left(u_{I},u_{I}\right)+B_{I,2}\left(u_{I},u_{\mathrm{II}}\right)\right)+u_{\mathrm{II}}\cdot \left(B_{\mathrm{II},1}\left(u_{I},u_{I}\right)+B_{\mathrm{II},2}\left(u_{I},u_{\mathrm{II}}\right)\right)=0.$$

Inserting (10) into (9) yields the conditional Gaussian systems with energy-conserving quadratic nonlinear interactions,

(12) $$\begin{array}{cc} du_{I} & =\left(B_{I,1}\left(u_{I},u_{I}\right)+B_{I,2}\left(u_{I},u_{\mathrm{II}}\right)+L_{I,1}u_{I}+L_{I,2}u_{\mathrm{II}}+F_{I}\right)dt+\Sigma_{I}\left(u_{I}\right)dW_{I}, \end{array}$$

(13) $$\begin{array}{cc} du_{\mathrm{II}} & =\left(B_{\mathrm{II},1}\left(u_{I},u_{I}\right)+B_{\mathrm{II},2}\left(u_{I},u_{\mathrm{II}}\right)+L_{\mathrm{II},1}u_{I}+L_{\mathrm{II},2}u_{\mathrm{II}}+F_{\mathrm{II}}\right)dt+\Sigma_{\mathrm{II}}\left(u_{I}\right)dW_{\mathrm{II}}, \end{array}$$

Now, we explore the detailed forms of the energy-conserving nonlinear terms in (12) and (13).

We start with $B_{\mathrm{II},2}\left(u_{I},u_{\mathrm{II}}\right)$, which can be written as

(14) $$B_{\mathrm{II},2}\left(u_{I},u_{\mathrm{II}}\right)=S_{\mathrm{II}}\left(u_{I}\right)u_{\mathrm{II}},\;\mathrm{with}\;S_{\mathrm{II}}\left(u_{I}\right)=\sum_{j=1}^{N_{I}}S_{\mathrm{II},j}u_{I,j},$$

where each $S_{\mathrm{II},j}$ is a skew-symmetric matrix with $S_{\mathrm{II},j}^{T}=-S_{\mathrm{II},j}$ and $u_{I,j}$ is the *j*-th entry of $u_{I}$. The energy-conserving property is easily seen by multiplying $u_{\mathrm{II}}$ to $B_{\mathrm{II},2}\left(u_{I},u_{\mathrm{II}}\right)$ in (14),

$$u_{\mathrm{II}}\cdot B_{\mathrm{II},2}\left(u_{I},u_{\mathrm{II}}\right)=u_{\mathrm{II}}\cdot S\left(u_{I}\right)u_{\mathrm{II}}=\sum_{j=1}^{N_{I}}u_{I,j}\cdot \left(u_{\mathrm{II}}\cdot S_{\mathrm{II},j}u_{\mathrm{II}}\right)=0.$$

Due to the skew-symmetric property of $S_{\mathrm{II},j}$. In fact, $B_{\mathrm{II},2}\left(u_{I},u_{\mathrm{II}}\right)$ usually represents the internal oscillation with non-constant oscillation frequency that depends on $u_{I}$.

Next, $B_{I,2}\left(u_{I},u_{\mathrm{II}}\right)$ contains three components,

(15) $$B_{I,2}\left(u_{I},u_{\mathrm{II}}\right)=B_{I,2}^{1}\left(u_{I},u_{\mathrm{II}}\right)+B_{I,2}^{2}\left(u_{I},u_{\mathrm{II}}\right)+B_{I,2}^{3}\left(u_{I},u_{\mathrm{II}}\right).$$

One of the components in (15), say $B_{I,2}^{1}\left(u_{I},u_{\mathrm{II}}\right)$, has its own energy conservation, i.e.,

$$u_{I}\cdot B_{I,2}^{1}\left(u_{I},u_{\mathrm{II}}\right)=0.$$

Here, $B_{I,2}^{1}\left(u_{I},u_{\mathrm{II}}\right)=S_{I}\left(u_{I}\right)u_{\mathrm{II}}$ and therefore

(16) $$u_{I}\cdot S_{I}\left(u_{I}\right)u_{\mathrm{II}}=0,$$

where each column of $S_{I}\left(u_{I}\right)$ is given by

(17) $$S_{I,j}\left(u_{I}\right)=S_{I,j}u_{I},$$

with $S_{I,j}$ being a skew-symmetric matrix. Thus, with (17) in hand, (16) becomes

$$\sum_{j=1}^{N_{\mathrm{II}}}\left(u_{I}\cdot S_{I,j}\cdot u_{I}\right)u_{\mathrm{II},j}=0,$$

where $u_{\mathrm{II},j}$ is the *j*-th entry of $u_{\mathrm{II}}$.

The other two components of $B_{I,2}\left(u_{I},u_{\mathrm{II}}\right)$ in (12) involve the interactions with $B_{\mathrm{II},1}\left(u_{I},u_{I}\right)=B_{\mathrm{II},1}^{2}\left(u_{I},u_{I}\right)+B_{\mathrm{II},1}^{3}\left(u_{I},u_{I}\right)$ in (13). On one hand, the energy-conserving property in the following two terms is obvious,

(18) $$\begin{array}{cc} B_{I,2}^{2}\left(u_{I},u_{\mathrm{II}}\right) & =\sum_{j=1}^{N_{I}}\Gamma_{j}u_{I,j}u_{\mathrm{II}}, \end{array}$$

(19) $$\begin{array}{cc} B_{\mathrm{II},1}^{2}\left(u_{I},u_{I}\right) & =-\sum_{j=1}^{N_{I}}\Gamma_{j}^{T}u_{I}^{2}, \end{array}$$

where each $\Gamma_{j}$ is a $N_{I}\times N_{\mathrm{II}}$ matrix, $u_{I,j}$ is the *j*-th entry of $u_{I}$ and $u_{I}^{2}$ is a vector of size $N_{I}\times 1$ with the *j*-th entry being $u_{I,j}^{2}$. On the other hand, the remaining two terms $B_{I,2}^{3}\left(u_{I},u_{\mathrm{II}}\right)$ and $B_{\mathrm{II},1}^{3}\left(u_{I},u_{I}\right)$ are similar to those in (18)–(20) but deal with the cross-interactions between different components of $u_{I}$ such as replacing $u_{I}^{2}$ by $u_{I,j_{1}}u_{I,j_{2}}$ in (20). To this end, we define the following

(20) $$G\left(u_{I}\right)=\sum_{j=1}^{N_{I}}G_{j}u_{I,j},$$

which satisfies

(21) $$u_{I}\cdot G\left(u_{I}\right)u_{\mathrm{II}}-u_{\mathrm{II}}\cdot G^{T}\left(u_{I}\right)u_{I}=0.$$

In fact, (18)–(21) are important for generating the intermittent instability, where $u_{\mathrm{II}}$ plays the role of both damping and anti-damping for the dynamics of $u_{I}$.

Finally, $B_{I,1}\left(u_{I},u_{I}\right)$ involves any iterations between $u_{I}$ and itself that satisfies

(22) $$u_{I}\cdot B_{I,1}\left(u_{I},u_{I}\right)=0.$$

Therefore, with (14)–(22) in hand, the conditional Gaussian system with energy-conserving quadratic nonlinear interactions (12) and (13) has the following form:

(23) $$\begin{array}{cc} du_{I} & =\left(B_{I,1}\left(u_{I},u_{I}\right)+\sum_{j=1}^{N_{I}}\Gamma_{j}u_{I,j}u_{\mathrm{II}}+S_{I}\left(u_{I}\right)u_{\mathrm{II}}+G\left(u_{I}\right)u_{\mathrm{II}}+L_{I,1}u_{I}+L_{I,2}u_{\mathrm{II}}+F_{I}\right)dt+\Sigma_{I}\left(u_{I}\right)dW_{I}, \end{array}$$

(24) $$\begin{array}{cc} du_{\mathrm{II}} & =\left(S_{\mathrm{II}}\left(u_{I}\right)u_{\mathrm{II}}-\sum_{j=1}^{N_{I}}\Gamma_{j}^{T}u_{I}^{2}+L_{\mathrm{II},1}u_{I}+L_{\mathrm{II},2}u_{\mathrm{II}}-G^{T}\left(u_{I}\right)u_{I}+F_{\mathrm{II}}\right)dt+\Sigma_{\mathrm{II}}\left(u_{I}\right)dW_{\mathrm{II}}. \end{array}$$

### 3.4. Multiscale Conditional Gaussian with MTV Stochastic Modeling Strategy

Let’s start with a general nonlinear deterministic model with quadratic nonlinearity,

(25) $$du=\left[(L+D)u+B(u,u)+F(t)\right]dt,$$

where the notations of vectors and matrices are the same as in Section 3.3. In (25), the state variables are again decomposed into $u=(u_{I},u_{\mathrm{II}})$. Here, $\left(u_{I},u_{\mathrm{II}}\right)$ has multiscale features, where $u_{I}$ denotes the resolved variables that evolve slowly in time (e.g., climate variables) while $u_{\mathrm{II}}$ are unresolved or unobserved fast variables (e.g., weather variables). The system (25) can be written down into more detailed forms:

(26) $$\begin{array}{cc} du_{I}= & [\left(L_{11}+D_{11}\right)u_{I}+\left(L_{12}+D_{12}\right)u_{\mathrm{II}}+B_{11}^{1}\left(u_{I},u_{I}\right)\\ & +B_{12}^{1}\left(u_{I},u_{\mathrm{II}}\right)+B_{22}^{1}\left(u_{\mathrm{II}},u_{\mathrm{II}}\right)+F_{1}\left(t\right)]dt, \end{array}$$

(27) $$\begin{array}{cc} du_{\mathrm{II}}= & [\left(L_{22}+D_{22}\right)u_{\mathrm{II}}+\left(L_{21}+D_{21}\right)u_{I}+B_{11}^{2}\left(u_{I},u_{I}\right)\\ & +B_{12}^{2}\left(u_{I},u_{\mathrm{II}}\right)+B_{22}^{2}\left(u_{\mathrm{II}},u_{\mathrm{II}}\right)+F_{2}\left(t\right)]dt. \end{array}$$

Different from (12) and (13), the nonlinear interaction between fast scale variables themselves $B_{22}^{1}\left(u_{\mathrm{II}},u_{\mathrm{II}}\right)$ and $B_{22}^{2}\left(u_{\mathrm{II}},u_{\mathrm{II}}\right)$ are also included in (26) and (27). To make the mutiscale system (26) and (27) fit into the conditional Gaussian framework, two modifications are needed. First, if we link (26) and (27) with the general conditional Gaussian framework (1) and (2), the quadratic terms involving the interactions between $u_{\mathrm{II}}$ and itself, namely $B_{22}^{1}\left(u_{\mathrm{II}},u_{\mathrm{II}}\right)$ and $B_{22}^{2}\left(u_{\mathrm{II}},u_{\mathrm{II}}\right)$, are not allowed there. Second, stochastic noise is required at least to the system of $u_{I}$.

To fill in these gaps, the most natural way is to apply idea from the MTV strategy to the multiscale system (26) and (27). The MTV strategy [45,46,47,48], named after Majda, Timofeyev and Vanden-Eijnden, is a systematic mathematical strategy for stochastic mode reduction. The MTV strategy is a two-step procedure:

1. The equations of motion for the unresolved fast modes are modified by representing the nonlinear self-interactions terms between unresolved modes by stochastic terms.
2. The equations of motion for the unresolved fast modes are eliminated using the standard projection technique for stochastic differential equations.

In fact, we only need to take the first step in the MTV strategy here. Using ϵ to represent the time scale separation between $u_{I}$ and $u_{\mathrm{II}}$, the terms with quadratic nonlinearity of $u_{\mathrm{II}}$ and itself are approximated by

(28) $$\begin{array}{cc} B_{22}^{1}\left(u_{\mathrm{II}},u_{\mathrm{II}}\right) & \approx -\frac{\Gamma_{1}}{\epsilon }u_{\mathrm{II}}+\frac{\Sigma_{I}}{\sqrt{\epsilon }}W_{I},\\ B_{22}^{2}\left(u_{\mathrm{II}},u_{\mathrm{II}}\right) & \approx -\frac{\Gamma_{2}}{\epsilon }u_{\mathrm{II}}+\frac{\Sigma_{\mathrm{II}}}{\sqrt{\epsilon }}W_{\mathrm{II}}. \end{array}$$

That is, we replace the quadratic nonlinearity of $u_{\mathrm{II}}$ by a linear damping and stochastic noise, where $\Gamma_{1}$, $\Gamma_{2}$, $\Sigma_{I}$ and $\Sigma_{\mathrm{II}}$ are positive definite symmetric matrices. The motivation of (28) is that the nonlinear self-interacting terms of fast variables $u_{\mathrm{II}}$ are responsible for the chaotic sensitive dependence on small perturbations and do not require a more detailed description if their effect on the coarse-grained dynamics for the climate variables alone is the main objective. On the other hand, the quadratic nonlinear interactions between $u_{I}$ and $u_{\mathrm{II}}$ are retained.

Note that in the second step of the MTV strategy, standard techniques of averaging and adiabatic elimination of fast modes in stochastic equations are applied, assuming that ϵ≪1. Here, we assume ϵ is a finite small value and thus do not apply the second step of the MTV strategy.

Now, inserting (28) into (26) and (27) yields

(29) $$\begin{array}{cc} du_{I} & =\left[\left(L_{11}+D_{11}\right)u_{I}+\left(L_{12}^{\prime }+D_{12}\right)u_{\mathrm{II}}+B_{11}^{1}\left(u_{I},u_{I}\right)+B_{12}^{1}\left(u_{I},u_{\mathrm{II}}\right)+F_{1}\left(t\right)\right]dt+\Sigma_{I}^{\prime }dW_{I}\left(t\right), \end{array}$$

(30) $$\begin{array}{cc} du_{\mathrm{II}} & =\left[\left(L_{22}^{\prime }+D_{22}\right)u_{\mathrm{II}}+\left(L_{21}+D_{21}\right)u_{I}+B_{11}^{2}\left(u_{I},u_{I}\right)+B_{12}^{2}\left(u_{I},u_{\mathrm{II}}\right)+F_{2}\left(t\right)\right]dt+\Sigma_{\mathrm{II}}^{\prime }W_{\mathrm{II}}\left(t\right), \end{array}$$

where $L_{12}^{\prime }=L_{12}-\Gamma_{1}/\epsilon$, $L_{22}^{\prime }=L_{22}-\Gamma_{2}/\epsilon$, $\Sigma_{I}^{\prime }=\Sigma_{I}/\sqrt{\epsilon }$ and $\Sigma_{\mathrm{II}}^{\prime }=\Sigma_{\mathrm{II}}/\sqrt{\epsilon }$. Clearly, (29) and (30) belongs to the conditional Gaussian framework. Notably, if the nonlinear terms in (29) and (30) satisfy the conditions in Section 3.3, then (29) and (30) becomes a physics-constrained nonlinear model.

## 4. A Gallery of Examples of Conditional Gaussian Systems

This section includes various conditional Gaussian complex nonlinear dynamical systems and their applications.

### 4.1. Physics-Constrained Nonlinear Low-Order Stochastic Models

#### 4.1.1. The Noisy Versions of Lorenz Models

Lorenz proposed three famous models in 1963, 1984 and 1996. These models are widely used as simplified models to mimic the turbulent and chaotic behavior and as test models for data assimilation and prediction in atmosphere and ocean science. By incorporating noise and other small-scale parameterizations, these models all belong to the conditional Gaussian family.

**1. The Noisy Lorenz 63 (L-63) Model**

(31) $$\begin{array}{cc} dx & =\sigma \left(y-x\right)dt+\sigma_{x}dW_{x},\\ dy & =\left(x(\rho -z)-y\right)dt+\sigma_{y}dW_{y},\\ dz & =\left(xy-\beta z\right)dt+\sigma_{z}dW_{z}. \end{array}$$

The deterministic L-63 model ($\sigma_{x}=\sigma_{y}=\sigma_{z}=0$ in (31)) is proposed by Lorenz in 1963 [34]. It is a simplified mathematical model for atmospheric convection. The equations relate the properties of a two-dimensional fluid layer uniformly warmed from below and cooled from above. In particular, the equations describe the rate of change of three quantities with respect to time: *x* is proportional to the rate of convection, *y* to the horizontal temperature variation, and *z* to the vertical temperature variation. The constants σ, ρ, and β are system parameters proportional to the Prandtl number, Rayleigh number, and certain physical dimensions of the layer itself [111]. The L-63 model is also widely used as simplified models for lasers, dynamos, thermosyphons, brushless direct current DC motors, electric circuits, chemical reactions and forward osmosis [112,113,114,115,116,117,118]. the noisy version of the L-63 includes more turbulent and small-scale features and their interactions with the three large scale variables while retains the characteristics in the original L-63. The noisy L-63 model is a conditional Gaussian system (1) and (2) with $u_{I}=x$ and $u_{\mathrm{II}}={\left(y,z\right)}^{T}$. It also belongs to the conditional Gaussian family with $u_{I}={\left(y,z\right)}^{T}$ and $u_{\mathrm{II}}=x$.

**Figure 1:**
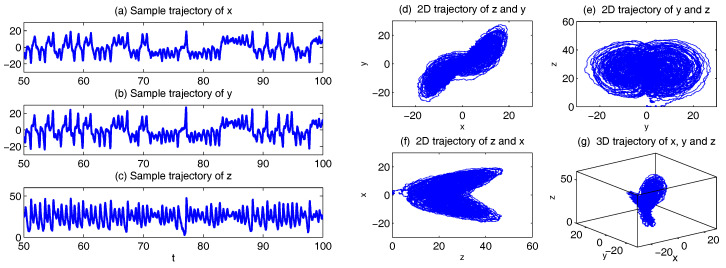
Sample trajectories of the noisy L-63 model (31) with parameters ρ = 28, σ = 10, β = 8/3, $\sigma_{x}=\sigma_{y}=\sigma_{z}=5$. (**a**–**c**) 1D trajectories of *x*, *y* and *z*, respectively; (**d**–**f**) 2D trajectories of (x,y), (y,z) and (z,x); (**g**) 3D trajectory of (x,y,z).

In Figure 1, we show some sample trajectories of the noisy L-63 model (31) with the typical values that Lorenz used [34],

(32) ρ=28,σ=10,β=8/3,

Together with a moderate noise level

(33) $$\sigma_{x}=\sigma_{y}=\sigma_{z}=5.$$

In addition to the chaotic behavior and the butterfly profile, the small fluctuations due to the noise are also clearly observed in these trajectories.

**2. The Noisy Lorenz 96 (L-96) and Two-Layer L-96 Models**

The original L-96 model with noise is given by

(34) $$du_{i}=\left(u_{i-1}\left(u_{i+1}-u_{i-2}\right)-{\bar{d}}_{i}u_{i}+F\right)dt+\sigma_{u}dW_{u_{i}}.$$

The model can be regarded as a coarse discretization of atmospheric flow on a latitude circle with complicated wave-like and chaotic behavior. It schematically describes the interaction between small-scale fluctuations with larger-scale motions. However, the noisy L-96 model in (34) usually does not have the conditional Gaussian structure unless a careful selection of a subset of $u_{i}$ for $u_{\mathrm{II}}$. Nevertheless, some two-layer L-96 models do belong to conditional Gaussian framework. These two-layer L96 models are conceptual models in geophysical turbulence that includes the interaction between small-scale fluctuations in the second layer with the larger-scale motions. They are widely used as a testbed for data assimilation and parameterization in numerical weather forecasting [36,40,119,120]. One natural choice of the two-layer L-96 model is the following [36]:

(35) $$\begin{array}{cc} du_{i} & =\left(u_{i-1}\left(u_{i+1}-u_{i-2}\right)+\sum_{j=1}^{J}\gamma_{i,j}u_{i}v_{i,j}-{\bar{d}}_{i}u_{i}+F\right)dt+\sigma_{u}dW_{u_{i}},\;i=1,\ldots ,I,\\ dv_{i,j} & =\left(-d_{v_{i,j}}v_{i,j}-\gamma_{j}u_{i}^{2}\right)dt+\sigma_{i,j}dW_{v_{i,j}},\;j=1,\ldots ,J. \end{array}$$

Linking the general conditional Gaussian framework (1) and (2) with the two-layer L-96 model in (35), $u_{I}=\left\{u_{i}\right\}$ represents the large-scale motions while $u_{\mathrm{II}}=\left\{v_{i,j}\right\}$ involves the small-scale variables.

**Figure 2:**
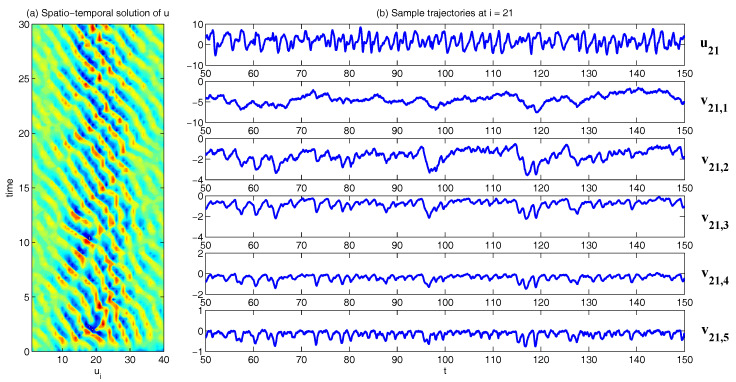
Simulations of the two-layer L-96 model in (35) with inhomogeneous parameters (36). Here, F=5. (**a**) spatio-temporal evolution of the large-scale variable $u_{i}$; (**b**) time series of $u_{i}$ and $v_{i,j}$ at i=21; from top to bottom: larger to smaller scales with increasing intermittency.

**Figure 3:**
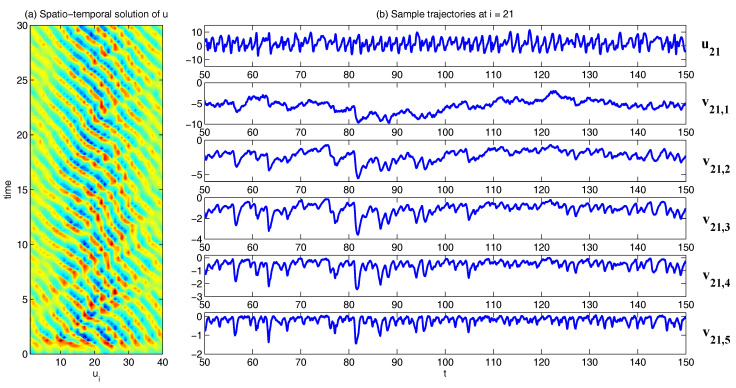
Simulations of the two-layer L-96 model in (35) with inhomogeneous parameters (36). Here, F=8. (**a**) spatio-temporal evolution of the large-scale variable $u_{i}$; (**b**) time series of $u_{i}$ and $v_{i,j}$ at i=21; from top to bottom: larger to smaller scales with increasing intermittency.

**Figure 4:**
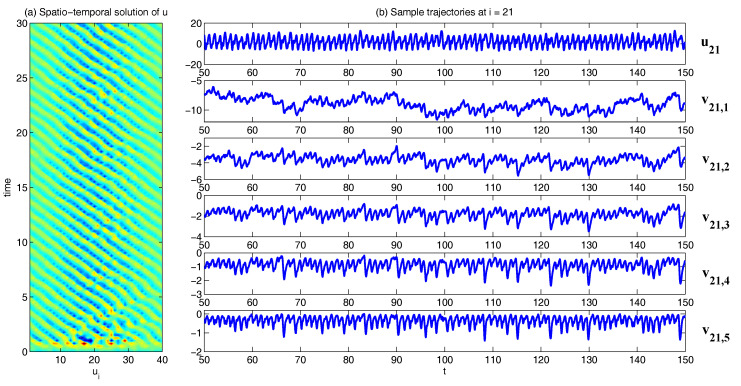
Simulations of the two-layer L-96 model in (35) with inhomogeneous parameters (36). Here, F=16. (**a**) spatio-temporal evolution of the large-scale variable $u_{i}$; (**b**) time series of $u_{i}$ and $v_{i,j}$ at i=21; from top to bottom: larger to smaller scales with increasing intermittency.

In Figure 2, Figure 3 and Figure 4, numerical simulations of the two-layer L-96 model in (35) with I=40 and J=5 are shown. Here, the damping and coupling coefficients are both functions of space with

(36) $${\bar{d}}_{i}=1+0.7\cos\left(2\pi i/J\right),\;\mathrm{and}\;\gamma_{i,j}:=\gamma_{i}=0.1+0.25\cos\left(2\pi i/J\right).$$

These mimic the situation that the damping and coupling above the ocean are weaker than those above the land since the latter usually have stronger friction or dissipation. Therefore the model is inhomogeneous and the large-scale wave patterns over the ocean are more significant, where a westward propagation is clearly seen in all these figures (Panel (a)). The other parameters are as follows:

(37) $$\begin{array}{c} \sigma_{u}=1,\;\sigma_{v_{i,1}}=0.5,\;\sigma_{v_{i,2}}=0.2,\;\sigma_{v_{i,3}}=\sigma_{v_{i,4}}=\sigma_{v_{i,5}}=0.1.\\ d_{v_{i,1}}=0.2,\;d_{v_{i,2}}=0.5,\;d_{v_{i,3}}=1,\;d_{v_{i,4}}=2,\;d_{v_{i,5}}=5, \end{array}$$

which imply that the damping time is shorter in the smaller scales. The model in (35) has many desirable properties as in more complicated turbulent systems. Particularly, the smaller scales are more intermittent (Panel (b)) with stronger fat tails in PDFs. Different constant forcing F=5,8 and 16 are adopted in Figure 2, Figure 3 and Figure 4, which result in various chaotic behavior for the system. With the forcing increase, the oscillation patterns in *u* become more regular while the small scale variables at each fixed grid point show more turbulent behavior.

**3. The Noisy Lorenz 84 (L-84) Model**

The noisy L-84 model is an extremely simple analogue of the global atmospheric circulation [121,122], which has the following form [35,123]:

(38) $$\begin{array}{cc} dx & =\left(-(y^{2}+z^{2})-a\left(x-f\right)\right)dt+\sigma_{x}dW_{x},\\ dy & =\left(-bxz+xy-y+g\right)dt+\sigma_{y}dW_{y},\\ dz & =\left(bxy+xz-z\right)dt+\sigma_{z}dW_{z}. \end{array}$$

In (38), the zonal flow *x* represents the intensity of the mid-latitude westerly wind current (or the zonally averaged meridional temperature gradient, according to thermal wind balance), and a wave component exists with *y* and *z* representing the cosine and sine phases of a chain of vortices superimposed on the zonal flow. Relative to the zonal flow, the wave variables are scaled so that $x^{2}+y^{2}+z^{2}$ is the total scaled energy (kinetic plus potential plus internal). Note that these equations can be derived as a Galerkin truncation of the two-layer quasigeostrophic potential vorticity equations in a channel.

In the L-84 model (38), the vortices are linearly damped by viscous and thermal processes. The damping time defines the time unit and a<1 is a Prandtl number. The terms xy and xz represent the amplification of the wave by interaction with the zonal flow. This occurs at the expense of the westerly current: the wave transports heat poleward, thus reducing the temperature gradient, at a rate proportional to the square of the amplitudes, as indicated by the term $-(y^{2}+z^{2})$. The terms −bxz and bxy represent the westward (if x>0) displacement of the wave by the zonal current, and b>1 allows the displacement to overcome the amplification. The zonal flow is driven by the external force af which is proportional to the contrast between solar heating at low and high latitudes. A secondary forcing *g* affects the wave, it mimics the contrasting thermal properties of the underlying surface of zonally alternating oceans and continents. When g=0 and f<1, the system has a single steady solution x=f,y=z=0, representing a steady Hadley circulation. This zonal flow becomes unstable for f>1, forming steadily progressing vortices. For g>0, the system clearly shows chaotic behavior.

Linking (38) to the general conditional Gaussian framework (1) and (2), it is clear that the zonal wind current $u_{I}=x$ is the variable for state estimation or filtering given the two phases of the large-scale vortices $u_{\mathrm{II}}=\left(y,z\right)$.

**Figure 5:**
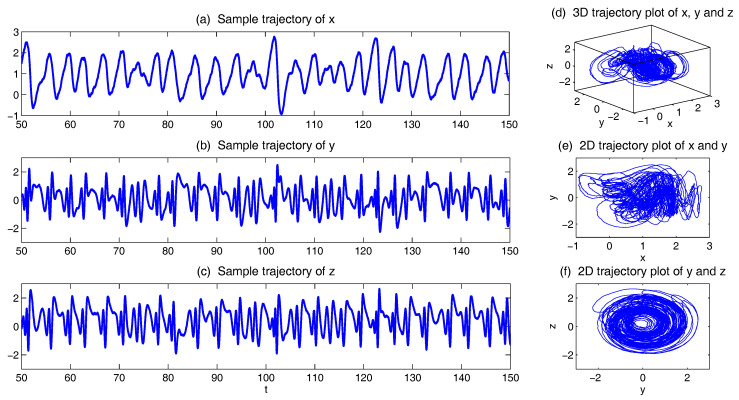
Simulations of the L-84 model (38) with parameters in (39). (**a**–**c**) sample trajectories of *x*, *y* and *z*, respectively; (**d**) 3D trajectory of (x,y,z); (**e**,**f**) 2D trajectories of (x,y) and (y,z).

In Figure 5, we show the sample trajectories of the system with the following parameters:

(39) $$a=\frac{1}{4},\;b=4,\;f=8,\;g=1,$$

which were Lorenz adopted [35]. Small noise $\sigma_{x}=\sigma_{y}=\sigma_{z}-0.1$ is also added to the system. It is clear that *y* and *z* are quite chaotic and they appear as a pair (Panels (b,c,f)). On the other hand, *x* is less turbulent and occurs in a relatively slower time scale. It plays an important role in changing both the phase and amplitude of *y* and *z*.

#### 4.1.2. Nonlinear Stochastic Models for Predicting Intermittent MJO and Monsoon Indices

**Figure 6:**
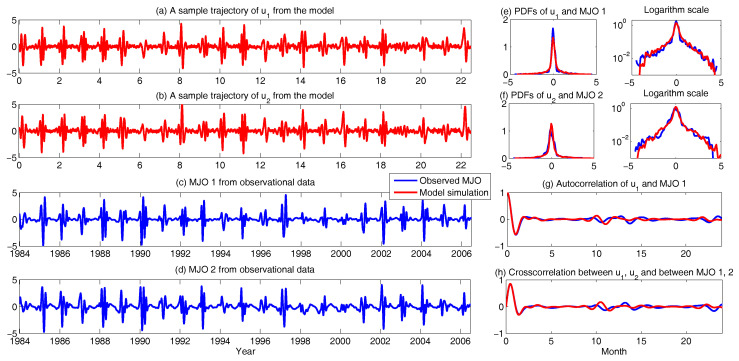
Using physics-constrained nonlinear low-order stochastic model (6) to capture the key features of the observed MJO indices. (**a**,**b**) sample trajectories from the model (6); (**c**,**d**) MJO indices from observations; (**e**,**f**) comparison of the PDFs in both linear and logarithm scales; (**g**,**h**) comparison of the autocorrelation and cross-correlation functions.

Assessing the predictability limits of time series associated with the tropical intraseasonal variability such as the the Madden–Julian oscillation (MJO) and monsoon [41,42,43] is an important topic. They yield an interesting class of low-order turbulent dynamical systems with extreme events and intermittency. For example, Panels (c,d) in Figure 6 show the MJO time series [41], measured by outgoing longwave radiation (OLR; a proxy for convective activity) from satellite data [88]. These time series are obtained by applying a new nonlinear data analysis technique, namely nonlinear Laplacian spectrum analysis [124], to these observational data. To describe and predict such intermittent time series, the following model is developed:

(40) $$\begin{array}{cc} du_{1} & =\left(-d_{u}\left(t\right)\;u_{1}\;+\;\gamma \;v\;u_{1}\;-\;\omega \;u_{2}\right)\;dt+\sigma_{u}\;dW_{u_{1}},\\ du_{2} & =\left(-d_{u}\left(t\right)\;u_{2}\;+\;\gamma \;v\;u_{2}\;+\;\omega \;u_{1}\right)\;dt+\sigma_{u}\;dW_{u_{2}},\\ dv & =\left(-d_{v}\;v\;-\;\gamma \;\left(u_{1}^{2}+u_{2}^{2}\right)\right)\;dt+\sigma_{v}\;dW_{v},\\ d\omega & =\left(-d_{\omega }\omega +\hat{\omega }\right)\;dt+\sigma_{\omega }\;dW_{\omega }, \end{array}$$

with

(41) $$d_{u}\left(t\right)=d_{u0}+d_{u1}\sin\left(\omega_{f}t+\phi \right).$$

Such a model is derived based on the physics-constrained nonlinear data-driven strategy [31,32]. In this model, $u_{I}=\left(u_{1},u_{2}\right)$ are the two-dimensional indices obtained from observational data while $u_{\mathrm{II}}=\left(v,\omega \right)$ are the two hidden unobserved variables representing other important underlying physical processes that interact with the observational ones. The coupled system is a conditional Gaussian one, which plays an important role here since there is no direct observational data for the two hidden processes *v* and ω. In fact, in order to obtain the initial values of *v* and ω for ensemble forecast, the data assimilation formula in (4) and (5) is used given the observational trajectories of $u_{1}$ and $u_{2}$. The parameters here are estimated via information theory. With the calibrated parameters, the sample trajectories as shown in Panels (a,b) capture all the important features as in the MJO indices from observations. In addition, the non-Gaussian PDFs (Panels (e,f)) and the correlation and cross-correlation functions (Panels (g,h)) from the model nearly perfectly match those associated with the observations. In [41], significant prediction skill of these MJO indices using the physics-constrained nonlinear stochastic model (6) was shown. The prediction based on ensemble mean can have skill even up to 40 days. In addition, the ensemble spread accurately quantify the forecast uncertainty in both short and long terms. In light of a twin experiment, it was also revealed in [41] that the model in (6) is able to reach the predictability limit of the large-scale cloud patterns of the MJO.

#### 4.1.3. A Simple Stochastic Model with Key Features of Atmospheric Low-Frequency Variability

This simple stochastic climate model [49,125] presented below is set-up in such a way that it features many of the important dynamical features of comprehensive global circulation models (GCMs) but with many fewer degree of freedom. Such simple toy models allow the efficient exploration of the whole parameter space that is impossible to conduct with GCMs:

(42) $$\begin{array}{cc} dx_{1} & =\left(-x_{2}\left(L_{12}+a_{1}x_{1}+a_{2}x_{2}\right)+d_{1}x_{1}+F_{1}+L_{13}y_{1}+b_{123}x_{2}y_{1}\right)dt+\sigma_{x_{1}}dW_{x_{1}},\\ dx_{2} & =\left(+x_{1}\left(L_{12}+a_{1}x_{1}+a_{2}x_{2}\right)+d_{2}x_{2}+F_{2}+L_{24}y_{2}+b_{213}x_{1}y_{1}\right)dt+\sigma_{x_{2}}dW_{x_{1}},\\ dy_{1} & =\left(-L_{13}x_{1}+b_{312}x_{1}x_{2}+F_{3}-\frac{\gamma_{1}}{\epsilon }y_{1}\right)dt+\frac{\sigma_{y_{1}}}{\sqrt{\epsilon }}dW_{y_{1}}.\\ dy_{2} & =\left(-L_{24}x_{2}+F_{4}-\frac{\gamma_{2}}{\epsilon }y_{2}\right)dt+\frac{\sigma_{y_{2}}}{\sqrt{\epsilon }}dW_{y_{2}}, \end{array}$$

where $b_{123}+b_{213}+b_{312}=0$. It contains a quadratic nonlinear part that conserves energy as well as a linear operator. Therefore, this model belongs to physics-constrained nonlinear stochastic model family. The linear operator includes a skew-symmetric part that mimics the Coriolis effect and topographic Rossby wave propagation, and a negative definite symmetric part that is formally similar to the dissipation such as the surface drag and radiative damping. The two variables $x_{1}$ and $x_{2}$ can be regarded as climate variables while the other two variables $y_{1}$ and $y_{2}$ become weather variables that occur in a much faster time scale when ϵ is small. In fact, the MTV strategy as described in Section 3.4 is applied to $y_{1}$ and $y_{2}$ that introduce this ϵ together with stochastic noise and damping terms. The coupling in different variables is through both linear and nonlinear terms, where the nonlinear coupling through $b_{ijk}$ produces multiplicative noise. Note that when ϵ→0, applying an explicit stochastic mode reduction results in a two-dimensional system for the climate variables [45,46]. Clearly, the 4D stochastic climate model (42) is a conditional Gaussian system with $u_{I}={\left(x_{1},x_{2}\right)}^{T}$ and $u_{\mathrm{II}}={\left(y_{1},y_{2}\right)}^{T}$.

**Figure 7:**
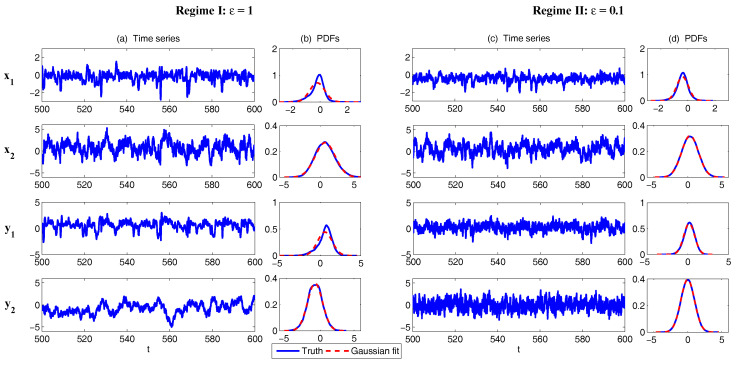
Simulations of the simple stochastic climate model (42) with parameters given by (43). (**Left**) Regime I with ϵ=1. (**Right**) Regime II with ϵ=0.1.

In Figure 7, sample trajectories and the associated PDFs in two dynamical regimes are shown. The two regimes differ by the scale separation ϵ with ϵ=1 (Regime I) and ϵ=0.1 (Regime II), respectively. The other parameters are the same in the two regimes:

(43) $$\begin{array}{c} L_{12}=1,\;L_{13}=0.5,\;L_{24}=0.5,\;a_{1}=2,\;a_{2}=1,\;d_{1}=-1,\;d_{2}=-0.4,\\ \sigma_{1}=0.5,\;\sigma_{2}=2,\;\sigma_{3}=0.5,\;\sigma_{4}=1,\;b_{123}=1.5,\;b_{213}=1.5,\;b_{312}=-3,\\ F_{1}=F_{2}=F_{3}=F_{4}=0. \end{array}$$

It is obvious that with ϵ=1, all the four variables lie in the same time scale. Both “climate variable” $x_{1}$ and “weather variable” $y_{1}$ can have intermittent behavior with non-Gaussian PDFs. On the other hand, with ϵ=0.1, a clear scale separation can be seen in the time series, where $y_{1}$ and $y_{2}$ occur in a much faster time scale than $x_{1}$ and $x_{2}$. Since the memory time due to the strong damping becomes much shorter in $y_{1}$ and $y_{2}$, the associated PDFs for these “weather variables” become Gaussian.

#### 4.1.4. A Nonlinear Triad Model with Multiscale Features

The following nonlinear triad system is a simple prototype nonlinear stochastic model that mimics structural features of low-frequency variability of GCMs with non-Gaussian features [50] and it was used to test the skill for reduced nonlinear stochastic models for the fluctuation dissipation theorem [126]:

(44) $$\begin{array}{cc} du_{1} & =\left(-\gamma_{1}u_{1}+L_{12}u_{2}+L_{13}u_{3}+Iu_{1}u_{2}+F\left(t\right)\right)\;dt+\sigma_{1}dW_{1}, \end{array}$$

(45) $$\begin{array}{cc} du_{2} & =\left(-L_{12}u_{1}-\frac{\gamma_{2}}{\epsilon }u_{2}+L_{23}u_{3}-Iu_{1}^{2}\right)dt+\frac{\sigma_{2}}{\epsilon^{1/2}}dW_{2}, \end{array}$$

(46) $$\begin{array}{cc} du_{3} & =\left(-L_{13}u_{1}-L_{23}u_{2}-\frac{\gamma_{3}}{\epsilon }u_{3}\right)\;dt+\frac{\sigma_{3}}{\epsilon^{1/2}}dW_{3}. \end{array}$$

The triad model (44)–(46) involves a quadratic nonlinear interaction between $u_{1}$ and $u_{2}$ with energy-conserving property that induces intermittent instability. On the other hand, the coupling between $u_{2}$ and $u_{3}$ is linear and is through the skew-symmetric term with coefficient $-L_{23}$, which represents an oscillation structure of $u_{2}$ and $u_{3}$. Particularly, when $L_{23}$ is large, fast oscillations become dominant for $u_{2}$ and $u_{3}$ while the overall evolution of $u_{1}$ can still be slow provided that the feedback from $u_{2}$ and $u_{3}$ is damped quickly. Such multiscale structure appears in the turbulent ocean flows described for example by shallow water equation, where $u_{1}$ stands for the geostrophically balanced part while $u_{2}$ and $u_{3}$ mimics the fast oscillations due to the gravity waves [11,122]. The large-scale forcing F(t) represents the external time-periodic input to the system, such as the seasonal effects or decadal oscillations in a long time scale [121,127]. In addition, the scaling factor ϵ plays the same role as in the 4D stochastic climate model (42) that allows a difference in the memory of the three variables. Note that the MTV strategy as described in Section 3.4 is applied to $u_{2}$ and $u_{3}$ that introduces the factor ϵ. The nonlinear triad model in (44)–(46) belongs to conditional Gaussian model family with $u_{I}={\left(x_{1},x_{2}\right)}^{T}$ and $u_{\mathrm{II}}={\left(y_{1},y_{2}\right)}^{T}$.

**Figure 8:**
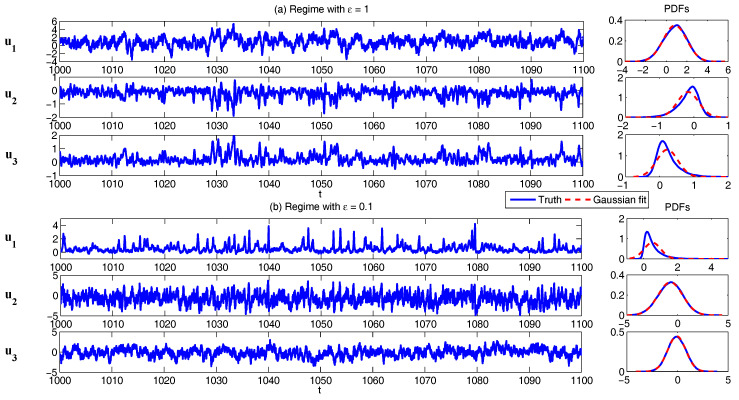
Model simulation of the triad model (44)–(46) with parameters in (47). (**a**) Regime I with ϵ=1; (**b**) Regime II with ϵ=0.1.

To understand the dynamical behavior of the nonlinear triad model (44)–(46), we show the model simulations in Figure 8 for the following two regimes:

(47) $$\begin{array}{cc} \mathrm{Regime}\;I: & \;\epsilon =1.0,\;I=1,\;\sigma_{1}=0.5,\;L_{12}=0.4,\;L_{13}=0.4,\;L_{23}=0,\\ & \;\gamma_{1}=2.5,\;\gamma_{2}=0.4,\;\gamma_{3}=0.4,\;\sigma_{2}=1.2,\;\sigma_{3}=0.8,\;F=2,\\ \mathrm{Regime}\;\mathrm{II}: & \;\epsilon =0.1,\;I=5,\;\sigma_{1}=0.5,\;L_{12}=1.0,\;L_{13}=1.0,\;L_{23}=10,\\ & \;\gamma_{1}=2.0,\;\gamma_{2}=0.1,\;\gamma_{3}=0.1,\;\sigma_{2}=1.2,\;\sigma_{3}=0.8,\;F=2. \end{array}$$

In Regime I, ϵ=1 and therefore $u_{1},u_{2}$ and $u_{3}$ occur in the same time scale. Since a large noise coefficient $\sigma_{1}=2.5$ is adopted in the dynamics of $u_{1}$, the PDF of $u_{1}$ is nearly Gaussian and the variance is large. The latter leads to a skewed PDF of $u_{2}$ due to the nonlinear feedback term $-Iu_{1}^{2}$, where the extreme events are mostly towards the negative side of $u_{2}$ due to the negative sign in front of the nonlinear term. As a consequence, the $u_{3}$ also has a skewed PDF but the skewness is positive. On the other hand, in Regime I, where ϵ=0.1, $u_{2}$ and $u_{3}$ both have a short decorrelation time and the associated PDFs are nearly Gaussian. Nevertheless, the slow variable $u_{1}$ is highly non-Gaussian due to the direct nonlinear interaction between $u_{2}$ in which $u_{2}$ plays the role of stochastic damping and it results in the intermittent instability in the signal of $u_{1}$.

#### 4.1.5. Conceptual Models for Turbulent Dynamical Systems

Understanding the complexity of anisotropic turbulence processes over a wide range of spatiotemporal scales in engineering shear turbulence [128,129,130] as well as climate atmosphere ocean science [73,121,122] is a grand challenge of contemporary science. This is especially important from a practical viewpoint because energy often flows intermittently from the smaller scales to affect the largest scales in such anisotropic turbulent flows. The typical features of such anisotropic turbulent flows are the following: (A) The large-scale mean flow is usually chaotic but more predictable than the smaller-scale fluctuations. The overall single point PDF of the flow field is nearly Gaussian whereas the mean flow pdf is sub-Gaussian, in other words, with less extreme variability than a Gaussian random variable; (B) There are nontrivial nonlinear interactions between the large-scale mean flow and the smaller-scale fluctuations which conserve energy; (C) There is a wide range of spatial scales for the fluctuations with features where the large-scale components of the fluctuations contain more energy than the smaller-scale components. Furthermore, these large-scale fluctuating components decorrelate faster in time than the mean-flow fluctuations on the largest scales, whereas the smaller-scale fluctuating components decorrelate faster in time than the larger-scale fluctuating components; (D) The PDFs of the larger-scale fluctuating components of the turbulent field are nearly Gaussian, whereas the smaller-scale fluctuating components are intermittent and have fat-tailed PDFs, in other words, a much higher probability of extreme events than a Gaussian distribution.

Denote *u* the largest variable and $\left\{v_{k}\right\}$ a family of small-scale variables. One can think of *u* as the large-scale spatial average of the turbulent dynamics at a single grid point in a more complex system and $\left\{v_{k}\right\}$ as the turbulent fluctuations at the grid point. To add a sense of spatial scale, one can also regard $v_{k}$ as the amplitude of the *k*-th Fourier cosine mode evaluated at a grid point. A hallmark of turbulence is that the large scales can destabilize the smaller scales in the turbulent fluctuations intermittently and this increased small-scale energy can impact the large scales; this key feature is captured in the conceptual models. With the above discussion, here are the simplest models with all these features, the conceptual dynamical models for turbulence [37]:

(48) $$\begin{array}{cc} du & =\left(-d_{u}u+\gamma \sum_{k=1}^{K}v_{k}^{2}+F\right)dt,\\ dv_{k} & =\left(-d_{v_{k}}v_{k}-\gamma uv_{k}\right)dt+\sigma_{v_{k}}dW_{v_{k}},\;1\leq k\leq K. \end{array}$$

Now, let us take K=5 and use the following parameters which have been tested in [37] that represent the features of turbulent flows:

(49) $$d_{u}=0.01,\;d_{v_{k}}=1+0.02k^{2},\;\frac{\sigma_{v_{k}}^{2}}{d_{v_{k}}}=\frac{0.004}{{\left(1+k\right)}^{5/3}},\;\gamma =1.5,\;F=-0.15.$$

**Figure 9:**
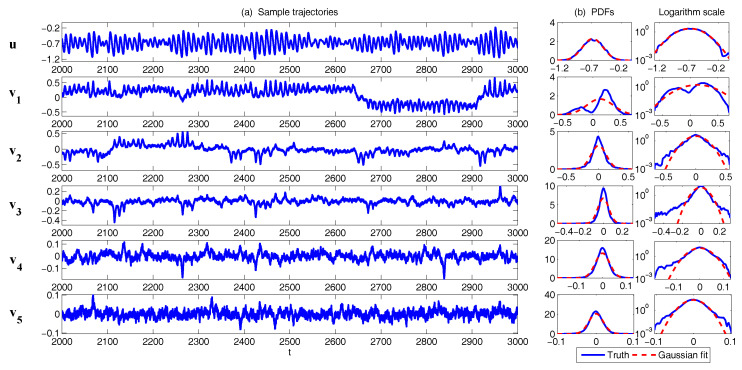
Sample trajectories (**a**) and the associated PDFs (**b**) of the conceptual turbulent model (48) with parameters in (49).

The sample trajectories and the associated PDFs are shown in Figure 9. The largest scale *u* is nearly Gaussian while more intermittent behavior is observed in the smaller scale variables. Here, the nonlinear interaction plays an important role in generating these turbulent features and the total energy in the nonlinear terms is conserved. Thus, all the features (A)–(D) above are addressed in this model. It is also clear that the conceptual turbulent model (48) is a conditional Gaussian system with $u_{I}={\left(v_{1},\ldots ,v_{5}\right)}^{T}$, $u_{\mathrm{II}}=u$.

In [36], a modified version of the conceptual model was developed:

(50) $$\begin{array}{cc} du & =\left(-d_{u}u+\sum_{k}^{K}\;\gamma_{k}\;u\;v_{k}+F\right)dt+\sigma_{u}dW_{u}, \end{array}$$

(51) $$\begin{array}{cc} dv_{k} & =\left(-d_{v_{k}}v_{k}-\gamma_{k}\;u^{2}\right)dt+\sigma_{v_{k}}dW_{v_{k}},\;k=1,\ldots ,K. \end{array}$$

**Figure 10:**
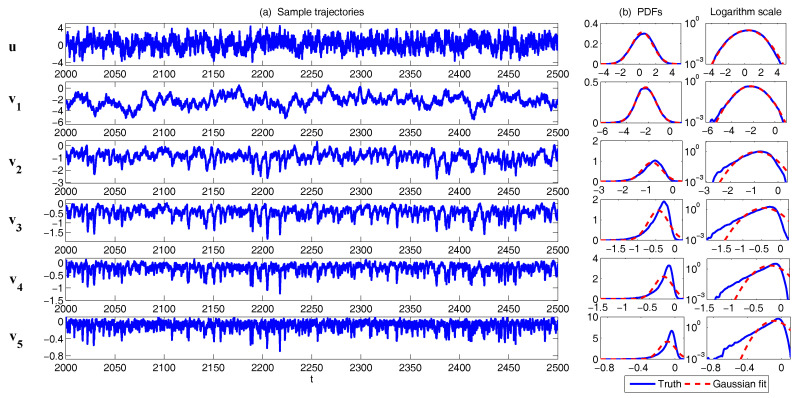
Sample trajectories (**a**) and the associated PDFs (**b**) of the modified conceptual turbulent model (50) and (51) with parameters in (52).

This modified conceptual turbulent model again fits into the conditional Gaussian framework, where $u_{I}=u$ includes the largest scale variable and $u_{\mathrm{II}}={\left(v_{1},\ldots ,v_{K}\right)}^{T}$ represents small-scale ones. The modified conceptual turbulent model (50) and (51) inherits many important features from the dynamics in (48). For example, with suitable choices of parameters, the large-scale observed variable *u* is nearly Gaussian while small-scale variables $v_{k}$ becomes more intermittent with the increasing of *k*. In addition, the small-scale turbulent flows provide feedback to large scales through the nonlinear coupling with energy-conserving property. An example is shown in Figure 10 with the following choice of parameters:

(52) $$\begin{array}{c} d_{u}=0.1,\;F=0.5,\;\sigma_{u}=2,\;\gamma_{k}=0.25,\\ \;d_{v_{k}}=\left\{0.2,0.5,1,2,5\right\},\;\sigma_{v_{k}}=\left\{0.5,0.2,0.1,0.1,0.1\right\},\;k=1,\ldots ,5. \end{array}$$

#### 4.1.6. A Conceptual Model of the Coupled Atmosphere and Ocean

In [39], the signatures of feedback between the atmosphere and the ocean are studied with a simple coupled model, which can be used to exam the effects of oceanic variability and seasonality.

The atmosphere component is the Lorenz 1984 model (38) discussed in Section 4.1.1, except that the forcing has an explicit form

$$f\left(t\right)=f_{0}+f_{1}\cos\omega t,$$

which represents seasonal effect. Therefore, the atmosphere model reads

(53) $$\begin{array}{cc} dx & =\left(-(y^{2}+z^{2})-a\left(x-f\left(t\right)\right)\right)dt+\sigma_{x}dW_{x},\\ dy & =\left(-bxz+xy-y+g\right)dt+\sigma_{y}dW_{y},\\ dz & =\left(bxy+xz-z\right)dt+\sigma_{z}dW_{z}. \end{array}$$

Again, *x* represents the amplitude of the zonally averaged meridional temperature gradient while *y* and *z* denote the amplitudes of the cosine and sine longitudinal phases of a chain of large-scale waves. The poleward heat transport is achieved by the eddies at a rate proportional to $y^{2}+z^{2}$, and this heat transport reduces the zonally averaged temperature gradient. The term f(t) represents the zonally averaged forcing due to the equator-pole difference in solar heating and it varies on a seasonal timescale.

On the other hand, the oceanic module simulates the wind-driven circulation in a basin that occupies a fraction *r* of the longitudinal extent of the atmosphere. Its dynamics are described by a set of four ordinary differential equations, namely,

(54) $$\begin{array}{cc} dp & =-(\psi_{r}^{2}+\psi_{i}^{2})pdt,\\ dq & =0,\\ d\psi_{r} & =(-\sigma \psi_{r}-\Omega \psi_{i})dt,\\ d\psi_{i} & =(\Omega \psi_{r}-\sigma \psi_{i})dt. \end{array}$$

Here, *p* represents the zonally averaged meridional temperature gradient at the sea surface, while *q* represents the basin-averaged sea surface temperature. The poleward heat transport is achieved by a large-scale flow, at a rate proportional to $\psi_{r}^{2}+\psi_{i}^{2}$. The average temperature *Q* is conserved in the absence of any coupling with the atmosphere. The transport is represented by two phases of the streamfunction, $\psi_{r}$ and $\psi_{i}$. The streamfunction undergoes damped oscillations with a period, 2π/Ω, of 5.3 y and a decay time, $\sigma^{-1}$, of 17 y. This damped oscillation is the only source of internal variability in the ocean and is due to the intrinsic decadal variability of the wind-driven circulation. Note that the equations for the two phases of the streamfunction can be derived as a Galerkin truncation of the one-and-a-half-layer quasigeostrophic potential vorticity equation for long linear Rossby waves. It is suggested in [131,132] that basin modes with decadal frequencies can be excited by stochastic atmospheric forcing and represent a resonant response of the ocean. This model essentially assumes that the intrinsic decadal variability of the ocean wind-driven circulation is described by one such mode. For weak flow, the wind-driven gyres reduce the zonally averaged north-south temperature gradient in a basin.

The feedback between the ocean and the atmosphere are constructed so as to conserve total heat in the air–sea exchange. The coupled atmosphere-ocean model reads:

(55) $$\begin{array}{cc} dx & =\left(-(y^{2}+z^{2})-a\left(x-f\left(t\right)\right)\underline{\;+\;rm(p-x-\gamma )}\right)dt+\sigma_{x}dW_{x},\\ dy & =\left(-bxz+xy\;-\;y+g\underline{\;+\;rm(q-y)}\right)dt+\sigma_{y}dW_{y},\\ dz & =\left(bxy+xz\;-\;z\right)dt+\sigma_{z}dW_{z},\\ dp & =\left(-np\underline{\;+\;mc^{-1}\left(x-p+\gamma \right)}\right)dt+\sigma_{p}dW_{p},\\ dq & =\underline{\;mc^{-1}\left(y-q\right)}+\sigma_{q}dW_{q},\\ d\psi_{r} & =\left(-\sigma \psi_{r}-\Omega \psi_{i}\underline{\;+\;\alpha_{r}x+\beta_{r}y}\right)dt+\sigma_{\phi_{r}}dW_{\phi_{r}},\\ d\psi_{i} & =\left(\Omega \psi_{r}-\sigma \psi_{i}\underline{\;+\;\alpha_{i}x+\beta_{i}y}\right)dt+\sigma_{\phi_{i}}dW_{\phi_{i}}, \end{array}$$

where the terms with underlines represent the coupling between atmosphere and ocean. In (55), the term $\psi_{r}^{2}+\psi_{i}^{2}$ on the right-hand side of the evolution of *p* is parameterized by a constant *n* such that the ocean part become a linear model. Therefore, (55) includes a nonlinear atmosphere model and a linear ocean model. The coupling is through linear terms. Thus, treating the atmosphere variables as $u_{I}=\left(x,y,z\right)$ and the ocean variables as $u_{\mathrm{II}}=\left(p,q,\psi_{r},\psi_{i}\right)$, the coupled system (55) belongs to conditional Gaussian framework. It is also obvious that the coupled model belongs to the physics-constrained regression model family.

In (55), the air–sea heat fluxes are proportional to the difference between the oceanic and the atmospheric temperature: these are the terms m(p−x) and m(q−y). The bulk transfer coefficient, *m*, is assumed to be constant. In the atmospheric model, this term needs to be multiplied by the fraction of earth covered by ocean, *r*. In the oceanic model, the air–sea flux is divided by *c*, which is the ratio of the vertically integrated heat capacities of the atmosphere and the ocean. The constant γ represents the fraction of solar radiation that is absorbed directly by the ocean. There is no heat exchange between the atmospheric standing wave *z* and the ocean because *z* represents the sine phase of the longitudinal eddies and has zero zonal mean across the ocean. A feedback between *z* and the ocean would appear if we added an equation for the longitudinal temperature gradient of the ocean. The effect of the wind stress acting on the ocean is represented as a linear forcing proportional to *x* and *y* in the equations for the streamfunction, ψ. The coupling constants $\alpha_{r}$, $\alpha_{i}$, $\beta_{r}$, and $\beta_{i}$ are chosen to produce realistic values for the oceanic heat transport. Detailed discussions of the parameter choices and model behaviors in different dynamical regimes are shown in [39].

#### 4.1.7. A Low-Order Model of Charney–DeVore Flows

The concept of multiple equilibria in a severely truncated “low-order” image of the atmospheric circulation was proposed by Charney and DeVore [133] (the CdV model). The simplest CdV model describes a barotropic zonally unbounded flow over a sinusoidal topography in a zonal channel with quasigeostrophic dynamics. The vorticity balance of such a flow

(56) $$\frac{\partial }{\partial t}\nabla^{2}\Psi +u\cdot \nabla \left[\nabla^{2}\Psi +\beta y+\frac{f_{0}b}{H}\right]=R\nabla^{2}\left(\Psi^{*}-\Psi \right)$$

needs an additional constraint to determine the boundary values of the streamfunction Ψ on the channel walls. The vorticity concept has eliminated the pressure field and its reconstruction in a multiconnected domain requires the validity of the momentum balance, integrated over the whole domain,

(57) $$\frac{\partial U}{\partial t}=R\left(U^{*}-U\right)+\frac{f_{0}}{H}\langle b\frac{\partial \Psi }{\partial x}\rangle .$$

Here, *U* is the zonally and meridionally averaged zonal velocity and $R\nabla^{2}\Psi^{*}=-R\partial U^{*}/\partial y$ is the vorticity and $RU^{*}$ the zonal momentum imparted into the system.

The depth of the fluid is H−b and the topography height b is taken sinusoidal, $b=b_{0}\cos\;Kx\;\sin\;Ky$ with K=2πn/L, where *L* is the length and L/2 the width of the channel. A heavily truncated expansion

$$\Psi =-Uy+\frac{1}{K}\left[A\;\cos\;Kx+B\;\sin\;Kx\right]\sin\;Ky$$

represents the flow in terms of the zonal mean *U* and a wave component with sine and cosine amplitudes *A* and *B*. It yields the low-order model [29,133]:

(58) $$\begin{array}{cc} dU & =\left(R\left(U^{*}\;-\;U\right)+\frac{1}{4}\delta B\right)dt+\sigma_{U}dW_{U},\\ dA & =\left(-KB(U-c_{R})-RA\right)dt+\sigma_{A}dW_{A},\\ dB & =\left(KA\left(U-c_{R}\right)-\frac{1}{2}\delta U-RB\right)dt+\sigma_{B}dW_{B}, \end{array}$$

where $c_{R}=\beta /2K^{2}$ is the barotropic Rossby wave speed and $\delta =f_{0}b_{0}/H$. Apparently, (58) belongs to the conditional Gaussian framework when the zonal mean flow is treated as $u_{I}=U$ while the two wave components belong to $u_{\mathrm{II}}={\left(A,B\right)}^{T}$. This model also belongs to physics-constrained nonlinear modeling family.

Without the stochastic noise, three equilibria are found if $U^{*}$ is well above $c_{R}$. The three possible steady states can be classified according to the size of the mean flow *U* compared to the wave amplitudes: (a) The high zonal index regime is frictionally controlled, the flow is intense and the wave amplitude is low; (b) The low zonal index regime is controlled by form stress, the mean flow is weak and the wave is intense; (c) The intermediate state is transitional, it is actually unstable to perturbations. This “form stress instability” works obviously when the slope of the resonance curve is below the one associated with friction, i.e., $\partial (RU-\frac{1}{4}\delta B\left[U\right])/\partial U>0$, so that a perturbation must run away from the steady state.

#### 4.1.8. A Paradigm Model for Topographic Mean Flow Interaction

A barotropic quasi-geostrophic equation with a large scale zonal mean flow can be used to solve topographic mean flow interaction. The full equations are given as follows [2]:

(59) $$\begin{array}{cc} \frac{\partial q}{\partial t} & =-\nabla^{⊥}\psi \cdot \nabla q-u\left(t\right)\frac{\partial q}{\partial x}-\beta \frac{\partial \psi }{\partial x},\\ q & =\Delta \psi +h,\\ \frac{du}{dt} & =\frac{1}{4\pi^{2}}\int h\frac{\partial \psi }{\partial x}. \end{array}$$

Here, *q* and ψ are the small-scale potential vorticity and stream function, respectively. The large scale zonal mean flow is characterized by u(t) and the topography is defined by function h=h(x,y). The parameter β is associated with the β-plane approximation to the Coriolis force. The domain considered here is a periodic box ${\left[0,2\pi \right]}^{2}$.

Now, we construct a set of special solutions to (59), which inherit the nonlinear coupling of the small-scale flow with the large-scale mean flow via topographic stress. Consider the following Fourier decomposition:

(60) $$\psi \left(\vec{x},t\right)=\sum_{k\neq 0}\psi_{k}\left(t\right)e^{ik\vec{l}\cdot \vec{x}},\;h\left(\vec{x}\right)=\sum_{k\neq 0}h_{k}e^{ik\vec{l}\cdot \vec{x}},$$

where $\vec{l}=\left(l_{x},l_{y}\right)$ and $\vec{x}=\left(x,y\right)$. By assuming $h_{0}=0$ and inserting (60) into (59), we arrive at the layered topographic equations in Fourier form:

$$\begin{array}{cc} \frac{d\psi_{k}}{dt} & =ikl_{x}\left(\frac{\beta }{k^{2}{\left|\vec{l}\right|}^{2}}-u\right)\psi_{k}+i\frac{kl_{x}}{k^{2}{\left|\vec{l}\right|}^{2}}h_{k}u,\\ \frac{du}{dt} & =-il_{x}\sum_{k\neq 0}kh_{k}\psi_{k}^{*}, \end{array}$$

where $\psi_{k}^{*}=\psi_{-k}$ and $h_{k}^{*}=h_{-k}$. Consider a finite Fourier truncation. By adding extra damping and stochastic noise for compensating the information truncated in the finite Fourier decomposition in $\psi_{k}$,

$$\begin{array}{cc} d\psi_{k} & =\left(-d_{k}\psi_{k}+ikl_{x}\left(\frac{\beta }{k^{2}{\left|\vec{l}\right|}^{2}}-u\right)\psi_{k}+i\frac{kl_{x}}{k^{2}{\left|\vec{l}\right|}^{2}}h_{k}u\right)dt+\sigma_{k}dW_{k},\;\left|k\right|\leq K,\\ du & =\left(-il_{x}\sum_{k\neq 0}kh_{k}\psi_{k}^{*}\right)dt. \end{array}$$

We arrive at a conditional Gaussian system with $u_{I}=\left\{\psi_{k}\right\}$ and $u_{\mathrm{II}}=u$.

In [2], very rich chaotic dynamics are reached with two-layer topographic models. Here as a concrete example, we consider a single layer topography in the zonal direction $\vec{l}=\left(1,0\right)$ [32],

$$\begin{array}{cc} \psi (x,t) & =\psi_{1}\left(t\right)e^{ix}+\psi_{-1}\left(t\right)e^{-ix},\\ h(x) & =H\left(\cos(x)+\sin(x)\right)=h_{1}e^{-ix}+h_{-1}e^{-ix}, \end{array}$$

where $h_{1}=H/2-H/2i$ and *H* denotes the topography amplitude. Choose

$$\psi_{1}\left(t\right)=\frac{1}{2}\left(b(t)-ia(t)\right)\;and\;\psi_{-1}=\psi_{1}^{*}$$

and rotate the variables (a,b) counterclockwise by 45° to coordinate $\left(v_{1},v_{2}\right)$. We arrive at

(61) $$\begin{array}{cc} du & =\omega v_{1}dt+\sigma_{u}dW_{u},\\ dv_{1} & =\left(-2\omega u-\beta v_{2}+uv_{2}\right)dt,\\ dv_{2} & =(\beta v_{1}-uv_{1})dt, \end{array}$$

where ω= $H/\sqrt{2}$. This model is similar to the Charney and Devore model for nonlinear regime behavior without dissipation and forcing. Then, with additional damping and noise in $v_{1}$ and $v_{2}$ approximating the interaction with the truncated Rossby wave modes, we have the following system:

(62) $$\begin{array}{cc} du & =\omega v_{1}dt,\\ dv_{1} & =\left(-d_{v_{1}}v_{1}-2\omega u-\beta v_{2}+uv_{2}\right)dt+\sigma_{v_{1}}dW_{v_{1}},\\ dv_{2} & =\left(-d_{v_{2}}v_{2}+\beta v_{1}-uv_{1}\right)dt+\sigma_{v_{2}}dW_{v_{2}}. \end{array}$$

**Figure 11:**
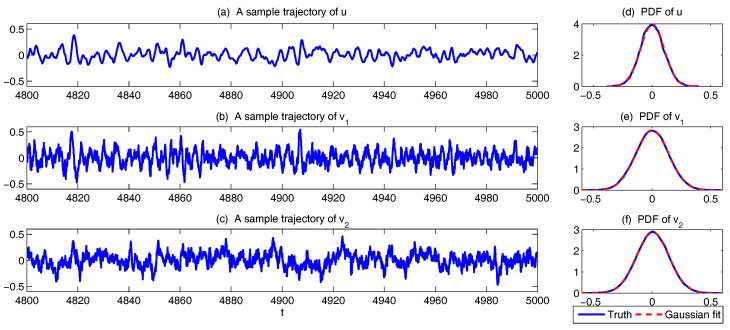
Sample trajectories (**a**–**c**) and the associated PDFs (**d**–**f**) of the single layer topography model (62) with parameters in (63). Note that the invariant measure is Gaussian.

Linear stability is satisfied for $v_{1},v_{2}$ while there is only neutral stability of *u*. The system in (62) satisfies the conditional Gaussian framework where $u_{I}={\left(v_{1},v_{2}\right)}^{T}$ and $u_{\mathrm{II}}=u$. Notably, this model also belongs to the physics-constrained nonlinear model family. One interesting property of this model is that, if the invariant measure exists, then, despite the nonlinear terms, the invariant measure is Gaussian. The validation of this argument can be easily reached following [31]. A numerical illustration is shown Figure 11 with the following parameters:

(63) $$\omega =1,\;d_{v_{1}}=d_{v_{2}}=1,\;\beta =0.5,\;\sigma_{v_{1}}=0.2,\;\sigma_{v_{2}}=0.2.$$

Note that, if stochastic noise is also added in the evolution equation of *u*, then the system (62) also belongs to the conditional Gaussian model family with $u_{I}=u$ and $u_{\mathrm{II}}={\left(v_{1},v_{2}\right)}^{T}$.

### 4.2. Stochastically Coupled Reaction–Diffusion Models in Neuroscience and Ecology

#### 4.2.1. Stochastically Coupled FitzHugh–Nagumo (FHN) Models

The FitzHugh–Nagumo model (FHN) is a prototype of an excitable system, which describes the activation and deactivation dynamics of a spiking neuron [57]. Stochastic versions of the FHN model with the notion of noise-induced limit cycles were widely studied and applied in the context of stochastic resonance [134,135,136,137]. Furthermore, its spatially extended version has also attracted much attention as a noisy excitable medium [138,139,140,141].

One common representation of the stochastic FHN model is given by

(64) $$\begin{array}{cc} \epsilon du & =\left(f_{1}\left(u\right)+c_{1}v\right)dt+\sqrt{\epsilon }\delta_{1}dW_{u},\\ dv & =\left(f_{2}\left(u\right)+c_{2}v+s\left(t\right)\right)dt+\delta_{2}dW_{v}, \end{array}$$

where the time scale ratio ϵ is much smaller than one (e.g., $\epsilon \approx 10^{-2}$), implying that u(t) is the fast and v(t) is the slow variable. The coupled FHN system in (64) is obviously a conditional Gaussian system with $u_{I}=u$ and $u_{\mathrm{II}}=v$. The nonlinear function $f_{1}\left(u\right)$ is one of the nullclines of the deterministic system. A common choice for this function is

(65) $$f_{1}\left(u\right)=u-cu^{3},$$

where the parameter *c* is either 1 or 1/3. On the other hand, $f_{2}\left(u\right)$ is usually a linear function of *u*. In (64), s(t) is an external source and it can be a time-dependent function. In the following, we set s(t) to be a constant external forcing for simplicity. Diffusion term is typically imposed in the dynamics of *u*. Thus, with these choices, a simple stochastically coupled spatial-extended FHN model is given by

(66) $$\begin{array}{cc} \epsilon du & =\left(d_{u}\nabla^{2}u+u-\frac{1}{3}u^{3}-v\right)dt+\sqrt{\epsilon }\delta_{1}dW_{u},\\ dv & =\left(u+a\right)dt+\delta_{2}dW_{v}. \end{array}$$

With $\delta_{1}$ and $\delta_{2}$, the model in (66) contains the model families with both coherence resonance and self-induced stochastic resonance [142]. Applying a finite difference discretization to the diffusion term in (66), we arrive at the FHN model in the lattice form

(67) $$\begin{array}{cc} \epsilon du_{i} & =\left(d_{u}\left(u_{i+1}+u_{i-1}-2u_{i}\right)+u_{i}-\frac{1}{3}u_{i}^{3}-v_{i}\right)dt+\sqrt{\epsilon }\delta_{1}dW_{u_{i}},\\ dv_{i} & =\left(u_{i}+a\right)dt+\delta_{2}dW_{v_{i}},\;i=1,\ldots ,N. \end{array}$$

Note that the parameter a>1 is required in order to guarantee that the system has a global attractor in the absence of noise and diffusion. The random noise is able to drive the system above the threshold level of global stability and triggers limit cycles intermittently. The model behavior of (67) in various dynamical regimes has been studied in [36]. The model can show both strongly coherent and irregular features as well as scale-invariant structure with different choices of noise and diffusion coefficients.

There are several other modified versions of the FHN model that are widely used in applications. One that appears in the thermodynamic limit of an infinitely large ensemble is the so-called globally coupled FHN model

(68) $$\begin{array}{cc} \epsilon du_{i} & =\left(d_{u}\left(u_{i+1}+u_{i-1}-2u_{i}\right)+u_{i}-\frac{1}{3}u_{i}^{3}+m\left(\bar{u}-u_{i}\right)-v_{i}\right)dt+\sqrt{\epsilon }\delta_{1}dW_{u_{i}},\\ dv_{i} & =\left(u_{i}+a\right)dt+\delta_{2}dW_{v_{i}},\;i=1,\ldots ,N, \end{array}$$

where $\bar{u}=\frac{1}{N}\sum_{i=1}^{N}u_{i}$. Different from the model in (67) where each $u_{i}$ is only directly coupled to its two nearest neighbors $u_{i-1}$ and $u_{i+1}$, each $u_{i}$ in the globally coupled FHN model (68) is directly affected by all $u_{j},j=1,\ldots ,N$. In [57], various closure methods are developed to solve the time evolution of the statistics associated with the globally coupled FHN model (68).

Another important modification of the FHN model is to include the colored noise into the system as suggested by [57,143]. For example, the constant *a* on the right-hand side of (67) can be replaced by an OU process [143] that allows the memory of the additive noise. Below, by introducing a stochastic coefficient γ in front of the linear term *u*, a stochastically coupled FHN model with multiplicative noise is developed. The model reads

(69) $$\begin{array}{cc} \epsilon du_{i} & =\left(d_{u}\left(u_{i+1}+u_{i-1}-2u_{i}\right)+u_{i}-\frac{1}{3}u_{i}^{3}-v_{i}\right)dt+\sqrt{\epsilon }\delta_{1}dW_{u_{i}},\\ dv_{i} & =\left(\gamma_{i}u_{i}+a\right)dt+\delta_{2}dW_{v_{i}},\\ d\gamma_{i} & =-d_{\gamma_{i}}\left(\gamma_{i}-{\hat{\gamma }}_{i}\right)dt+\sigma_{\gamma_{i}}dW_{\gamma_{i}},\;i=1,\ldots ,N. \end{array}$$

The stochastically coupled FHN model with multiplicative noise belongs to the conditional Gaussian framework with $u_{I}={\left(u_{1},\ldots ,u_{N}\right)}^{T}$ and $u_{\mathrm{II}}={\left(v_{1},\gamma_{1},\ldots ,v_{N},\gamma_{N}\right)}^{T}$.

To provide intuition about the dynamical behavior of the stochastically coupled FHN model with multiplicative noise (69), we show the time series of the model with n=1, namely there is no diffusion term and the coupled model is three-dimensions. The three-dimensional model with no diffusion reads,

(70) $$\begin{array}{cc} \epsilon du & =\left(u-\frac{1}{3}u^{3}-v\right)dt+\sqrt{\epsilon }\delta_{1}dW_{u},\\ dv & =\left(\gamma u+a\right)dt+\delta_{2}dW_{v},\\ d\gamma & =-d_{\gamma }\left(\gamma -\hat{\gamma }\right)dt+\sigma_{\gamma }dW_{\gamma }, \end{array}$$

with the following parameters

(71) $$\epsilon =0.01,\;\delta_{1}=0.2,\;\delta_{2}=0.4,\;a=1.05,\;d_{\gamma }=1,\;\hat{\gamma }=1.$$

**Figure 12:**
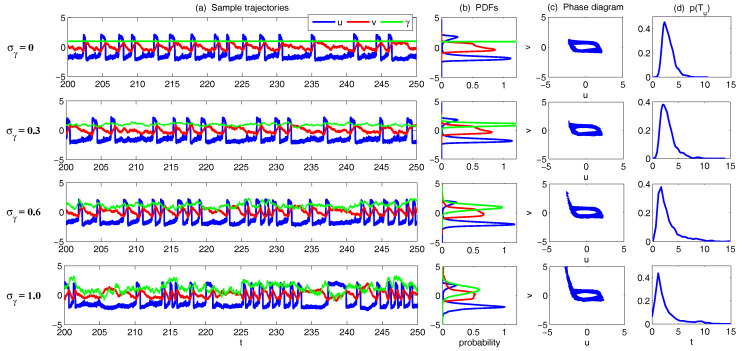
Simulations of the three-dimensional stochastically coupled FHN model with multiplicative noise (70). Different values of noise coefficient $\sigma_{\gamma }$ are used here. (**a**) sample trajectories of *u* (blue), *v* (red) and γ (green); (**b**) the associated PDFs; (**c**) phase diagram of (u,v); (**d**) distribution of the time interval *T* between successive oscillations in *u*.

Figure 12 shows the model simulations with different $\sigma_{\gamma }$. Note that, when $\sigma_{\gamma }=0$, the model (70) reduces to a two-dimensional model with $\gamma \equiv \hat{\gamma }$. From Figure 12, it is clear that *u* is always intermittent. On the other hand, *v* has only a small variation when γ is nearly a constant while more extreme events in *v* are observed when $\sigma_{\gamma }$ increases (see the PDF of *v*). Note that the extreme events in *v* are strongly correlated with the quiescent phase of *u* according to the phase diagram. These extreme events do not affect the regular ones that form a closed loop with the signal of *u*. With the increase of $\sigma_{\gamma }$, the periods of *u* becomes more irregular. This can be seen in (d), which shows the distribution of the time interval *T* between successive oscillations in *u*. With a large $\sigma_{\gamma }$, this distribution not only has a large variance but also shows a fat tail.

**Figure 13:**
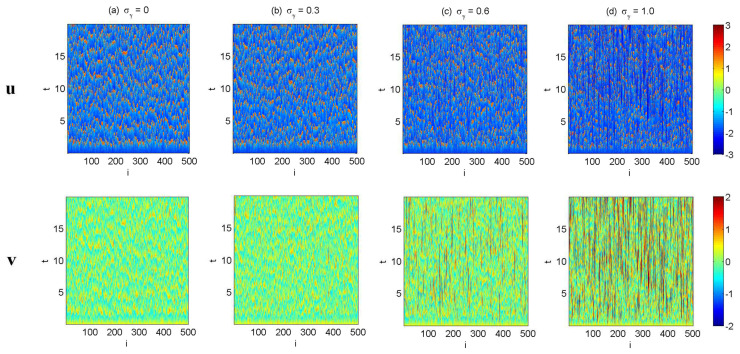
Simulations of the stochastically coupled FHN model with multiplicative noise system with spatial-extended structure (69). The same parameters as in (71) are taken and $d_{\gamma_{i}}=d_{\gamma }=1$ and ${\hat{\gamma }}_{i}=\hat{\gamma }=1$ for all *i*. Homogeneous initial conditions $u_{i}\left(0\right)=-2$ and $v_{i}\left(0\right)=0.5$ are adopted for all i=1,…,N. (**a**–**d**) show the simulation with different $\sigma_{\gamma }=0,0.3,0.6$ and 1.0, where $\sigma_{\gamma }$ is the noise coefficient at all the grid points, namely $\sigma_{\gamma_{i}}=\sigma_{\gamma }$ for all *i*.

Finally, Figure 13 shows the model simulation in the spatial-extended system (69), where n=500. The same parameters as in (71) are taken and $d_{\gamma_{i}}=d_{\gamma }=1$ and ${\hat{\gamma }}_{i}=\hat{\gamma }=1$ for all *i*. Homogeneous initial conditions $u_{i}\left(0\right)=-2$ and $v_{i}\left(0\right)=0.5$ are adopted for all i=1,…,N. The four rows show the simulation with different $\sigma_{\gamma }$, where $\sigma_{\gamma }$ is the noise coefficient at all the grid points, namely $\sigma_{\gamma_{i}}=\sigma_{\gamma }$ for all *i*. With the increase of $\sigma_{\gamma }$, the spatial structure of *u* becomes less coherent and more disorganized, which is consistent with the temporal structure as shown in Figure 12. In addition, more extreme events can be observed in the field of *v* due to the increase of the multiplicative noise.

#### 4.2.2. The Predator–Prey Models

The functioning of a prey–predator community can be described by a reaction–diffusion system. The standard deterministic predator–prey models have the following form [58]:

(72) $$\begin{array}{cc} \frac{du}{dt} & =D\nabla^{2}u+\frac{\alpha }{b}u\left(1-u\right)-\gamma \frac{u}{u+h}v,\\ \frac{dv}{dt} & =D\nabla^{2}v+\kappa \gamma \frac{u}{u+h}v-\mu v, \end{array}$$

where *u* and *v* represent predator and prey, respectively. Here, α,b,H, and γ are constants: α is the maximal growth rate of the prey, *b* is the carrying capacity for the prey population, and *H* is the half-saturation abundance of prey. Introducing dimensionless variables

$$\tilde{u}=u/b,\;\tilde{v}=v\gamma /\left(\alpha b\right),\;\tilde{t}=\alpha t,\;\tilde{r}=r{\left(\alpha /D\right)}^{1/2}$$

and, using the dimensionless parameters,

h=H/b,m=μ/α,k=κγ/α.

The non-dimensional system (by dropping the primes) becomes

(73) $$\begin{array}{cc} \frac{du}{dt} & =\nabla^{2}u+u\left(1-u\right)-\frac{u}{u+h}v,\\ \frac{dv}{dt} & =\nabla^{2}v+k\frac{u}{u+h}v-mv. \end{array}$$

Note that, in the predator–prey system, both *u* and *v* must be positive.

Clearly, the deterministic model is highly idealized and therefore stochastic noise is added into the system [144,145]. In order to keep the positive constraints for the variables *u* and *v*, multiplicative noise is added to the system. One natural choice of the noise is the following:

(74) $$\begin{array}{cc} \frac{du}{dt} & =\nabla^{2}u+u\left(1-u\right)-\frac{u}{u+h}v+f\left(u\right){\dot{W}}_{u},\\ \frac{dv}{dt} & =\nabla^{2}v+k\frac{u}{u+h}v-mv. \end{array}$$

Here, f(u) is a function of *u* and its value vanishes at u=0 to guarantee the positivity of the system. The most straightforward choice of f(u) is f(u)∝u, which leads to small noise when the signal of *u* is small and large noise when the amplitude of *u* is large. Another common choice of this multiplicative noise is $f\left(u\right)\propto u/\sqrt{1+u^{2}}$ such that the noise level remains nearly constant when *u* is larger than a certain threshold. Applying a spatial discretization to the diffusion terms, the stochastic coupled predator–prey in (74) belongs to the conditional Gaussian framework with $u_{I}=u$ and $u_{\mathrm{II}}=v$. Note that when f(u)≈0, the conditional Gaussian estimation (4) and (5) will become singular due to the term ${\left(f\left(u\right)f{\left(u\right)}^{*}\right)}^{-1}$. Nevertheless, the limit cycle in the dynamics will not allow the solution to be trapped at u=0. Assessing this issue using rigorous analysis is an interesting future work.

**Figure 14:**
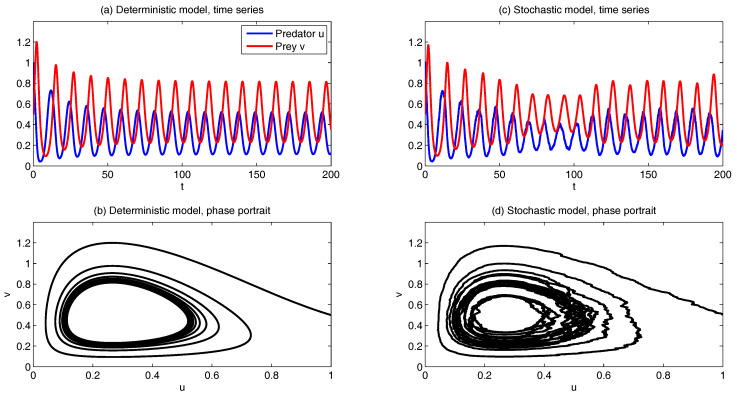
Sample trajectories and phase portraits under the simplest setup simulation of (74) without diffusion terms $\nabla^{2}u$ and $\nabla^{2}v$. The parameters are given in (75). (**a**,**b**) simulations without stochastic noise; (**c**,**d**) simulations with a multiplicative noise $f\left(u\right)=0.05u/\sqrt{1+u^{2}}$.

Figure 14 shows sample trajectories and phase portraits under the simplest setup simulation of (74) without diffusion terms $\nabla^{2}u$ and $\nabla^{2}v$. The parameters are given as follows:

(75) k=2,r=0.4,m=0.8,h=0.4.

Panels (a,b) show the simulations without stochastic noise while Panels (c,d) show the results with a multiplicative noise $f\left(u\right)=0.05u/\sqrt{1+u^{2}}$. The intermittent behavior is found in both *u* and *v*.

**Figure 15:**
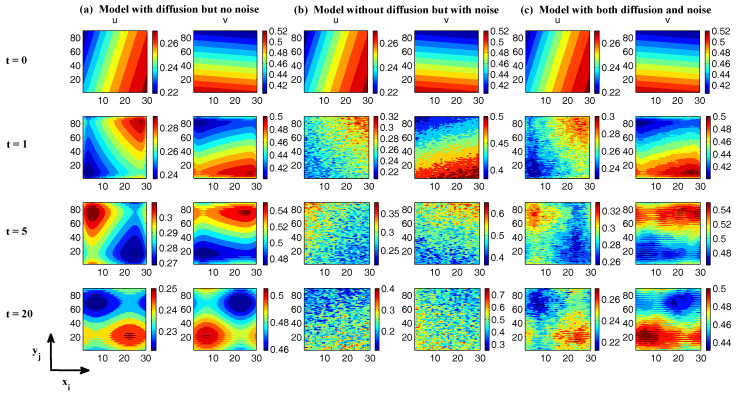
Snapshots at t=0,t=1,t=5 and t=20 of the spatial-extended system (74). Spatially periodic boundary conditions are used here. The parameters are given in (75). In all the three simulations in (**a–c**), the initial values for both *u* and *v* are the same. (**a**) model simulation of (74) with diffusion terms $\nabla^{2}u$ and $\nabla^{2}v$ but the noise coefficient f(u)=0; (**b**) model simulation of (74) without diffusion terms $\nabla^{2}u=\nabla^{2}v=0$. Therefore, the system is spatially decoupled. However, a stochastic noise $f\left(u\right)=0.05u/\sqrt{1+u^{2}}$ is added to the system; (**c**) simulations with both the diffusion terms $\nabla^{2}u$ and $\nabla^{2}v$ and the stochastic noise $f\left(u\right)=0.05u/\sqrt{1+u^{2}}$.

In Figure 15, we show the simulations of the spatial-extended system in (74), where we take 30 and 90 grid points in *x* and *y* directions, respectively. Spatially periodic boundary conditions are used here. In all three of the simulations in Columns (a–c), the initial values for both *u* and *v* are the same. Column (a) shows the model simulation of (74) with diffusion terms $\nabla^{2}u$ and $\nabla^{2}v$, but the noise coefficient is zero, namely f(u)=0. Therefore, the model is deterministic and spatial patterns can be seen in all the time instants. In Column (b), we ignore the diffusion terms $\nabla^{2}u=\nabla^{2}v=0$ and thus the system is spatially decoupled. Nevertheless, a stochastic noise $f\left(u\right)=0.05u/\sqrt{1+u^{2}}$ is added to the system. The consequence is that the initial correlated structure will be removed by the noise and, at t=5, the spatial structures are purely noisy. Finally, in Column (c), the simulations of the model with both the diffusion terms $\nabla^{2}u$, $\nabla^{2}v$ and the stochastic noise $f\left(u\right)=0.05u/\sqrt{1+u^{2}}$ are shown. Although after t=5, the initial structure completely disappears, the diffusion terms correlate the nearby grid points and spatial structures are clearly observed in these simulations. Due to the stochastic noise, the patterns are polluted and are more noisy than those shown in Column (a). In addition, the structure in *v* is more clear since the noise is directly imposed only on *u*.

#### 4.2.3. A Stochastically Coupled SIR Epidemic Model

The SIR model is one of the simplest compartmental models for epidemiology, and many models are derivations of this basic form [59,146]. The model consists of three compartments: “S” for the number susceptible, “I” for the number of infectious, and “R” for the number recovered (or immune). Each member of the population typically progresses from susceptible to infectious to recovered, namely

(76) susceptible⟶infectious⟶recovered.

This model is reasonably predictive for infectious diseases which are transmitted from human to human, and where recovery confers lasting resistance, such as measles, mumps and rubella.

The classical SIR model is as follows:

(77) $$\begin{array}{cc} dS & =(-\beta SI-\mu_{1}S+b)dt,\\ dI & =(\beta SI-\mu_{2}I-\alpha I)dt,\\ dR & =(\alpha I-\mu_{3}R)dt, \end{array}$$

where the total population size has been normalized to one and the influx of the susceptible comes from a constant recruitment rate *b*. The death rate for the S,I and *R* class is, respectively, given by $\mu_{1},\mu_{2}$ and $\mu_{3}$. Biologically, it is natural to assume that $\mu_{1}<\min\left\{\mu_{2},\mu_{3}\right\}$. The standard incidence of disease is denoted by βSI, where β is the constant effective contact rate, which is the average number of contacts of the infectious per unit time. The recovery rate of the infectious is denoted by α such that 1/α is the mean time of infection.

When the distribution of the distinct classes is in different spatial locations, the diffusion terms should be taken into consideration and random noise can also be added. Thus, an extended version of the above SIR system (77) can be described as the following [147,148,149]:

(78) $$\begin{array}{cc} dS & =\left(\nabla^{2}S-\beta SI-\mu_{1}S+b\right)dt+\sigma \left(S\right)dW_{S},\\ dI & =(\nabla^{2}I+\beta SI-\mu_{2}I-\alpha I)dt,\\ dR & =(\nabla^{2}R+\alpha I-\mu_{3}R)dt, \end{array}$$

where the noise is multiplicative in order to guarantee the positivity of the three variables. Clearly, the SIR model in (78) is a conditional Gaussian system with $u_{I}=S$ and $u_{\mathrm{II}}={\left(I,R\right)}^{T}$. It can be used to estimate and predict the number of both infectious and recovered given those susceptible. Note that the SIS model (the model with only S and I variables) [150] is a special case of SIR model and it naturally belongs to the conditional Gaussian framework.

#### 4.2.4. A Nutrient-Limited Model for Avascular Cancer Growth

Here, we present a nutrient-limited model for avascular cancer growth [60], where the cell actions (division, migration, and death) are locally controlled by the nutrient concentration field. Consider a single nutrient field described by the diffusion equation:

(79) $$dN=(D_{N}\nabla^{2}N-\gamma N\sigma_{n}-\lambda \gamma N\sigma_{c})dt$$

in which γ and λ are the nutrient consumption rates of normal and cancer cells, respectively. The domain is the tissue which is represented by a square lattice of size (L+1)×(L+1) and lattice constant Δ. On the other hand, the growth factor (GF) concentration obeys the diffusion equation

(80) $$dG=(D_{G}\nabla^{2}G-k^{2}G+\Gamma \sigma_{c}N\left(G_{M}-G\right))dt,$$

which includes the natural degradation of GFs, also imposing a characteristic length ∼1/k for GFs diffusion, and a production term increasing linearly with the local nutrient concentration up to a saturation value $G_{M}$. Therefore, we are assuming that the release of GFs involves complex metabolic processes supported by nutrient consumption. The boundary conditions satisfied by the GF concentration field is $G(\vec{x},t)=0$ at a large distance (d>2/k) from the tumor border. Define the non-dimensional variables:

$$\begin{array}{c} t^{\prime }=\frac{D_{N}t}{\Delta^{2}},\;{\vec{x}}^{\prime }=\frac{{\vec{x}}^{\prime }}{\Delta },\;N^{\prime }=\frac{N}{K_{0}},\;G^{\prime }=\frac{G}{G_{M}},\\ \alpha ={\left(\frac{\gamma \Delta^{2}}{D_{N}}\right)}^{1/2},\;k^{\prime }=k{\left(\frac{\Delta^{2}}{D_{N}}\right)}^{1/2},\;\Gamma^{\prime }=\frac{\Gamma \Delta^{2}}{D_{N}},\;D=\frac{D_{G}}{D_{N}}, \end{array}$$

with these new variables (dropping the primes) and stochastic noise, the system (79) and (80) becomes

(81) $$\begin{array}{cc} dN & =\left(\nabla^{2}N-\alpha^{2}N\sigma_{n}-\lambda \alpha^{2}N\sigma_{c}\right)dt+\sigma_{N}dW_{N},\\ dG & =\left(D\nabla^{2}G-k^{2}G+\Gamma \sigma_{c}N\left(1-G\right)\right)dt+\sigma_{G}dW_{G}. \end{array}$$

The coupled system (81) is a conditional Gaussian system with the observations given by the GF concentration $u_{I}=G$ and the state estimation for the nutrient field $u_{\mathrm{II}}=N$.

### 4.3. Large-Scale Dynamical Models in Turbulence, Fluids and Geophysical Flows

#### 4.3.1. The MJO Stochastic Skeleton Model

The Madden–Julian oscillation (MJO) is the dominant mode of variability in the tropical atmosphere on intraseasonal time scales and planetary spatial scales [151,152,153]. It affects both tropical and extratropical weather and climate. It can also possibly trigger and modify the El Niño-Southern Oscillation [154,155,156]. Understanding and predicting the MJO is a central problem in contemporary meteorology with large societal impacts.

In [51], a stochastic skeleton model was developed that recovers robustly the most fundamental MJO features: (1) a slow eastward speed of roughly 5 m/s; (2) a peculiar dispersion relation with dω/dk≈0; (3) a horizontal quadrupole vortex structure; (4) the intermittent generation of MJO events; and (5) the organization of MJO events into wave trains with growth and demise, as seen in nature. In fact, the first three features are already covered by the deterministic version of the skeleton model [157,158]. The last two ones are significantly captured by the stochastic version [51]. Using a method based on theoretical waves structures, the MJO skeleton from the model is identified in the observational data [159]. In addition, the stochastic skeleton model is capable of reproducing observed MJO statistics such as the average duration of MJO events and the overall MJO activity [160].

The MJO stochastic skeleton model is given as follows [51]:

(82) $$\begin{array}{cc} u_{t}-yv-\theta_{x} & =0,\\ yu-\theta_{y} & =0,\\ \theta_{t}-u_{x}-v_{y} & =\bar{H}a-s^{\theta },\\ q_{t}+\bar{Q}\left(u_{x}+v_{y}\right) & =-\bar{H}a+s^{q},\\ a_{t} & =\mathrm{stochastic}\;\mathrm{birth-death}\;\mathrm{process}, \end{array}$$

and the expectation of the convective activity *a* satisfies

(83) $$a_{t}=\Gamma qa,$$

where $\cdot_{t}$, $\cdot_{x}$ and $\cdot_{y}$ denote the derivatives with respect to time *t* zonal (east-west) coordinate *x* and meridional (north-south) coordinate *y*. In (82), *u*, *v* and θ are the zonal velocity, meridional velocity and the potential temperature, respectively, and *a* is the envelope of convective activity. The fourth equation describes the evolution of low-level moisture *q*. All variables are anomalies from a radiative-convective equilibrium, except *a*. The skeleton model contains a minimal number of parameters: $\bar{Q}$ is the background vertical moisture gradient, Γ is a proportionality constant. $\bar{H}$ is irrelevant to the dynamics but allows us to define a cooling/drying rate $\bar{H}a$ for the system in dimensional units. $s^{\theta }$ and $s^{q}$ are external sources of cooling and moistening that need to be prescribed in the system. Notably, the planetary envelope a≥0 in particular is a collective representation of the convection/wave activity occurring at sub-planetary scale, the details of which are unresolved. A key part of the q−a interaction is the assumption that *q* influences the tendency of a $a_{t}=\Gamma qa$, where Γ>0 is a constant.

Next, the system (82) is projected onto the first Hermite function in the meridional direction such that $a\left(x,y,t\right)=A\left(x,t\right)\phi_{0},q=Q\phi_{0},s^{q}=S^{q}\phi_{0},s^{\theta }=S^{\theta }\phi_{0}$, where $\phi_{0}\left(y\right)=\sqrt{2}{\left(4\pi \right)}^{-1/4}\exp\left(-y^{2}/2\right)$. Such a meridional heating structure is known to excite only Kelvin waves and the first symmetric equatorial Rossby waves [11,161]. The resulting meridionally truncated equations are

(84) $$\begin{array}{cc} K_{t}+K_{x} & =(S^{\theta }-\bar{H}A)/2, \end{array}$$

(85) $$\begin{array}{cc} R_{t}-R_{x}/3 & =(S^{\theta }-\bar{H}A)/3, \end{array}$$

(86) $$\begin{array}{cc} Q_{t}+\bar{Q}\left(K_{x}-R_{x}/3\right) & =\left(\bar{H}A-S^{q}\right)\left(\bar{Q}/6-1\right), \end{array}$$

(87) $$\begin{array}{cc} A_{t} & =\mathrm{stochastic}\;\mathrm{birth-death}\;\mathrm{process}. \end{array}$$

The expectation of the convective activity *A* satisfies

(88) $$A_{t}=\Gamma QA.$$

**Figure 16:**
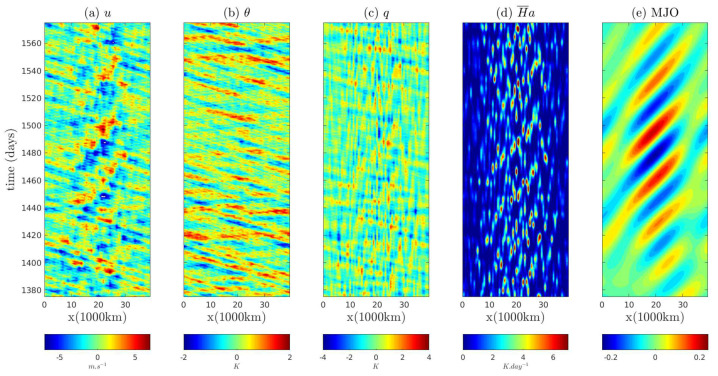
(**a**) Velocity field *u*; (**b**) temperature θ; (**c**) moisture *q*; (**d**) convective activity $\bar{H}a$ and (**e**) the MJO patterns in the MJO stochastic skeleton model (84)–(87). The *x*-axis is the zonal region that extends over the entire equator. The *y*-axis is time.

Figure 16 shows one model simulation of different fields (Panels (a–d)) as well as the MJO patterns (Panel (e)) with realistic warm pool background heating and moisture sources [51]. The MJO patterns are calculated by the combination of different fields using eigen-decomposition [51]. All the features mentioned at the beginning of this subsection can be clearly seen in Panel (e).

To provide intuition of forming a conditional Gaussian system from the original MJO skeleton model, we start with a simplified version of the full spatially extended system in (84)–(87) and (88), namely the stochastic skeleton single-column model,

(89) $$\begin{array}{cc} \frac{dK}{dt} & =(S^{\theta }-\bar{H}A)/2, \end{array}$$

(90) $$\begin{array}{cc} \frac{dR}{dt} & =(S^{\theta }-\bar{H}A)/3, \end{array}$$

(91) $$\begin{array}{cc} \frac{dQ}{dt} & =\left(\bar{H}A-S^{q}\right)\left(\bar{Q}/6-1\right), \end{array}$$

(92) $$\begin{array}{cc} \frac{dA}{dt} & =\mathrm{stochastic}\;\mathrm{birth-death}\;\mathrm{process}, \end{array}$$

where the expectation of the convective activity *A* satisfies

(93) $$\frac{dA}{dt}=\Gamma QA.$$

In the single-column model, the spatial derivative disappears and the coupled system becomes a four-dimensional stochastic ODEs. In the original stochastic skeleton model [51], the process of the convective activity *A* is driven by a stochastic birth-death process, which provides a clear physical interpretation of the evolution of the convective activity. Note that in the continuous limit of the stochastic birth-death process (the small jump between the two nearby states Δa→0), (92) converges to a continuous SDE

(94) $$dA=\Gamma QAdt+\sqrt{\lambda A}dW^{A},$$

where $W_{A}$ is white noise. See [162] for more details. It is clear that (94) involves a multiplicative noise that guarantees the non-negativity of *A*. In addition, the expectation of the convective activity *A* converges to (93). Replacing (92) by (94), we arrive at the following system:

(95) $$\begin{array}{cc} dA & =\Gamma QAdt+\sqrt{\lambda A}dW^{A}, \end{array}$$

(96) $$\begin{array}{cc} dK & =\frac{1}{2}\left(S^{\theta }-\bar{H}A\right)dt, \end{array}$$

(97) $$\begin{array}{cc} dR & =\frac{1}{3}\left(S^{\theta }-\bar{H}A\right)dt, \end{array}$$

(98) $$\begin{array}{cc} dQ & =\left(1-\frac{\bar{Q}}{6}\right)\left(S^{q}-\bar{H}A\right)dt, \end{array}$$

which is a conditional Gaussian system with $u_{I}=A$ and $u_{\mathrm{II}}=\left(K,R,Q\right)$. Note that, in the content of data assimilation and prediction, judicious model errors [20,28] are often added to the system. These judicious model errors include extra damping and stochastic noise that balance the intrinsic model error and the observational error as well as improve the model capability in capturing the uncertainty. With these judicious model errors, the system (95)–(98) can be modified as

(99) $$\begin{array}{cc} dA & =\Gamma QAdt+\sqrt{\lambda A}dW^{A}, \end{array}$$

(100) $$\begin{array}{cc} dK & =\left(-{\bar{d}}^{K}K+\frac{1}{2}\left(S^{\theta }-\bar{H}A\right)\right)dt+\sigma^{K}dW^{K}, \end{array}$$

(101) $$\begin{array}{cc} dR & =\left(-{\bar{d}}^{R}R+\frac{1}{3}\left(S^{\theta }-\bar{H}A\right)\right)dt+\sigma^{R}dW^{R}, \end{array}$$

(102) $$\begin{array}{cc} dQ & =\left(-{\bar{d}}^{Q}Q+\left(1-\frac{\bar{Q}}{6}\right)\left(S^{q}-\bar{H}A\right)\right)dt+\sigma^{Q}dW^{Q}. \end{array}$$

Now let us consider the spatially extended system (84)–(87). Making use of the finite Fourier decomposition of the state variables

$$F\left(x_{j},t\right)=\sum_{-M+1\leq k\leq M}{\hat{F}}_{k}\left(t\right)e^{2\pi ikx_{j}/L}=\sum_{-M+1\leq k\leq M}{\hat{F}}_{k}\left(t\right)e^{il_{k}x_{j}},\;F=\left\{A,K,R,Q\right\}$$

and plugging them into the truncated system (84)–(87), we reach the following stochastic system:

(103) $$\begin{array}{cc} \frac{d{\hat{A}}_{k}}{dt} & =\Gamma \sum_{-M+1\leq s\leq M}{\hat{Q}}_{s}{\hat{A}}_{k-s}+\sqrt{\lambda_{k}{\hat{A}}_{k}}{\dot{W}}_{k}^{A},\\ \frac{d{\hat{K}}_{k}}{dt} & =\left(-il_{k}-{\bar{d}}_{k}^{K}\right){\hat{K}}_{k}+\frac{1}{2}\left({\hat{S}}_{k}^{\theta }-\bar{H}{\hat{A}}_{k}\right)+\sigma_{k}^{K}{\dot{W}}_{k}^{K},\\ \frac{d{\hat{R}}_{k}}{dt} & =\left(il_{k}/3-{\bar{d}}_{k}^{R}\right){\hat{R}}_{k}+\frac{1}{3}\left({\hat{S}}_{k}^{\theta }-\bar{H}{\hat{A}}_{k}\right)+\sigma_{k}^{R}{\dot{W}}_{k}^{R},\\ \frac{d{\hat{Q}}_{k}}{dt} & =-{\bar{d}}_{k}^{Q}{\hat{Q}}_{k}-\bar{Q}il_{k}\left({\hat{K}}_{k}-\frac{{\hat{R}}_{k}}{3}\right)+\left(1-\frac{\bar{Q}}{6}\right)\left({\hat{S}}_{k}^{q}-\bar{H}{\hat{A}}_{k}\right)+\sigma_{k}^{Q}{\dot{W}}_{k}^{Q},\\ & -M+1\leq k\leq M, \end{array}$$

where judicious model errors with extra damping and stochastic noise are added in these equations. In addition to improving the skill in data assimilation and prediction, these extra damping and stochastic noise also compensate the small scale information that has been truncated in the finite Fourier decomposition.

It is clear that the nonlinear model (103) is a conditional Gaussian system with

$$u_{I}=\left\{{\hat{A}}_{k}\right\},\;\mathrm{and}\;u_{\mathrm{II}}=\left(\left\{{\hat{K}}_{k}\right\},\left\{{\hat{R}}_{k}\right\},\left\{{\hat{Q}}_{k}\right\}\right).$$

Linking (103) to the original system (82), *u*, *v*, θ and *q* belong to $u_{\mathrm{II}}$ and the wave activity *a* is the observed variable $u_{I}$. Note that there are several further studies of the stochastic skeleton model. By involving additional off equatorial components of convective heating and adding a simple seasonal cycle in a warm pool background [163], the skeleton model succeeds in reproducing the meridionally asymmetric intraseasonal events and a seasonal modulation of intraseasonal variability. Another extended version of the skeleton model involves the refined vertical structure of the MJO in nature [164]. All of these modified versions belong to the conditional Gaussian framework. Note that a rigorous proof has shown that the stochastic skeleton model has the geometric ergodicity property [165].

#### 4.3.2. A Coupled El Niño Model Capturing Observed El Niño Diversity

The El Niño Southern Oscillation (ENSO) has significant impact on global climate and seasonal prediction [166,167,168]. It is the most prominent year-to-year climate variation on earth, with dramatic ecological and social impacts. The traditional El Niño consists of alternating periods of anomalously warm El Niño conditions and cold La Niña conditions in the equatorial Pacific every two to seven years, with considerable irregularity in amplitude, duration, temporal evolution and spatial structure of these events. In recent decades, a different type of El Niño has been frequently observed [169,170,171,172], which is called the central Pacific (CP) El Niño. Different from the traditional El Niño where warm sea surface temperature (SST) occurs in the eastern Pacific, the CP El Niño is characterized by warm SST anomalies confined to the central Pacific, flanked by colder waters to both east and west. Understanding and predicting El Niño diversity has significant scientific and social impacts [173,174].

In [52], a simple modeling framework has been developed that automatically captures the statistical diversity of ENSO. The starting model is a deterministic, linear and stable model that includes the coupled atmosphere, ocean and SST [175]. Then, key factors are added to the system in a systematic way. First, a stochastic parameterization of the wind bursts including both westerly and easterly winds is coupled to the starting model that succeeds in capturing both the eastern Pacific El Niño events and the statistical features in the eastern Pacific [175]. Secondly, a simple nonlinear zonal advection with no ad hoc parameterization of the background SST gradient and a mean easterly trade wind anomaly representing the multidecadal acceleration of the trade wind are both incorporated into the coupled model that enable anomalous warm SST in the central Pacific [176]. Then, a three-state stochastic Markov jump process is utilized to drive the wind burst activity that depends on the strength of the western Pacific warm pool in a simple and effective fashion [52]. It allows the coupled model to simulate the quasi-regular moderate traditional eastern Pacific El Niño, the super El Niño, the CP El Niño as well as the La Niña with realistic features [177]. In particular, the model succeeds in capturing and predicting different super El Niño events [178], including both the directly formed (similar to 1997–1998) and delayed events (similar to 2014–2016). An improved version of the model is also able to recover the season synchronization [179]. The coupled model is as follows:

*Atmosphere*
  (104) $$\begin{array}{cc} -yv-\partial_{x}\theta & =0,\\ yu-\partial_{y}\theta & =0,\\ -(\partial_{x}u+\partial_{y}v) & =E_{q}/\left(1-\bar{Q}\right). \end{array}$$
*Ocean*
  (105) $$\begin{array}{cc} \partial_{\tau }U-c_{1}YV+c_{1}\partial_{x}H & =c_{1}\tau_{x},\\ YU+\partial_{Y}H & =0,\\ \partial_{\tau }H+c_{1}\left(\partial_{x}U+\partial_{Y}V\right) & =0. \end{array}$$
*SST*
  (106) $$\partial_{\tau }T+\mu \partial_{x}\left(UT\right)=-c_{1}\zeta E_{q}+c_{1}\eta H,$$
Coupling:
  (107) $$E_{q}=\alpha_{q}T,\;\tau_{x}=\gamma \left(u+u_{p}\right).$$

Here, *x* is zonal direction and τ is interannual time, while *y* and *Y* are meridional direction in the atmosphere and ocean, respectively. The u,v are zonal and meridional winds, θ is potential temperature, U,V are zonal and meridional currents, *H* is thermocline depth, *T* is SST, $E_{q}$ is latent heating, and $\tau_{x}$ is zonal wind stress. All variables are anomalies from an equilibrium state, and are non-dimensional. The coefficient $c_{1}$ is a non-dimensional ratio of time scales, which is of order O(1). The term $u_{p}$ describes stochastic wind burst activity. The atmosphere extends over the entire equatorial belt $0\leq x\leq L_{A}$ with periodic boundary conditions, while the Pacific ocean extends over $0\leq x\leq L_{O}$ with reflection boundary conditions for the ocean model and zero normal derivative at the boundaries for the SST model. The wind bursts and easterly mean trade wind are parameterized as

(108) $$\begin{array}{cc} u_{p} & =a_{p}\left(\tau \right)s_{p}\left(x\right)\phi_{0}\left(y\right),\\ \frac{da_{p}}{d\tau } & =-d_{p}\left(a_{p}-{\hat{a}}_{p}\right)+\sigma_{p}\left(T_{W}\right)\dot{W}\left(\tau \right), \end{array}$$

where the noise $\sigma_{p}\left(T_{W}\right)$ is a state-dependent noise coefficient given by a three-state Markov jump process. In addition, $s_{p}\left(x\right)$ and $\phi_{0}\left(y\right)$ are the prescribed zonal and meridional bases.

**Figure 17:**
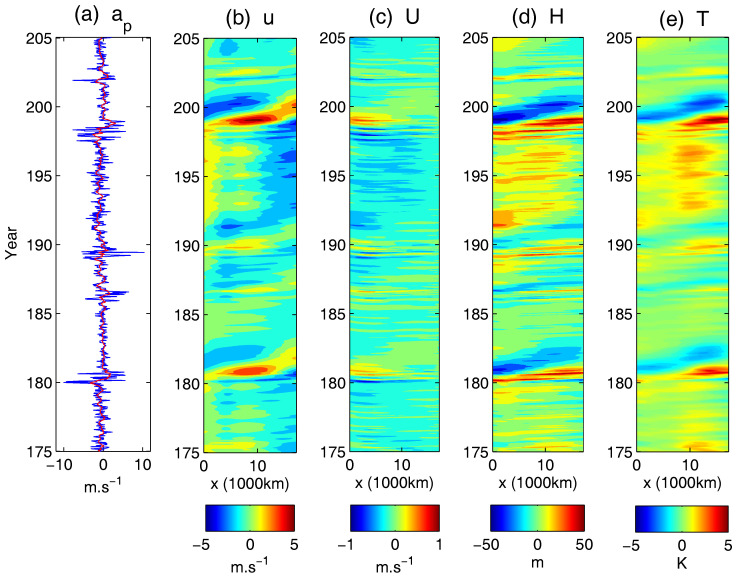
Model simulations of the coupled ENSO model (104)–(108). The *x*-axis is the zonal region of the equatorial Pacific. The *y*-axis is time. (**a**) time series of the stochastic wind burst amplitude $a_{p}$ (blue) and its 90-day running mean; (**b**–**e**) atmosphere wind *u*, ocean current *U*, thermocline depth *H* and SST *T*.

Applying the meridional truncation to the coupled system (104)–(108), the coupled model reduces to a set of PDEs that depends only on time *t* and zonal variables *x*. These equations describe the zonal propagation of atmosphere and ocean Kelvin and Rossby waves [52]. Figure 17 shows a model simulation. The El Niño diversity is clearly captured, where a traditional eastern Pacific ENSO cycle (t=181–182), a series of CP El Niño (t=192–197) and a super El Niño (t=199) are all observed. Similar to the MJO skeleton model in Section 4.3.1, a suitable set of zonal bases are chosen and the coupled ENSO model becomes a large dimension of coupled ODE system. By adding judicious model errors with extra damping and stochastic forcing (as in the MJO skeleton model), the resulting system belongs to the conditional Gaussian framework, where $u_{I}$ includes SST and $u_{\mathrm{II}}$ contains all the variables in atmosphere and ocean.

#### 4.3.3. The Boussinesq Equation

The Boussinesq approximation [11] is a way to solve nonisothermal flow without having to solve for the full compressible formulation of the Navier–Stokes equations. It assumes that variations in density have no effect on the flow field, except that they give rise to buoyancy forces. The Boussinesq equation is derived when the Boussinesq approximation is applied to the full Navier–Stokes equation:

(109) $$\begin{array}{cc} \nabla \cdot u & =0,\\ \frac{\partial u}{\partial t}+u\cdot \nabla u & =-\frac{1}{\rho_{0}}\nabla p+\nu \nabla^{2}u-g\alpha T+F_{u},\\ \frac{\partial T}{\partial t}+u\cdot \nabla T & =\kappa \nabla^{2}T+F_{T}, \end{array}$$

where *T* is the temperature, u is the velocity fields, $\rho_{0}$ is the reference density, *p* is the pressure, *g* is the acceleration due to gravity, $F_{u}$ and $F_{T}$ are the external forcing and κ is the diffusion coefficient. Note that the forcing terms $F_{u}$ and $F_{T}$ can involve both deterministic and stochastic forcing. The three equations in (109) are the continuity equation, the momentum equation and the thermal equation, respectively. The Boussinesq equation has a wide application, including modeling the Rayleigh–Bénard convection [53,54] and describing strongly stratified flows as in geophysics [55].

Here for simplicity, let us assume the boundary conditions are periodic. The framework below can be easily extended to the Boussinesq system with more general boundary conditions. Similar to the general procedure as described in Section 4.3.1, applying a finite Fourier truncation to (109) and adding judicious model errors with extra damping and noise if needed, the resulting system consists of a large dimension of stochastic ODEs. Here, the continuity equation with divergence free condition provides the eigen-directions of the each Fourier mode associated with the velocity field [11], namely,

$$u\left(x,t\right)=\sum_{0<|k|\leq K}\hat{u}\left(t\right)e^{ikx}r_{k},$$

where $r_{k}$ is the eigen-direction associated with the wavenumber k. Note that the resulting system remains highly nonlinear due to the quadratic coupling between different Fourier modes in the advection u·∇u and u·∇T. The straightforward calculation shows that the system belongs to the conditional Gaussian framework with $u_{I}=u$ and $u_{\mathrm{II}}=T$, where the right-hand side should be understood as the Fourier modes. Such a setup allows the state estimation of the temperature *T* given the noisy velocity field u using the closed form (4) and (5) in the conditional Gaussian framework.

In many applications, it is easier to observe the temperature *T* while the velocity field u is required to be estimated. However, if we choose $u_{I}=T$ and $u_{\mathrm{II}}=u$, then (109) is not a conditional Gaussian system. To satisfy the requirement of the conditional Gaussian framework, the equation of the velocity field has to be linearized. Nevertheless, in the situation with large Pradlt number [54,180,181], which occurs quite often in applications (such as Rayleigh–Bénard convection), the nonlinear term u·∇u can be dropped and the resulting system becomes

(110) $$\begin{array}{cc} \nabla \cdot u & =0,\\ \frac{\partial u}{\partial t} & =-\frac{1}{\rho_{0}}\nabla p+\nu \nabla^{2}u-g\alpha T+F_{u},\\ \frac{\partial T}{\partial t}+u\cdot \nabla T & =\kappa \nabla^{2}T+F_{T}. \end{array}$$

It is important to note that the coupled system remains nonlinear due to the nonlinear coupling u·∇T. One interesting issue is to understand the model error by dropping the term u·∇u in filtering the resulting conditional Gaussian systems.

#### 4.3.4. Darcy–Brinkman–Oberbeck–Boussinesq System—Convection Phenomena in Porous Media

The problem considered in this subsection is the convection phenomena in porous media which is relevant to a variety of science and engineering problems ranging from geothermal energy transport to fiberglass insulation. Consider a Rayleigh–Bénard like problem: convection in a porous media region, Ω, bounded by two parallel planes saturated with fluids. The bottom plate is kept at temperature $T_{2}$ and the top plate is kept at temperature $T_{1}$ with $T_{2}>T_{1}$. One of the famous models is the following Darcy–Brinkman–Oberbeck–Boussinesq system in the non-dimensional form [56]:

(111) $$\begin{array}{cc} \nabla \cdot v & =0,\\ \gamma_{a}\left(\frac{\partial v}{\partial t}+\left(v\cdot \nabla \right)v\right)+v-\tilde{Da}\Delta v+\nabla p & =Ra_{D}kT+F_{v},\\ \frac{\partial T}{\partial t}+v\cdot \nabla T & =\Delta T+F_{T}, \end{array}$$

where k is the unit normal vector directed upward, v is the non-dimensional seepage velocity, *p* is the non-dimensional kinematic pressure, *T* is the non-dimensional temperature. As in (109), $F_{v}$ and $F_{T}$ here are external forcing, which involve both deterministic and stochastic parts. The parameters in the system are given by the Prandtl–Darcy number, $\gamma_{a}^{-1}$ , which is defined as $\gamma_{a}^{-1}=\left(\nu h^{2}\right)/\left(\kappa K\right)$ where ν is the kinematic viscosity of the fluid, *h* is the distance between the top and bottom plates, κ is the thermal diffusivity and *K* is the permeability of the fluid; the Brinkman–Darcy number, $\tilde{Da}$, which is given by $\tilde{Da}=\left(\mu_{eff}K\right)/\left(\nu h^{2}\right)$, where $\nu_{eff}$ is the effective kinematic viscosity of the porous media; and the Rayleigh–Darcy number, $Ra_{D}$, which takes the form $Ra_{D}=\left(g\gamma \left(T_{2}-T_{1}\right)Kh\right)/\left(\nu \kappa \right)$, where *g* is the gravitational acceleration constant, α is the thermal expansion coefficient. The parameter $\gamma_{a}$ is also called the non-dimensional acceleration coefficient.

As in the Boussinesq equation, by applying finite Fourier expansion and adding extra damping and noise to the Darcy–Brinkman–Oberbeck–Boussinesq model (111), a conditional Gaussian system is reached with $u_{I}=v$ and $u_{\mathrm{II}}=T$. Again, the right-hand side should be understood as Fourier coefficients.

On the other hand, applying linearization and dropping one of the nonlinear terms (v·∇)v in (111), the temperature *T* can be viewed as the observed variable to filter the seepage velocity v,

$$u_{I}=T\;\mathrm{and}\;u_{\mathrm{II}}=v.$$

The resulting system is given by

(112) $$\begin{array}{cc} \nabla \cdot v & =0,\\ \gamma_{a}\frac{\partial v}{\partial t}+v-\tilde{Da}\Delta v+\nabla p & =Ra_{D}kT+F_{v},\\ \frac{\partial T}{\partial t}+v\cdot \nabla T & =\Delta T+F_{T}. \end{array}$$

#### 4.3.5. The Rotating Shallow Water Equations

The rotating shallow water equation is an appropriate approximation for atmospheric and oceanic motions in the midlatitudes with relatively large length and time scales [11]. In such regimes, the effects of the earth’s rotation are important when the fluid motions evolve on a time scale that is comparable or longer than the time scale of rotation. Note that the rotation shallow water equations do not include the effects of density stratification as observed in many other phenomena.

The rotating shallow water equation is as follows:

(113) $$\begin{array}{cc} \frac{\partial u}{\partial t}+u\cdot \nabla u+fu^{⊥}+g\nabla h & =F_{u},\\ \frac{\partial h}{\partial t}+u\cdot \nabla h+\left(H+h\right)\nabla \cdot u & =F_{h}, \end{array}$$

where u is the two-dimensional velocity field in the (x,y)-plane, *h* is the geophysical height. On the right-hand side of (113), $F_{u}$ and $F_{h}$ are forcing terms that include both deterministic and stochastic forcing. As discussed in the previous subsections, choosing $u_{I}=u$ and $u_{\mathrm{II}}=h$ and applying the procedure in Section 4.3.1 with finite Fourier expansion and judicious model error, a conditional Gaussian system is formed.

Alternatively, in some applications, the height variable *h* can be measured and the velocity field u is required to be filtered or estimated. Thus, it is natural to set

$$u_{I}=h\;\mathrm{and}\;u_{\mathrm{II}}=u$$

and then making use of data assimilation to estimate the velocity field. To allow such a problem to fit into conditional Gaussian framework, one of the nonlinear terms u·∇u is dropped and the resulting system is given by

(114) $$\begin{array}{cc} \frac{\partial h}{\partial t}+u\cdot \nabla h+\left(H+h\right)\nabla \cdot u & =F_{h},\\ \frac{\partial u}{\partial t}+fu^{⊥}+g\nabla h & =F_{u}. \end{array}$$

The equations in (114) are then put into the framework in Section 4.3.1 that forms a conditional Gaussian system.

As a side remark, linearizing the shallow water Equation (113) and writing into characteristic forms of the resulting equations provide two types of modes:

1. Geostrophically balanced (GB) modes: $\omega_{\vec{k},B}=0$; incompressible.
2. Gravity modes: $\omega_{\vec{k},\pm }=\pm \epsilon^{-1}\sqrt{\delta {\left|\vec{k}\right|}^{2}+1}$; compressible.

Let us denote ${\hat{v}}_{\vec{k},B}$ and ${\hat{v}}_{\vec{k},\pm }$ as the random coefficients of GB and gravity modes for each Fourier wavenumber, respectively. Following the idea in [20], a coupled system that describes the interactions between the GB and gravity modes are given by

(115) $$\begin{array}{cc} d{\hat{v}}_{\vec{k},B} & =\left(-d_{B}{\hat{v}}_{\vec{k},B}+f_{\vec{k},B}\left(t\right)\right)dt+\sigma_{\vec{k},B}dW_{\vec{k},B}\left(t\right),\\ d{\hat{v}}_{\vec{k},\pm } & =\left(\;\left(-d_{g}+i\omega_{\vec{k},\pm }+i{\hat{v}}_{\vec{k},B}\right){\hat{v}}_{\vec{k},\pm }+f_{\vec{k},\pm }\left(t\right)\;\right)dt+\sigma_{\vec{k},\pm }dW_{\vec{k},\pm }\left(t\right). \end{array}$$

Collecting the equations for different Fourier wavenumbers forms a coupled systems for the GB and gravity modes. Note that the GB modes are in slow time scale while the gravity modes occur in a fast time scale. Denote

$$u_{I}=\left\{{\hat{v}}_{\vec{k},B}\right\}\;\mathrm{and}\;u_{\mathrm{II}}=\left\{{\hat{v}}_{\vec{k},\pm }\right\}.$$

The coupled system (115) including all the Fourier wavenumbers *k* belong to the conditional Gaussian framework.

### 4.4. Coupled Observation-Filtering Systems for Filtering Turbulent Ocean Flows Using Lagrangian Tracers

Lagrangian tracers are drifters and floaters that collect real-time information of fluid flows [182]. Filtering (or data assimilation) of the turbulent ocean flows using Lagrangian tracers provides a more accurate state estimation and reduces the uncertainty [183,184,185]. In [61,62,63], a first rigorous mathematical framework was developed and it was applied to study (1) the information barrier in filtering [61]; (2) the filtering skill of multiscale turbulent flows [62] and (3) the model error in various cheap and practical reduced filters with both linear and strongly nonlinear underlying flows [63].

Consider a *d* dimensional random flow modeled by a finite number of Fourier modes with random amplitudes in periodic domain ${\left(0,2\pi \right]}^{d}$,

$$\vec{v}\left(\vec{x},t\right)=\sum_{\vec{k}\in K}{\hat{v}}_{\vec{k}}\left(t\right)\cdot e^{i\vec{k}\cdot \vec{x}}\cdot {\vec{r}}_{\vec{k}}.$$

Each ${\hat{v}}_{k}\left(t\right)$ follows an OU process,

(116) $$d{\hat{v}}_{\vec{k}}\left(t\right)=-d_{\vec{k}}{\hat{v}}_{\vec{k}}\left(t\right)dt+f_{\vec{k}}\left(t\right)dt+\sigma_{\vec{k}}dW_{\vec{k}}^{v}\left(t\right).$$

The observations are given by the trajectories of *L* noisy Lagrangian tracers,

$$\begin{array}{cc} d{\vec{x}}_{l}\left(t\right) & =\vec{v}\left({\vec{x}}_{l}\left(t\right),t\right)dt+\sigma_{x}dW_{l}^{x}\left(t\right)\\ & =\sum_{\vec{k}\in K}{\hat{v}}_{\vec{k}}\left(t\right)\cdot e^{i\vec{k}\cdot {\vec{x}}_{l}\left(t\right)}\cdot {\vec{r}}_{\vec{k}}dt+\sigma_{x}dW_{l}^{x}\left(t\right),\;l=1,\ldots ,L, \end{array}$$

which is highly nonlinear due to the appearance of the ${\vec{x}}_{l}\left(t\right)$ in the exponential term.

Collecting all the Fourier components of the velocity into a vector and including all the tracer displacements into another yields

(117) $$U={\left({\hat{v}}_{1},\ldots {\hat{v}}_{K}\right)}^{T},\;X={\left(x_{1,x},x_{1,y},\ldots ,x_{L,x},x_{L,y}\right)}^{T}.$$

Then, the coupled observation-filtering system is formed in a concise way,

$$\begin{array}{cc} \mathrm{Observations}:\;dX & =P_{X}\left(X\right)Udt+\Sigma_{x}dW_{X},\\ \mathrm{Underlying}\;\mathrm{flow}:\;dU & =-\Gamma Udt+F\left(t\right)dt+\Sigma_{u}dW_{u}. \end{array}$$

This is obviously a conditional Gaussian system with $u_{I}=X$ and $u_{\mathrm{II}}=U$.

In [62], multiscale turbulent flows were studied. To include both the (slow) geophysically balanced (GB) modes and (fast) gravity modes, the simple flow field in (116) was replaced by

(118) $$\begin{array}{cc} d{\hat{v}}_{\vec{k},B} & =\left(-d_{B}{\hat{v}}_{\vec{k},B}+f_{\vec{k},B}\left(t\right)\right)dt+\sigma_{\vec{k},B}dW_{\vec{k},B},\\ d{\hat{v}}_{\vec{k},\pm } & =\left(\;\left(-d_{g}+i\omega_{\vec{k},\pm }\right){\hat{v}}_{\vec{k},\pm }+f_{\vec{k},\pm }\left(t\right)\;\right)dt+\sigma_{\vec{k},\pm }dW_{\vec{k},\pm }, \end{array}$$

where $\omega_{\vec{k},\pm }\propto \pm \epsilon^{-1}$ with ϵ being a Rossby number [11,121].

In [63], a nonlinear coupling between GB and gravity modes as described in (115) were further included for generating the true signal while the filter adopts (118). This allows the understanding of model errors based on a novel information theoretic framework [63].

### 4.5. Other Low-Order Models for Filtering and Prediction

#### 4.5.1. Stochastic Parameterized Extended Kalman Filter Model

The stochastic parameterized extended Kalman filter (SPEKF) model [65,101] was introduced to filter and predict the highly nonlinear and intermittent turbulent signals as observed in nature:

(119) $$\begin{array}{cc} du & =\left((-\gamma +i\omega )u+F(t)+b\right)dt+\sigma_{u}dW_{u},\\ d\gamma & =-d_{\gamma }\left(\gamma -\hat{\gamma }\right)dt+\sigma_{\gamma }dW_{\gamma },\\ d\omega & =-d_{\omega }\left(\omega -\hat{\omega }\right)dt+\sigma_{\omega }dW_{\omega },\\ db & =-d_{b}\left(b-\hat{b}\right)dt+\sigma_{b}dW_{b}. \end{array}$$

In the SPEKF model (119), the process u(t) is driven by the stochastic damping γ(t), stochastic phase ω(t) and stochastic forcing correction b(t), all of which are specified as Ornstein–Uhlenbeck (OU) processes [66]. In (119), F(t) is a deterministic large-scale forcing, representing external effects such as the seasonal cycle. Physically, the variable u(t) in (119) represents one of the resolved modes (i.e., observable) in the turbulent signal, while γ(t),ω(t) and b(t) are hidden processes. In particular, γ(t),ω(t) and b(t) are surrogates for the nonlinear interaction between u(t) and other unobserved modes in the perfect model. The SPEKF model naturally belongs to conditional Gaussian filtering framework with the observed variable $u_{I}=u$ and filtering variables $u_{\mathrm{II}}={\left(\gamma ,\omega ,b\right)}^{T}$.

The nonlinear SPEKF system was first introduced in [65,101] for filtering multiscale turbulent signals with hidden instabilities and has been used for filtering and prediction in the presence of model error [1,20]. In addition to filtering and predicting intermittent signals from nature in the presence of model error [101,186,187,188], other important applications of using SPEKF to filter complex spatial-extended systems include stochastic dynamical superresolution [103] and effective filters for Navier–Stokes equations [104]. It has been shown that the SPEKF model has much higher skill than classical Kalman filters using the so-called mean stochastic model (MSM)

(120) $$du=\left(\left(-\hat{\gamma }+i\hat{\omega }\right)u+F\left(t\right)+\hat{b}\right)dt+\sigma_{u}dW_{u}$$

to capture the irregularity and intermittency in nature. Here, $\hat{\gamma },\hat{\omega }$ and $\hat{b}$ in (120) are the constant mean states of the damping, phase and forcing and therefore the MSM is a linear model with Gaussian statistics.

**Figure 18:**
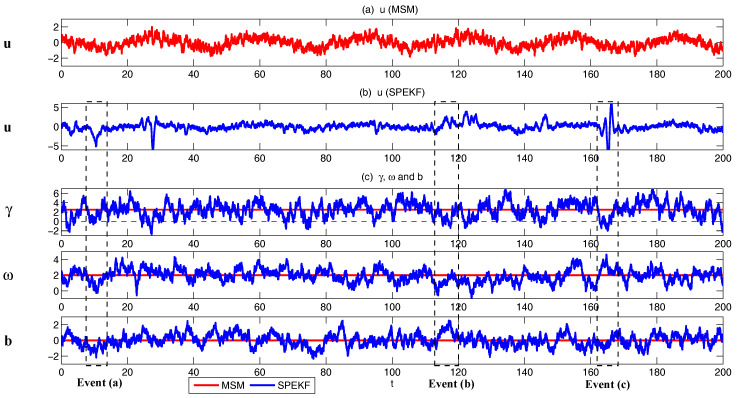
Simulations of the SPEKF model and MSM. (**a**) the observed variable *u* from MSM (120); (**b**) the observed variable *u* from SPEKF model; (**c**) hidden variables. The parameters are given in (121). The red curves are for MSM and blue ones are for SPEKF. The black dashed line in the γ process indicates the threshold of zero. When γ is below this threshold, γ becomes anti-damping and the associated signal of *u* tends to have exponential growth.

To provide more intuition, we show in Figure 18 the model simulation of SPEKF together with the MSM for comparison. The following parameters are adopted here

(121) $$\begin{array}{c} F\left(t\right)=2\;\exp\;\left(0.2t\right),\;\sigma_{u}=1,\;d_{\gamma }=0.8,\;\sigma_{\gamma }=2,\;\hat{\gamma }=2.5,\\ d_{\omega }=0.6,\;\sigma_{\omega }=1,\;\hat{\omega }=2,\;d_{b}=0.8,\;\sigma_{b}=1,\;\hat{b}=0. \end{array}$$

In Panel (a), the simulation of the MSM is shown. As expected, the signal of *u* has a large-scale time-periodic behavior due to the time-periodic forcing F(t). On top of the large-scale behavior, small fluctuations with nearly a constant oscillation is found, which is due to the constant phase $\hat{\omega }$. There is no intermittency since the damping $\hat{\gamma }=2.5>0$ always stabilizes the system.

On the other hand, with stochastic damping, stochastic phase and stochastic forcing, the variable u(t) has a rich dynamical behavior with intermittency and random oscillations. See Panels (b,c). In fact, when γ(t) becomes negative, the anti-damping results in an exponential increase of the signal of *u* and generates intermittency. In addition, the stochastic phase ω(t) leads to different phase oscillation frequency. For example, ω(t) at Event (a) is much smaller than that at Event (c), and therefore the oscillation at the phase of Event (c) is much faster. Furthermore, the stochastic forcing b(t) also modifies the signal of u(t). One example is shown in Event (b), where a large amplitude of b(t) forces the signal u(t) to go towards a positive value in the local area.

#### 4.5.2. An Idealized Surface Wind Model

The following idealized surface wind model is a version of the model for stochastic surface momentum budget [189]. Here, tendencies in the horizontal wind vector (u,v) averaged over a layer of thickness *h* are modelled as resulting from imbalances between the surface turbulent momentum flux (expressed using a bulk drag law with drag coefficient $c_{d}$, taken to be constant) and the ageostrophic difference between the pressure gradient and Coriolis forces:

$$\begin{array}{cc} \frac{du}{dt} & =\langle \Pi_{u}\rangle -\frac{c_{d}}{h}{\left(u^{2}+v^{2}\right)}^{1/2}u+\eta_{u}+\sigma_{u}{\dot{W}}_{u},\\ \frac{dv}{dt} & =-\frac{c_{d}}{h}{\left(u^{2}+v^{2}\right)}^{1/2}v+\eta_{v}+\sigma_{v}{\dot{W}}_{v},\\ \frac{d\eta_{u}}{dt} & =-\frac{1}{\tau }\eta_{u}+\frac{\sigma }{\tau }{\dot{W}}_{1},\\ \frac{d\eta_{v}}{dt} & =-\frac{1}{\tau }\eta_{v}+\frac{\sigma }{\tau }{\dot{W}}_{2}. \end{array}$$

The model assumes that tendencies associated with horizontal momentum advection are negligible and that the “large-scale” ageostrophic residual between the pressure gradient and Coriolis forces can be expressed as a mean $\left(\langle \Pi_{u}\rangle ,0\right)$ and fluctuations $\left(\eta_{u},\eta_{v}\right)$.

It is important to note that simply applying the white noise approximation for *u* and *v* (i.e., $\eta_{u}$ = $\eta_{v}=0$) has several limitations [190]. In particular, the white noise model requires unrealistically large values of *h* to produce serial dependence similar to that observed, and it is unable to account for the strong anisotropy in the autocorrelation structure of the wind components. Therefore, independent Ornstein–Uhlenbeck processes [66]$\left(\eta_{u},\eta_{v}\right)$ are included in the coupled system.

With extra additive noise with amplitudes $\sigma_{u},\sigma_{v}$, the coupled system belongs to conditional Gaussian framework with $u_{I}={\left(u,v\right)}^{T}$ and $u_{\mathrm{II}}={\left(\eta_{u},\eta_{v}\right)}^{T}$. The conditional Gaussian framework can be applied to filter the signal of $\left(\eta_{u},\eta_{v}\right)$, the filtered signal of which provides a clear view of the importance of such parameterization against simple white noise source.

## 5. Algorithms Which Beat the Curse of Dimension for Fokker–Planck Equation for Conditional Gaussian Systems: Application to Statistical Prediction

The Fokker–Planck equation is a partial differential equation (PDE) that governs the time evolution of the probability density function (PDF) of a complex system with noise [66,67]. For a general nonlinear dynamical system,

(122) du=F(u,t)dt+Σ(u,t)dW,

with state variables $u\in R^{N}$, noise matrix $\Sigma \in R^{N\times K}$ and white noise $W\in R^{K}$, the associated Fokker–Planck equation is given by

(123) $$\begin{array}{c} \frac{\partial }{\partial t}p\left(u,t\right)=-\nabla_{u}\left(F(u,t)p(u,t)\right)+\frac{1}{2}\nabla_{u}\cdot \nabla_{u}\left(Q\left(u,t\right)p\left(u,t\right)\right),\\ p_{t}{|}_{t=t_{0}}=p_{0}\left(u\right), \end{array}$$

with $Q=\Sigma \Sigma^{T}$. In many complex dynamical systems, including geophysical and engineering turbulence, neuroscience and excitable media, the solution of the Fokker–Planck equation in (123) involves strong non-Gaussian features with intermittency and extreme events [1,57,191]. In addition, the dimension of u in these complex systems is typically very large, representing a variety of variability in different temporal and spatial scales [1,121] (see also the examples in Section 4). Therefore, solving the high-dimensional Fokker–Planck equation for both the steady state and transient phases with non-Gaussian features is an important topic. However, traditional numerical methods such as finite element and finite difference as well as the direct Monte Carlo simulations of (122) all suffer from the curse of dimension [68,69]. Nevertheless, for the conditional Gaussian systems (1) and (2), efficient statistically accurate algorithms can be developed for solving the Fokker–Planck equation in high dimensions and thus beat the curse of dimension. Since the conditional Gaussian system is able to capture many salient features of the turbulent behavior, such algorithms are quite useful in uncertainty quantification, data assimilation and statistical prediction of turbulent phenomena in nature.

### 5.1. The Basic Algorithm with a Hybrid Strategy

Here, we state the basic efficient statistically accurate algorithms developed in [38]. The only underlying assumption here is that the dimension of $u_{I}$ is low while the dimension of $u_{\mathrm{II}}$ can be arbitrarily high.

First, we generate *L* independent trajectories of the variables $u_{I}$, namely $u_{I}^{1}\left(s\leq t\right),\ldots ,u_{I}^{L}\left(s\leq t\right)$, where *L* is a small number. This first step can be done by running a Monte Carlo simulation for the full system, which is computationally affordable with a small *L*. Then, different strategies are adopted to deal with the parts associated with $u_{I}$ and $u_{\mathrm{II}}$, respectively. The PDF of $u_{\mathrm{II}}$ is estimated via a parametric method that exploits the closed form of the conditional Gaussian statistics in (4) and (5),

(124) $$p\left(u_{\mathrm{II}}\left(t\right)\right)=\lim_{L\to \infty }\frac{1}{L}\sum_{i=1}^{L}p\left(u_{\mathrm{II}}\left(t\right)|u_{I}^{i}\left(s\leq t\right)\right).$$

See [38] for the details of the derivation of (124). Note that the limit L→∞ in (124) (as well as (125) and (126) below) is taken to illustrate the statistical intuition, while the estimator is the non-asymptotic version. On the other hand, due to the underlying assumption of the low dimensionality of $u_{I}$, a Gaussian kernel density estimation method is used for solving the PDF of the observed variables $u_{I}$,

(125) $$p\left(u_{I}\left(t\right)\right)=\lim_{L\to \infty }\frac{1}{L}\sum_{i=1}^{L}K_{H}\left(u_{I}\left(t\right)-u_{I}^{i}\left(t\right)\right),$$

where $K_{H}\left(\cdot \right)$ is a Gaussian kernel centered at each sample point $u_{I}^{i}\left(t\right)$ with covariance given by the bandwidth matrix H(t). The kernel density estimation algorithm here involves a “solve-the-equation plug-in” approach for optimizing the bandwidth [192] that works for any non-Gaussian PDFs. Finally, combining (124) and (125), a hybrid method is applied to solve the joint PDF of $u_{I}$ and $u_{\mathrm{II}}$ through a Gaussian mixture,

(126) $$p\left(u_{I}\left(t\right),u_{\mathrm{II}}\left(t\right)\right)=\lim_{L\to \infty }\frac{1}{L}\sum_{i=1}^{L}\left(K_{H}\left(u_{I}\left(t\right)-u_{I}^{i}\left(t\right)\right)\cdot p\left(u_{\mathrm{II}}\left(t\right)|u_{I}^{i}\left(s\leq t\right)\right)\right).$$

Practically, L∼O(100) is sufficient for the hybrid method to solve the joint PDF with $N_{I}\leq 3$ and $N_{\mathrm{II}}∼O\left(10\right)$. See [38] for the illustration of various concrete examples. Note that the closed form of the *L* conditional distributions in (124) can be solved in a parallel way due to their independence [38], which further reduces the computational cost. Rigorous analysis [193] shows that the hybrid algorithm (126) requires a much less number of samples as compared with traditional Monte Carlo method especially when the dimension of $u_{\mathrm{II}}$ is large.

### 5.2. Beating the Curse of Dimension with Block Decomposition

The basic algorithm in (126) succeeds in solving the Fokker–Planck equation with O(10) state variables. However, many complex turbulent dynamical systems in nature involve variables that have a much higher dimension (for example, those in Section 4.3). In such a situation, the update of the conditional covariance matrix becomes extremely expensive since the number of entries in the covariance matrix is $N^{2}$, where $n=N_{I}+N_{\mathrm{II}}$ is the total dimension of the variables. Therefore, new strategies are required to be incorporated into the basic algorithm in (126) in order to beat the curse of dimension. In this subsection, we develop an effective strategy with block decomposition [36], which combines with the basic algorithm and can efficiently solve the Fokker–Planck equation in much higher dimensions even with orders in the millions.

Consider the following decomposition of state variables:

$$u_{k}=\left(u_{I,k},u_{\mathrm{II},k}\right)\;\mathrm{with}\;u_{I,k}\in R^{N_{I},k}\;\;\mathrm{and}\;\;u_{\mathrm{II},k}\in R^{N_{\mathrm{II}},k},$$

where 1≤k≤K, $N_{I}=\sum_{k=1}^{K}N_{I,k}$ and $N_{\mathrm{II}}=\sum_{k=1}^{K}N_{\mathrm{II},k}$. Correspondingly, the full dynamics in (1) and (2) are also decomposed into *K* groups, where the variables on the left-hand side of the *k*-th group are $u_{k}$. In addition, for notation simplicity, we assume both $\Sigma_{I}$ and $\Sigma_{\mathrm{II}}$ are diagonal and thus the noise coefficient matrices associated with the equations of $u_{I,k}$ and $u_{\mathrm{II},k}$ are $\Sigma_{I,k}$ and $\Sigma_{\mathrm{II},k}$, respectively.

To develop efficient statistically accurate algorithms that beat the curse of dimension, the following two conditions are imposed on the coupled system.

*Condition 1:* In the dynamics of each $u_{k}$ in (1) and (2), the terms $A_{0,k}$ and $a_{0,k}$ can depend on all the components of $u_{I}$ while the terms $A_{1,k}$ and $a_{1,k}$ are only functions of $u_{I,k}$, namely,

(127) $$\begin{array}{ccc} A_{0,k}=A_{0,k}\left(t,u_{I}\right), & \; & a_{0,k}=a_{0,k}\left(t,u_{I}\right),\\ A_{1,k}=A_{1,k}\left(t,u_{I,k}\right), & \; & a_{1,k}=a_{1,k}\left(t,u_{I,k}\right). \end{array}$$

In addition, only $u_{\mathrm{II},k}$ interacts with $A_{1,k}$ and $a_{1,k}$ on the right-hand side of the dynamics of $u_{k}$. Therefore, the equation of each $u_{I}=\left(u_{I,k},u_{\mathrm{II},k}\right)$ becomes

(128) $$\begin{array}{cc} du_{I,k} & =\left[A_{0}\left(t,u_{I}\right)+A_{1}\left(t,u_{I,k}\right)u_{\mathrm{II},k}\right]dt+\Sigma_{I}\left(t,u_{I,k}\right)dW_{I}\left(t\right), \end{array}$$

(129) $$\begin{array}{cc} du_{\mathrm{II},k} & =\left[a_{0}\left(t,u_{I}\right)+a_{1}\left(t,u_{I,k}\right)u_{\mathrm{II},k}\right]dt+\Sigma_{\mathrm{II}}\left(t,u_{I,k}\right)dW_{\mathrm{II}}\left(t\right). \end{array}$$

Note that in (128) and (129) each $u_{k}$ is fully coupled with other $u_{k^{\prime }}$ for all $k^{\prime }\neq k$ through $A_{0}\left(t,u_{I}\right)$ and $a_{0}\left(t,u_{I}\right)$. There is no trivial decoupling between different state variables.

*Condition 2:* The initial values of $\left(u_{I,k},u_{\mathrm{II},k}\right)$ and $\left(u_{I,k^{\prime }},u_{\mathrm{II},k^{\prime }}\right)$ with $k\neq k^{\prime }$ are independent with each other.

These two conditions are not artificial and they are actually the salient features of many complex systems with multiscale structures [73], multilevel dynamics [119] or state-dependent parameterizations [103]. Under these two conditions, the conditional covariance matrix becomes block diagonal, which can be easily verified according to (5). The evolution of the conditional covariance of $u_{\mathrm{II},k}$ conditioned on $u_{I}$ is given by:

$$dR_{\mathrm{II},k}\left(t\right)=\left\{a_{1,k}R_{\mathrm{II},k}+R_{\mathrm{II},k}a_{1,k}^{*}+\left(\Sigma_{\mathrm{II},k}\Sigma_{\mathrm{II},k}^{*}\right)-\left(R_{\mathrm{II},k}A_{1,k}^{*}\right){\left(\Sigma_{I,k}\Sigma_{I,k}^{*}\right)}^{-1}{\left(R_{\mathrm{II},k}A_{1,k}^{*}\right)}^{*}\right\}dt,$$

which has no interaction with that of $R_{\mathrm{II},k^{\prime }}$ for all $k^{\prime }\neq k$ since $A_{0}$ and $a_{0}$ do not enter into the evolution of the conditional covariance. Notably, the evolutions of different $R_{\mathrm{II},k}$ with k=1,…,K can be solved in a parallel way and the computation is extremely efficient due to the small size of each individual block. This facilitates the algorithms to efficiently solve the Fokker–Planck equation in large dimensions.

Next, the structures of $A_{0,k}$ and $a_{0,k}$ in (127) allow the coupling among all the *K* groups of variables in the conditional mean according to (4). The evolution of ${\bar{u}}_{\mathrm{II},k}$, namely the conditional mean of $u_{\mathrm{II},k}$ conditioned on $u_{I}$, is given by

$$d{\bar{u}}_{\mathrm{II},k}\left(t\right)=\left[a_{0,k}+a_{1,k}{\bar{u}}_{\mathrm{II},k}\right]dt+R_{\mathrm{II},k}A_{1,k}^{*}{\left(\Sigma_{I,k}\Sigma_{I,k}^{*}\right)}^{-1}\left[du_{I,k}-\left(A_{0,k}\left(t,u_{I}\right)+A_{1,k}{\bar{u}}_{\mathrm{II},k}\right)dt\right].$$

Finally, let’s use concrete examples to illustrate the reduction in the computational cost with the block decomposition.

The first example is the two-layer L-96 model in (35). A typical choice of the number of grid points for the large scale variables $u_{i}$ (i=1,…,I) is I=40 and that of the equations for the small scale variables $v_{i,j}$ (j=1,…,J) is J=5. Thus, the total number of the small-scale variables $v_{i,j}$ is I×J=200 and the size of conditional covariance is $n_{total}={\left(I\times J\right)}^{2}=40,000$. Note that the two-layer L-96 model in (35) can be decomposed into the form in (128) and (129). Each block contains one element of $u_{k}$ and five elements of $v_{k,j}$ with j=1,…,5 and *k* fixed, and the number of the blocks is K=I=40. Therefore, the conditional covariance matrix associated with each block is of size 5×5=25 and the total number of the elements that need to be updated is $n_{reduced}=25\times 40=1000$, which is only 2.5% compared with the total elements in the full conditional covariance matrix!

Another example is the stochastically coupled FHN models. In fact, the block decomposition can be applied to all the versions (67)–(69) of the stochastically coupled FHN models as discussed in Section 4.2.1. Here, we illustrate the one in (69) with multiplicative noise. Let’s take n=500. Since the conditional statistics involves the information for both $v_{i}$ and $\gamma_{i}$ with i=1,…,500, the total size of the conditional covariance is $n_{total}={\left(2\times 500\right)}^{2}=1,000,000$. On the other hand, with the block decomposition that involves 500 blocks, where each block is of size 2×2=4 and contains the information of only one $v_{i}$ and one $\gamma_{i}$, the total number of the elements that need to be updated is $n_{reduced}=500\times 4=2000$. Clearly, the computational cost in the conditional covariance update with the block decomposition is only $n_{reduced}/n_{total}=0.5\%$ compared with a full update! In Section 5.5, the statistical prediction of both the two-layer L-96 model and the stochastically coupled FHN model with multiplicative noise using the efficient statistically accurate algorithms will be demonstrated.

### 5.3. Statistical Symmetry

As was discussed in the previous two subsections, the hybrid strategy and the block decomposition provide an extremely efficient way to solve the high-dimensional Fokker–Planck equation associated with the conditional Gaussian systems. In fact, the computational cost in the algorithms developed above can be further reduced if the coupled system (1) and (2) has statistical symmetry [36]:

(130) $$p\left(u_{I,k}\left(t\right),u_{\mathrm{II},k}\left(t\right)\right)=p\left(u_{I,k^{\prime }}\left(t\right),u_{\mathrm{II},k^{\prime }}\left(t\right)\right),\;\mathrm{for}\;\mathrm{all}\;k\mathrm{and}\;k^{\prime }.$$

Namely, the statistical features for variables with different *k* are the same. The statistical symmetry is often satisfied when the underlying dynamical system represents a discrete approximation of some PDEs in a periodic domain with nonlinear advection, diffusion and homogeneous external forcing [2,20].

With the statistical symmetry, collecting the conditional Gaussian ensembles $N({\bar{u}}_{\mathrm{II},k}\left(t\right),R_{\mathrm{II},k}\left(t\right))$ for a specific *k* in *K* different simulations is equivalent to collecting that for all *k* with 1 ≤ *k* ≤ *K* in a single simulation. This also applies to $N(u_{I}^{i}\left(t\right),H\left(t\right))$ that are associated with $u_{I}$. Therefore, the statistical symmetry implies that the effective sample size is $L^{\prime }=KL$, where *K* is the number of the group variables that are statistically symmetric and *L* is the number of different simulations of the coupled systems via Monte Carlo. If *K* is large, then a much smaller *L* is needed to reach the same accuracy as in the situation without utilizing statistical symmetry, which greatly reduces the computational cost.

Below, we discuss the details of constructing the joint PDFs obtaining from the algorithms with statistical symmetry. First, we adopt the one-dimensional case for the convenience of illustration. The method can be easily extended to systems with multivariables and multidimensions. Denote ${\bar{u}}_{1},{\bar{u}}_{2},\ldots ,{\bar{u}}_{K}$ the mean values of the Gaussian ensembles at different grid points and the associated variance are $R_{1},\ldots ,R_{K}$. For simplicity, we only take one full run of the system. Therefore, the total number of Gaussian ensembles is *K*. Clearly, the 1D PDFs $p(u_{i})$ at different grid points are the same and are given by

(131) $$p\left(u_{i}\right)=\lim_{k\to \infty }\frac{1}{K}\sum_{k=1}^{K}N\left({\bar{u}}_{k},R_{k}\right),i=1,\ldots ,K.$$

The limit is taken for statistical intuition while a finite and small *K* is adopted in practice.

Now, we discuss the construction of the joint PDFs. We use the 2D joint PDF $p(u_{1},u_{2})$ as an illustration. The joint PDF is a Gaussian mixture with *K* Gaussian ensembles, where the mean of each 2D Gaussian ensemble is

(132) $$\mu_{1}=\left(\begin{array}{c} {\bar{u}}_{1}\\ {\bar{u}}_{2} \end{array}\right),\;\mu_{1}=\left(\begin{array}{c} {\bar{u}}_{2}\\ {\bar{u}}_{3} \end{array}\right)\;\ldots \;\mu_{K-1}=\left(\begin{array}{c} {\bar{u}}_{K-1}\\ {\bar{u}}_{K} \end{array}\right),\;\mu_{K}=\left(\begin{array}{c} {\bar{u}}_{K}\\ {\bar{u}}_{1} \end{array}\right),$$

and the covariance matrix is given by

(133) $$R_{1}=\left(\begin{array}{cc} R_{1} & \\ & R_{2} \end{array}\right),\;R_{2}=\left(\begin{array}{cc} R_{2} & \\ & R_{3} \end{array}\right)\;\ldots \;R_{K-1}=\left(\begin{array}{cc} R_{K-1} & \\ & R_{K} \end{array}\right),\;R_{K}=\left(\begin{array}{cc} R_{K} & \\ & R_{1} \end{array}\right).$$

It is clear from the construction of the ensemble mean in (132) that the subscript in the second component equals that of the first component plus one. That is, the first component of each $\mu_{k},k=1,\ldots ,K$ is treated as $u_{1}$ due to the statistical symmetry and the second component is treated as the corresponding $u_{2}$ associated with each *k*. The diagonal covariance matrix in (133) comes from the fact that each sample point is independent with each other. This is also true and more obvious for the block diagonal conditional covariance. Notably, the diagonal covariance matrix of each ensemble does not mean that the correlation between $u_{1}$ and $u_{2}$ is completely ignored. The correlation is reflected regarding how the points of ensemble means $\mu_{k},k=1,\ldots ,K$ are distributed.

### 5.4. Quantifying the Model Error Using Information Theory

Before we apply the efficient statistically accurate algorithms to statistical prediction, we introduce a measurement for quantifying errors. The natural way to quantify the error in the predicted PDF related to the truth is through an information measure, namely the relative entropy (or Kullback–Leibler divergence) [2,187,194,195,196,197]. The relative entropy is defined as

(134) $$P\left(p\left(u\right),p^{M}\left(u\right)\right)=\int p\left(u\right)\ln\frac{p(u)}{p^{M}\left(u\right)}du,$$

where p(u) is the true PDF and $p^{M}\left(u\right)$ is the predicted one from the efficient statistically accurate algorithms. This asymmetric functional on probability densities $P(p,p^{M})\geq 0$ measures lack of information in $p^{M}$ compared with *p* and has many attractive features. First, $P(p,p^{M})\geq 0$ with equality if and only if $p=p^{M}$. Secondly, $P(p,p^{M})$ is invariant under general nonlinear changes of variables. Notably, the relative entropy is a good indicator of quantifying the difference in the tails of the two PDFs, which is particularly crucial in the turbulent dynamical systems with intermittency and extreme events. On the other hand, the traditional ways of quantifying the errors, such as the relative error $∥p-p^{M}∥/∥p∥$, usually underestimate the lack of information in the PDF tails.

### 5.5. Applications to Statistical Prediction

Now, we apply the efficient statistically accurate algorithms developed in the previous subsections to statistical prediction. Two examples of nonlinear complex turbulent dynamical systems in high dimensions are shown below.

The first example is the stochastically coupled FHN model in (69) with multiplicative noise (Section 4.2.1) with n=500,

(135) $$\begin{array}{cc} \epsilon du_{i} & =\left(d_{u}\left(u_{i+1}+u_{i-1}-2u_{i}\right)+u_{i}-\frac{1}{3}u_{i}^{3}-v_{i}\right)dt+\sqrt{\epsilon }\delta_{1}dW_{u_{i}},\\ dv_{i} & =\left(\gamma_{i}u_{i}+a\right)dt+\delta_{2}dW_{v_{i}},\\ d\gamma_{i} & =-d_{\gamma_{i}}\left(\gamma_{i}-{\hat{\gamma }}_{i}\right)dt+\sigma_{\gamma_{i}}dW_{\gamma_{i}},\;i=1,\ldots ,N. \end{array}$$

The parameters are given by (71).

The second example is the two-layer L-96 model in (35) (Section 4.1.1) with I=40 and J=5. The parameters are given by (36) and (37):

(136) $$\begin{array}{cc} du_{i} & =\left(u_{i-1}\left(u_{i+1}-u_{i-2}\right)+\sum_{j=1}^{J}\gamma_{i,j}u_{i}v_{i,j}-{\bar{d}}_{i}u_{i}+F\right)dt+\sigma_{u}dW_{u_{i}},\;i=1,\ldots ,I,\\ dv_{i,j} & =\left(-d_{v_{i,j}}v_{i,j}-\gamma_{j}u_{i}^{2}\right)dt+\sigma_{i,j}dW_{v_{i,j}},\;j=1,\ldots ,J. \end{array}$$

**Figure 19:**
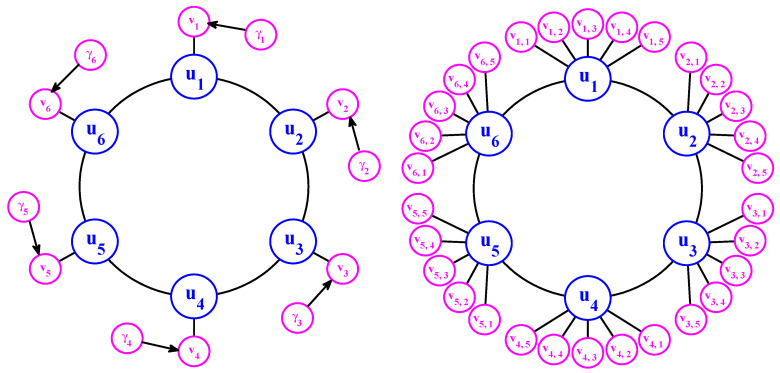
Schematic illustration of the coupling between different variables in the stochastically coupled FHN model (135) (**left**) and the two-layer L-96 model (136) (**right**). For illustration purposes, I=n=6 is used in this figure while I=40 and n=500 in the model simulations. All the variables are coupled to each other with no trivial decoupling.

Both the stochastically coupled FHN model (135) and the two-layer L-96 model (136) satisfy the model structure as described in Section 5.2 such that the block decomposition applies. Figure 19 provides a schematic illustration of the coupling between different variables in these models (for illustration purposes, I=n=6 is used in the figure). The multiscale and layered structures can be easily observed. It is also clear that all the variables are coupled to each other with no trivial decoupling. Note that the stochastically coupled FHN model (135) with the given parameters here also satisfies the statistical symmetry while the two-layer L-96 model (136) is inhomogeneous.

#### 5.5.1. Application to the Stochastically Coupled FHN Model with Multiplicative Noise Using Statistical Symmetry

Here, we illustrate the statistical prediction of the stochastically coupled FHN model with multiplicative noise (135), where the number of grid points in space is n=500. The noise coefficient in the $\gamma_{i}$ process is $\sigma_{\gamma_{i}}=0.6$ for all *i*. The other parameters are the same as those in (71) and the noise coefficients are the same at different grid points *i*, namely

(137) $$\epsilon =0.01,\;\delta_{1}=0.2,\;\delta_{2}=0.4,\;a=1.05,\;d_{\gamma_{i}}=1,\;{\hat{\gamma }}_{i}=\hat{\gamma }=1,\;\sigma_{\gamma_{i}}=\sigma_{\gamma }=0.6.$$

We also adopt homogeneous initial conditions $u_{i}\left(0\right)=-2$ and $v_{i}\left(0\right)=0.5$ for all i=1,…,N. Therefore, the model satisfies the statistical symmetry. The model simulations were shown in Figure 12 and Figure 13, where intermittent events are observed in both the fields of *u* and *v*.

**Figure 20:**
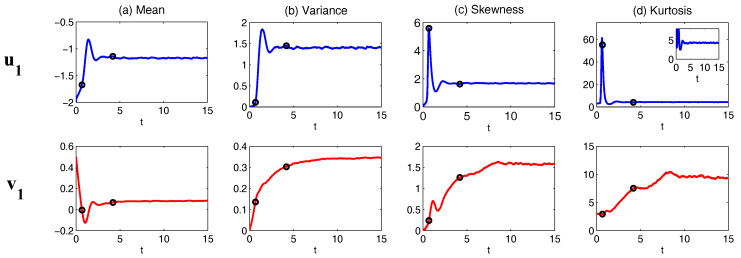
Time evolution of the first four moments ((**a**) mean; (**b**) variance; (**c**) skewness and (**d**) kurtosis) associated with $u_{1}$ and $v_{1}$ of the stochastically coupled FHN model with multiplicative noise (135), where n=500. Due to the statistical symmetry, the evolutions of these moments for different $u_{i}$ and $v_{i}$ are the same as $u_{1}$ and $v_{1}$. The dots at t=0.68 and t=4.2 indicate the time instants that the statistical prediction using the efficient statistically accurate algorithms are tested. Note that t=0.68 corresponds to the time instant that *u* arrives at its most non-Gaussian phase while t=4.2 is a non-Gaussian phase where *u* is nearly the statistical equilibrium while *v* is still transient.

**Figure 21:**
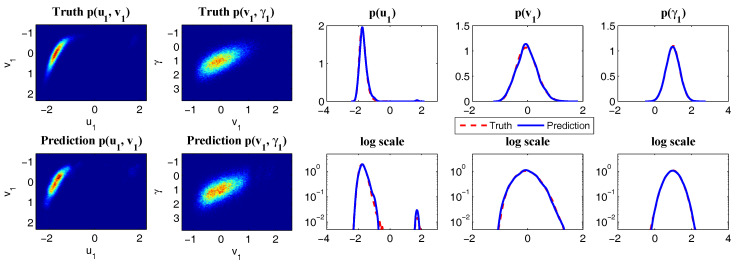
Statistical prediction of the 1D and 2D PDFs of the stochastically coupled FHN model with multiplicative noise (135) at a transit phase t=0.68, where n=500. Note that there is a small but non-zero probability around $u_{1}=2$ (see the subpanel with the logarithm scale).

**Figure 22:**
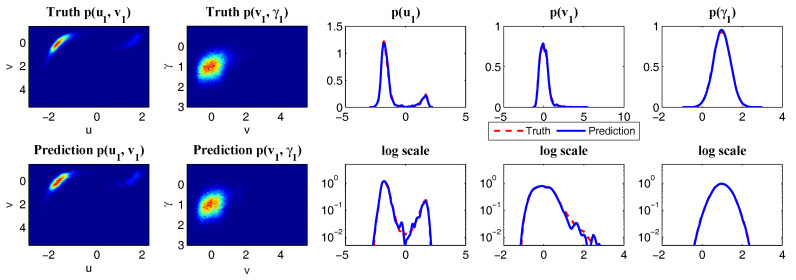
Statistical prediction of the 1D and 2D PDFs of the stochastically coupled FHN model with multiplicative noise (135) at a transit phase t=4.2, where n=500.

Figure 20 shows the time evolution of the first four moments associated with $u_{1}$ and $v_{1}$. Note that due to the statistical symmetry, the evolutions of these moments for different $u_{i}$ and $v_{i}$ are the same as $u_{1}$ and $v_{1}$. The dot at t=0.68 indicates the time instant that *u* arrives at its most non-Gaussian phase while t=4.2 is a non-Gaussian phase, where *u* is nearly the statistical equilibrium while *v* is still transient. In Figure 21 and Figure 22, the statistical prediction of the 1D marginal and 2D joint PDFs are shown at these two time instants. At t=0.68, most of the probability density of $u_{1}$ is concentrated around $u_{1}=-1.8$. However, there is a small but non-zero probability around $u_{1}=2$ (see the subpanel with the logarithm scale), which leads to large skewness and kurtosis of the statistics of $u_{1}$ as observed in Figure 20. The efficient statistically accurate algorithms succeed in predicting the PDFs at this transient phase and are able to accurately recover the statistics in predicting such extreme events. A similar performance is found in Figure 22, where the bimodal PDF of $u_{1}$, the highly skewed PDF of $v_{1}$ and the Gaussian one of $\gamma_{1}$ are all predicted with high accuracy. The fully non-Gaussian joint PDFs $p(u_{1},v_{1})$ and $p(v_{1},\gamma_{1})$ are also predicted with almost no bias. These results indicate the skillful behavior of the algorithms developed above.

Note that the total dimension of the stochastically coupled FHN model with multiplicative noise (135) is 3N=1500. Due to the statistical symmetry, the effective sample size in the statistical prediction as shown in Figure 21 and Figure 22 is $L^{\prime }=NL=500L$, where *L* is the number of repeated simulations of the systems. In fact, the simulations in the efficient statistically accurate algorithms are achieved with only L=1. This means we only run the model (135) once and apply the efficient statistically accurate algorithms that provide the accurate results in these figures. Therefore, the statistical prediction here is extremely cheap! On the other hand, we take $L_{C}=300$ in Monte Carlo simulations and again use statistical symmetry to generate the true PDFs and therefore the effective sample size in Monte Carlo simulation is $L_{C}^{\prime }=NLC=150,000$.

#### 5.5.2. Application to the Two-Layer L-96 Model with Inhomogeneous Spatial Structures

Now we apply the efficient statistically accurate algorithms to predict the two-layer L-96 model in (136). Here, I=40 and J=5. The parameters are given by (36) and (37). The model behavior with different forcing *F* is shown in Figure 2, Figure 3 and Figure 4.

Although the two-layer inhomogeneous L-96 model in (136) has no statistical symmetry, the model structure nevertheless allows the effective block decomposition. Below, L=500 trajectories of each variable $u_{i}$ are simulated from (136) to implement the efficient statistically accurate algorithms. As a comparison, a direct Monte Carlo method requires $L_{C}$ = 150,000 samples for each of the 240 variables for an accurate estimation of at least the one-point statistics. This means the total number of samples is around $4\times 10^{7}$! For an efficient calculation of the truth, we focus only on the statistical equilibrium state here, but the algorithms are not restricted to the equilibrium statistics. The true PDFs are calculated using the Monte Carlo samples over a long time series in light of the ergodicity while the recovered PDFs from the efficient statistically accurate algorithms are computed at t=25.

**Figure 23:**
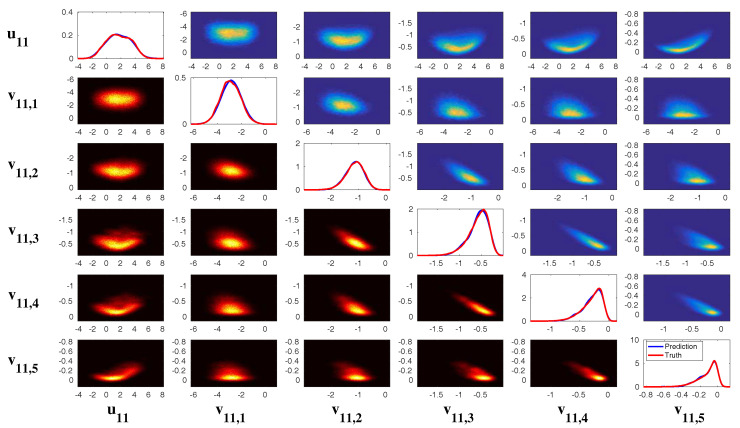
Inhomogeneous two-layer L-96 model (35) with *F* = 5. The other parameters are given in (36) and (37). Comparison of the 1D and 2D true and recovered PDFs at i=11. The diagonal subpanels here show the 1D marginal PDFs of $u_{11}$ and $v_{11,1},\ldots ,v_{11,5}$, where the blue one is the prediction and the red one is the truth. In this figure with 6×6 subpanels, the $\left(k_{i},k_{j}\right)$-subpanel with $k_{i}>k_{j}$ (below the diagonal panel) shows the true 2D PDF using a large number of Monte Carlo samples (red colormap) while the one with $k_{i}<k_{j}$ (above the diagonal panels) shows the predicted one using the efficient statistically accurate algorithm (blue colormap). The $\left(k_{i},k_{j}\right)$-panel is compared with the (j,i)-panel. Note that for the simplicity of comparison the labels $u_{11}$ and $v_{11,1},\ldots ,v_{11,5}$ on the bottom and left of the (i,j)-panel correspond to the *x*-axis and *y*-axis of the truth and the *y*-axis and *x*-axis of the predicted PDFs.

**Figure 24:**
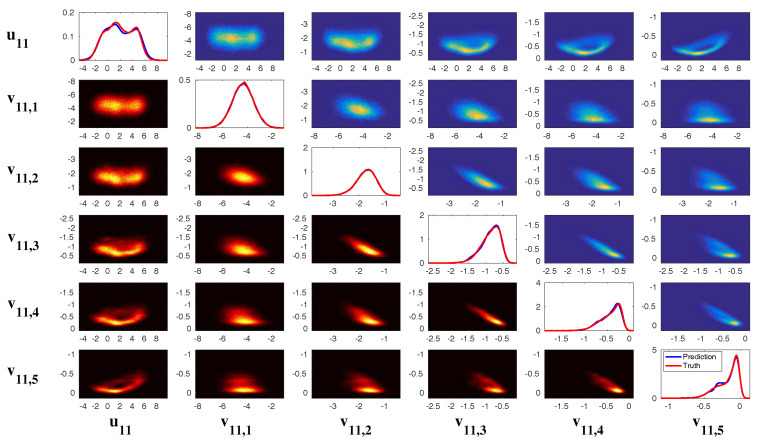
Inhomogeneous two-layer L-96 model (35), similar to Figure 23 but with *F* = 8.

**Figure 25:**
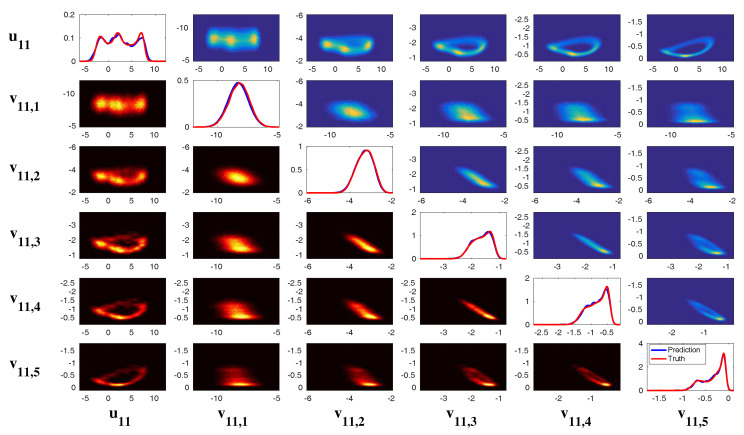
Inhomogeneous two-layer L-96 model (35), similar to Figure 23 but with *F* = 16.

**Figure 26:**
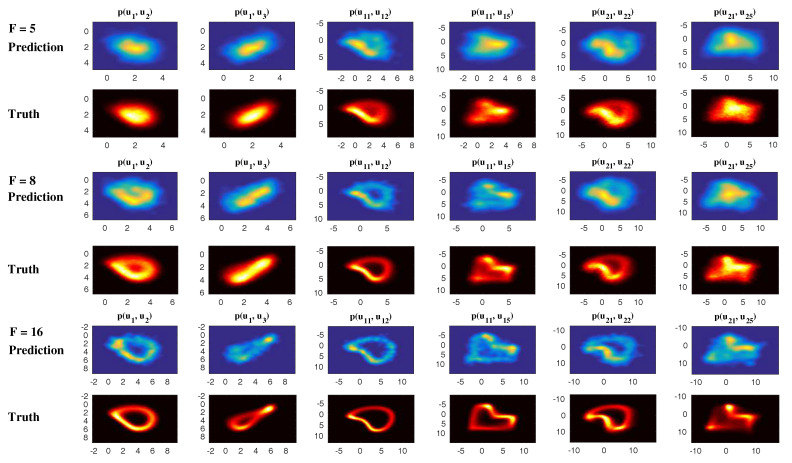
Inhomogeneous two-layer L-96 model (35) with F=5 (**top**); F=8 (**middle**) and F=16 (**bottom**). The other parameters are given in (36) and (37). Comparison of the joint PDFs of the large scale $p(u_{i_{1}},u_{i_{2}})$ with different $i_{1}$ and $i_{2}$.

**Figure 27:**
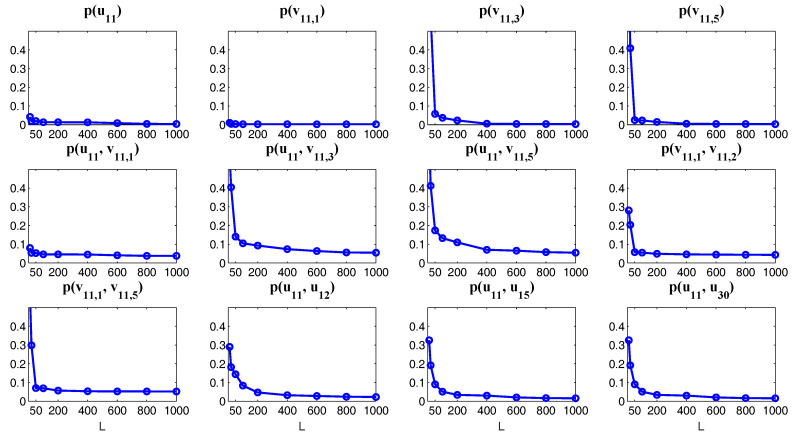
Inhomogeneous two-layer L-96 model (35) with F=5. Error in the predicted PDF as a function of the sample points *L* compared with the truth. The error is computed via the relative entropy (134).

In Figure 23, Figure 24 and Figure 25, we show the statistical prediction in all the three regimes with F=5,8 and 16, respectively, using the efficient statistically accurate algorithm and compare them with the truth. Here, we only show the results at i=11. Qualitative similar results are found at other grid points. In these figures, the diagonal subpanels here show the 1D marginal PDFs of $u_{11}$ and $v_{11,1},\ldots ,v_{11,5}$, where the blue one is the prediction and the red one is the truth. The $\left(k_{i},k_{j}\right)$-subpanel with $k_{i}>k_{j}$ (below the diagonal panel) shows the true 2D PDF using a large number of Monte Carlo samples (red colormap) while the one with $k_{i}<k_{j}$ (above the diagonal panels) shows the predicted one using the efficient statistically accurate algorithm (blue colormap). The $\left(k_{i},k_{j}\right)$-panel is compared with the (j,i)-panel. Note that for the simplicity of comparison the labels $u_{11}$ and $v_{11,1},\ldots ,v_{11,5}$ on the bottom and left of the (i,j)-panel correspond to the *x*-axis and *y*-axis of the truth and the *y*-axis and *x*-axis of the predicted PDFs. On the other hand, Figure 26 shows several 2D joint PDFs of the large scale $p(u_{i_{1}},u_{i_{2}})$ with different $i_{1}$ and $i_{2}$. It is clear that all the strong non-Gaussian features, including highly skewed, multimodal and fat-tailed distribution, are accurately predicted by the statistically accurate algorithm. Finally, Figure 27 shows the error in the predicted PDF as a function of the sample points *L* compared with the truth, where the error is computed via the relative entropy (134). In fact, with L∼O(100) samples in the efficient statistically accurate algorithms, the statistical prediction has already been accurate enough. Note that the small non-zero asymptotic values as a function of *L* in the error curves is due to the numerical error in computing the integral of the relative entropy (134) as well as the sampling in the Monte Carlo simulations.

It is worthwhile pointing out that although only the predicted one-point and two-point statistics are shown in both the examples here, the algorithms can actually provide an accurate estimation of the full joint PDF of $u_{\mathrm{II}}$, using a small number of samples. This is because the sample size in these algorithms does not grow exponentially as the dimension of $u_{\mathrm{II}}$, which is fundamentally different from Monte Carlo methods. See [193] for a theoretical justification.

## 6. Multiscale Data Assimilation, Particle Filters, Conditional Gaussian Systems and Information Theory for Model Errors

### 6.1. Parameter Estimation

The conditional Gaussian system (1) and (2) and its closed analytic formula for solving the conditional statistics (4) and (5) also provide a framework for parameter estimation. In fact, $u_{\mathrm{II}}$ can be written as

$$u_{\mathrm{II}}=\left({\tilde{u}}_{\mathrm{II}},\Lambda \right),$$

where $u_{\mathrm{II}}$ in $R^{{\tilde{N}}_{2}}$ is physical process variables and $\Lambda =\left(\lambda_{1},\lambda_{2},\ldots ,\lambda_{p}\right)\in R^{N_{2,p}}$ denotes the model parameters. Here, $N_{2}={\tilde{N}}_{2}+N_{2,p}$. Rewriting the conditional Gaussian system (1) and (2) in terms of $u_{\mathrm{II}}=\left({\tilde{u}}_{\mathrm{II}},\Lambda \right)$ yields

(138) $$\begin{array}{cc} du_{I} & =\left[A_{0}\left(t,u_{I}\right)+A_{1}\left(t,u_{I}\right){\tilde{u}}_{\mathrm{II}}+A_{1,\lambda }\left(t,u_{I}\right)\Lambda \right]dt+\Sigma_{I}\left(t,u_{I}\right)dW_{I}\left(t\right), \end{array}$$

(139) $$\begin{array}{cc} du_{\mathrm{II}} & =\left[a_{0}\left(t,u_{I}\right)+a_{1}\left(t,u_{I}\right){\tilde{u}}_{\mathrm{II}}+a_{1,\lambda }\left(t,u_{I}\right)\Lambda \right]dt+\Sigma_{\mathrm{II}}\left(t,u_{I}\right)dW_{\mathrm{II}}\left(t\right). \end{array}$$

The Equation (138) and (139) includes both the dynamics of ${\tilde{u}}_{\mathrm{II}}$ and those of Λ. Both the physical process variables ${\tilde{u}}_{\mathrm{II}}$ and the parameters Λ are coupled with any highly nonlinear functions of $u_{I}$. Nevertheless, any monomial involving both ${\tilde{u}}_{\mathrm{II}}$ and Λ is not allowed since otherwise the conditional Gaussian structure will break. Notably, although ${\tilde{u}}_{\mathrm{II}}$ and Λ are named as model states and parameters, they can also be understood as stochastic parameterization and simplification of complex physical process via bulk average, respectively. Therefore, ${\tilde{u}}_{\mathrm{II}}$ and Λ often share the same role in providing extra information of $u_{I}$ that leads to various non-Gaussian characteristics. This is a typical feature in multiscale complex turbulent dynamical systems.

Below, we provide different frameworks for parameter estimation. Let’s temporally ignore ${\tilde{u}}_{\mathrm{II}}$ for notation simplicity and the dynamics is given by

(140) $$du_{I}=\left[A_{0}\left(t,u_{I}\right)+A_{1,\lambda }\left(t,u_{I}\right)\Lambda^{*}\right]dt+\Sigma_{I}\left(t,u_{I}\right)dW_{I}\left(t\right),$$

where $\Lambda^{*}$ is the true parameter.

#### 6.1.1. Direct Parameter Estimation Algorithm

Since Λ are constant parameters, it is natural to augment the dynamics with the following relationship,

(141) $$\begin{array}{cc} du_{I} & =\left[A_{0}\left(t,u_{I}\right)+A_{1,\lambda }\left(t,u_{I}\right)\Lambda \right]dt+\Sigma_{I}\left(t,u_{I}\right)dW_{I}\left(t\right), \end{array}$$

(142) $$\begin{array}{cc} d\Lambda & =0, \end{array}$$

where an initial uncertainty for the parameter Λ is assigned. According to (4) and (5), the time evolutions of the mean ${\bar{u}}_{\mathrm{II}}$ and covariance $R_{\mathrm{II}}$ of the estimate of Λ are given by

(143) $$\begin{array}{cc} d{\bar{u}}_{\mathrm{II}}\left(t\right)= & \left(R_{\mathrm{II}}A_{1}^{*}\left(t,u_{I}\right)\right){\left(\Sigma_{I}\Sigma_{I}^{*}\right)}^{-1}\left(t,u_{I}\right)\left[du_{I}-\left(A_{0}\left(t,u_{I}\right)+A_{1}\left(t,u_{I}\right){\bar{u}}_{\mathrm{II}}\right)dt\right], \end{array}$$

(144) $$\begin{array}{cc} dR_{\mathrm{II}}\left(t\right)= & -\left(R_{\mathrm{II}}A_{1}^{*}\left(t,u_{I}\right)\right){\left(\Sigma_{I}\Sigma_{I}^{*}\right)}^{-1}\left(t,u_{I}\right){\left(R_{\mathrm{II}}A_{1}^{*}\left(t,u_{I}\right)\right)}^{*}dt. \end{array}$$

The formula in (144) indicates that $R_{\mathrm{II}}=0$ is a solution, plugging which into (143) results in ${\bar{u}}_{\mathrm{II}}=\Lambda^{*}$. This means, by knowing the perfect model, the estimated parameters in (141) and (142) and (143) and (144) under certain conditions will converge to the truth.

**Figure 28:**
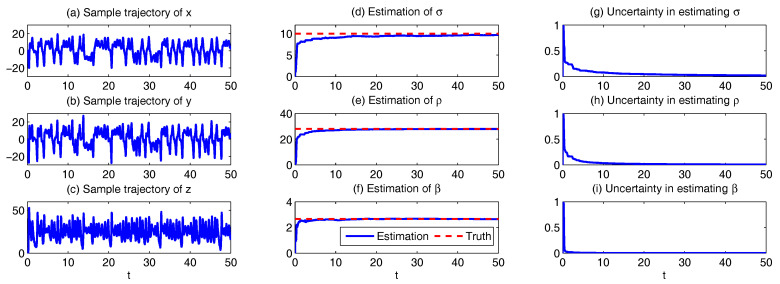
Parameter estimation of the noisy L-63 system using the direct approach (145) with $\sigma_{x}$ = $\sigma_{y}$ = $\sigma_{z}$ = 5. (**a**–**c**) sample trajectories of x,y,z which are used as observations; (**d**–**f**) time evolution of the estimated parameters and the truth (red); (**g**–**i**) the uncertainty evolution of each estimated parameter.

**Figure 29:**
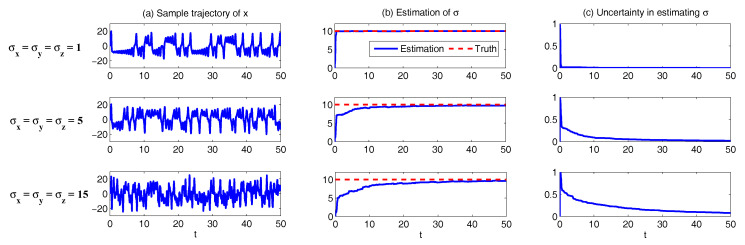
Parameter estimation of the noisy L-63 system using the direct approach (145) with $\sigma_{x}$ = $\sigma_{y}$ = $\sigma_{z}$ = 1, 5 and 15 for the three rows. For illustration purposes, only the trajectory of *x* and the estimation of σ is shown in all the three cases. (**a**) sample trajectory of *x*; (**b**,**c**) mean estimation of σ and the associated uncertainty.

As a simple test example, we consider estimating the three parameters σ,ρ and γ in the noisy L-63 model (31) with ρ=28,σ=10,β=8/3. Putting into the framework (141) and (142), the augmented system becomes

(145) $$\begin{array}{cc} dx & =\sigma \left(y-x\right)dt+\sigma_{x}dW_{x},\\ dy & =\left(x(\rho -z)-y\right)dt+\sigma_{y}dW_{y},\\ dz & =\left(xy-\beta z\right)dt+\sigma_{z}dW_{z},\\ d\sigma & =0,\\ d\rho & =0,\\ d\beta & =0, \end{array}$$

where $u_{I}={\left(x,y,z\right)}^{T}$ and $\Lambda ={\left(\sigma ,\rho ,\beta \right)}^{T}$. Figure 28 shows the parameter estimation skill with $\sigma_{x}=\sigma_{y}=\sigma_{z}=5$. Here, the initial guess of all the three parameters are ρ=σ=β=0 and the initial uncertainty is a 3×3 identity matrix. It is clear that the estimated parameter converge quickly to the truth and the corresponding uncertainty in the estimated parameters goes to zero. In Figure 29, the parameter estimation skill with different noise level is compared. When the noise level $\sigma_{x},\sigma_{y}$ and $\sigma_{z}$ in the observed signals become larger, the convergence rate becomes slower.

In [28], systematic studies of both one-dimensional linear and nonlinear systems have revealed that the convergence rate depends on different factors, such as the observability, the signal-to-noise ratio and the initial uncertainty. In particular, both theoretical analysis and numerical simulations in [28] showed that the convergence rate can be extremely slow and sometimes with an undesirable initial guess the estimation even converges to a wrong solution for some highly nonlinear systems. Thus, alternative parameter estimation scheme is required.

#### 6.1.2. Parameter Estimation Using Stochastic Parameterized Equations

Instead of augmenting the equation in a trivial but natural way for the parameters as shown in (142), a new approach of the augmented system can be formed in the following way [28]:

(146) $$\begin{array}{cc} du_{I} & =\left[A_{0}\left(t,u_{I}\right)+A_{1,\lambda }\left(t,u_{I}\right)\Lambda \right]dt+\Sigma_{I}\left(t,u_{I}\right)dW_{I}\left(t\right) \end{array}$$

(147) $$\begin{array}{cc} d\Lambda & =\left[c_{1}\Lambda +c_{2}\right]dt+\sigma_{\Lambda }dW_{\Lambda }\left(t\right). \end{array}$$

Here, $c_{1}$ is a negative-definite diagonal matrix, $c_{2}$ is a constant vector and $\sigma_{\Lambda }$ is a diagonal noise matrix. Different from (142), a stochastic parameterized equation is used in (147) to describe the time evolution of each component of the parameter $\Lambda_{i}$. This approach is motivated from the stochastic parameterized extended Kalman filter model [64,65] as discussed in Section 4.5.1. The stochastic parameterized equations in (147) serve as the prior information of the parameter estimation. Although certain model error will be introduced in the stochastic parameterized equations due to the appearance of $c_{1}$, $c_{2}$ and $\sigma_{\Lambda }$, it has shown in [28] that the convergence rate will be greatly accelerated. In fact, in linear models, rigorous analysis reveals that the convergence rate using stochastic parameterized Equation (146) and (147) is exponential while that using the direct method (146) and (147) is only algebraic.

Now, we apply the parameter estimation using stochastic parameterized Equation (146) and (147) for the noisy L-63 model. The augmented system reads,

(148) $$\begin{array}{cc} dx & =\sigma \left(y-x\right)dt+\sigma_{x}dW_{x},\\ dy & =\left(x(\rho -z)-y\right)dt+\sigma_{y}dW_{y},\\ dz & =\left(xy-\beta z\right)dt+\sigma_{z}dW_{z},\\ d\sigma & =-d_{\sigma }\left(\sigma -\hat{\sigma }\right)+\sigma_{\sigma }dW_{\sigma },\\ d\rho & =-d_{\rho }\left(\rho -\hat{\rho }\right)+\sigma_{\rho }dW_{\rho },\\ d\beta & =-d_{\beta }\left(\beta -\hat{\beta }\right)+\sigma_{\beta }dW_{\beta }. \end{array}$$

The same initial values are taken as those in the direct method. Recall in Figure 29 that the system with a large observational noise $\sigma_{x}=\sigma_{y}=\sigma_{z}=15$ leads to a slow convergence rate. Below, we focus on this case and use the parameter estimation scheme (148) to improve the result. The parameters in the stochastic parameterized equation are chosen as follows:

(149) $$\begin{array}{c} \hat{\sigma }=10+2=12,\;\hat{\rho }=28+5.6=33.6,\;\hat{\beta }=8/3+1.6/3=3.2,\\ d_{\sigma }=d_{\rho }=d_{\beta }=0.5,\;\sigma_{\sigma }=2,\;\sigma_{\rho }=5.6,\;\sigma_{\beta }=1.6/3. \end{array}$$

Here, we introduce 20% errors in all the mean states $\hat{\sigma },\hat{\rho }$ and $\hat{\beta }$. The variance of the stochastic parameters is given by $\sigma_{\sigma }^{2}/\left(2d_{\sigma }\right),\sigma_{\rho }^{2}/\left(2d_{\rho }\right)$ and $\sigma_{\beta }^{2}/\left(2d_{\beta }\right)$, respectively. Therefore, the truth is located at one standard deviation (square root of the variance) of the mean state. The decorrelation time of all the process is $1/d_{\sigma }=1/d_{\rho }=1/d_{\beta }=2$ time units.

**Figure 30:**
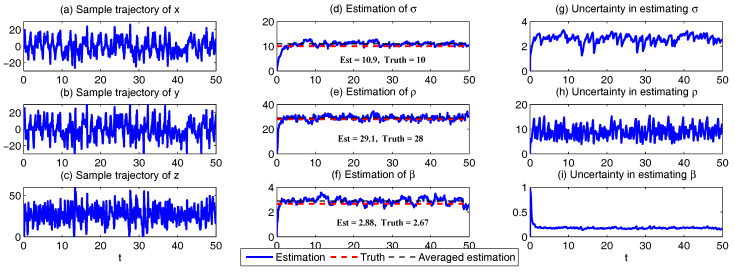
Parameter estimation of the noisy L-63 system using the method with stochastic parameterized Equations (146) and (147) with $\sigma_{x}=\sigma_{y}=\sigma_{z}=15$. (**a**–**c**) sample trajectories of x,y,z which are used as observations; (**d**–**f**) time evolution of the estimated parameters and the truth (red); the thin black line shows the average of the estimation from t=10 to t=50; (**g**)–(**i**) the uncertainty evolution of each estimated parameter.

Figure 30 shows the results of parameter estimation using the method with stochastic parameterized Equations (146) and (147). Despite the error in $\hat{\sigma },\hat{\rho }$ and $\hat{\beta }$ as the prior information, the estimation of the parameters is quite accurate. In fact, by using the averaged value over the time interval t∈[10,50] as the estimation, we compare the estimated parameters with the truth:

(150) $$\begin{array}{cc} \mathrm{Estimation}:\; & \sigma =10.9,\;\rho =29.1,\;\beta =2.88,\\ \mathrm{Truth}:\; & \sigma =10.0,\;\rho =28.0,\;\beta =2.67. \end{array}$$

The relative error in the three estimated parameters compared with the truth is 9.0%,3.9% and 7.8%, respectively, all of which are much less than 20% in $\hat{\sigma },\hat{\rho }$ and $\hat{\beta }$. This is because the conditional Gaussian framework (1) and (2) automatically makes use of the information in both the observations and the stochastic parameterized equations. The combination of these two components reduces the error even if the prior knowledge in the stochastic parameterized equations is biased (as in most of the real-world applications). Notably, the convergence of the estimated parameters as shown in Figure 30 is much faster than the direct method. This is one of the key features of the approach using stochastic parameterized equations and is practically quite useful since only limited observational data is known in many applications.

It is also important to note that the perfect model is unknown in most realistic situations. Therefore, dealing with model error in the parameter estimation is a crucial topic. The judicious model error in the approach using stochastic parameterized equations allows parameter uncertainties and provides more robust results compared with the direct method. Thus, the approach using stochastic parameterized equations has a wider application in real-world problems. More comparison between the two methods developed here can be found in [28].

#### 6.1.3. Estimating Parameters in the Unresolved Processes

As discussed in Section 4.5.1 and Section 6.1.2, stochastic parameterizations are widely used in describing turbulent signals as observed in nature. One important step is to estimate the parameters in the equations associated with these stochastic parameterizations (e.g, $\sigma_{\gamma },\hat{\gamma },d_{\gamma },\sigma_{\omega },\hat{\omega },d_{\omega },\sigma_{b},\hat{b}$ and $d_{b}$ in (119)). Note that there are typically no direct observations of these stochastic parameterized processes since they usually represent unresolved variables or they may not have clear physical correspondence. However, they play important roles in describing the underlying complex turbulent dynamical system. An accurate estimation of these parameters is crucial. See examples in Section 4.5.1 and Section 6.1.2 and those in [65,101].

Traditional methods in estimating these parameters include Markov Chain Monte Carlo (MCMC) [198,199,200,201], maximum likelihood estimation [202,203] and the ensemble Kalman filter [204,205]. Note that, if both the state variables γ,ω,b and the associated 9 parameters in these three equations are treated as unobserved variables $u_{\mathrm{II}}$, then the augmented system does not belong to the conditional Gaussian model family. Thus, the closed form in (4) and (5) cannot be applied in the two methods discussed in Section 6.1.1 and Section 6.1.2 and ensemble Kalman filter has to be adopted in these methods, which is computationally expensive.

Nevertheless, a judicious application of the conditional Gaussian framework still allows an efficient parameter estimation algorithm for the parameters in the stochastic parameterized equations. Below, we discuss the idea using the so-called SPEKF-M model, which is a simplified version of the SPEKF model (119) and contains only the multiplicative noise process γ (and “M” stands for multiplicative). The model reads

(151) $$\begin{array}{cc} du & =\left(-\gamma u+F(t)\right)dt+\sigma_{u}dW_{u},\\ d\gamma & =-d_{\gamma }\left(\gamma -\hat{\gamma }\right)dt+\sigma_{\gamma }dW_{\gamma }. \end{array}$$

The goal here is to estimate the three parameters $\sigma_{\gamma },d_{\gamma }$ and $\hat{\gamma }$ by observing only one sample trajectory of *u*. First, choose an arbitrary initial estimation of the three parameters $\sigma_{\gamma }^{\left(0\right)},d_{\gamma }^{\left(0\right)}$ and ${\hat{\gamma }}^{\left(0\right)}$. Then, run the conditional Gaussian filter (4) and (5), which gives a time series of the conditional mean ${\bar{\gamma }}^{\left(0\right)}\left(t\right)$ and another time series of the conditional variance $R^{\left(0\right)}\left(t\right)$ of γ(t) conditioned on the trajectory of *u*. With the ${\bar{\gamma }}^{\left(0\right)}\left(t\right)$ and $R^{\left(0\right)}\left(t\right)$, applying the same argument as that in (124) but changing the *L* Gaussian distributions associated with *L* different trajectories by those at *L* different time instants, the distribution $p(\gamma^{\left(0\right)})$ can be obtained via a Gaussian mixture. Here, $p(\gamma^{\left(0\right)})$ is also a Gaussian distribution due to its dynamical structure in (151). This Gaussian distribution provides two quantities—the mean $\mu_{\gamma ,eq}^{\left(0\right)}$ and the variance $R_{\gamma ,eq}^{\left(0\right)}$. In addition, the sample autocorrelation function $\tau_{\gamma ,eq}^{\left(0\right)}$ of the conditional mean ${\bar{\gamma }}^{\left(0\right)}\left(t\right)$ can be easily computed. Therefore, these three quantities are used to update the iteration of the three parameters $\sigma_{\gamma },d_{\gamma }$ and $\hat{\gamma }$ with the relation

(152) $$\frac{{\left(\sigma_{\gamma }^{\left(1\right)}\right)}^{2}}{2d_{\gamma }^{\left(1\right)}}=R_{\gamma }^{\left(0\right)},\;{\hat{\gamma }}^{\left(1\right)}=\mu_{\gamma ,eq}^{\left(0\right)},\;d_{\gamma }^{\left(1\right)}=\frac{1}{\tau_{\gamma ,eq}^{\left(0\right)}}.$$

Then, run the conditional Gaussian filter (4) and (5) again using the same observed time series u(t) but with the updated parameters $\sigma_{\gamma }^{\left(1\right)},d_{\gamma }^{\left(1\right)}$ and ${\hat{\gamma }}^{\left(1\right)}$. Repeating the above procedure leads to another update of the parameters $\sigma_{\gamma }^{\left(2\right)},d_{\gamma }^{\left(2\right)}$ and ${\hat{\gamma }}^{\left(2\right)}$. Continue this process until the updates of the parameters converge. This fixed point iteration results in an efficient parameter estimation algorithm.

As a simple test example, the following parameters are adopted to generate the true signal in the SPEKF-M model (151),

(153) $$\sigma_{\gamma }=0.5,\;d_{\gamma }=0.5,\;\hat{\gamma }=1,\;\sigma_{u}=0.5,\;F=2.$$

**Figure 31:**
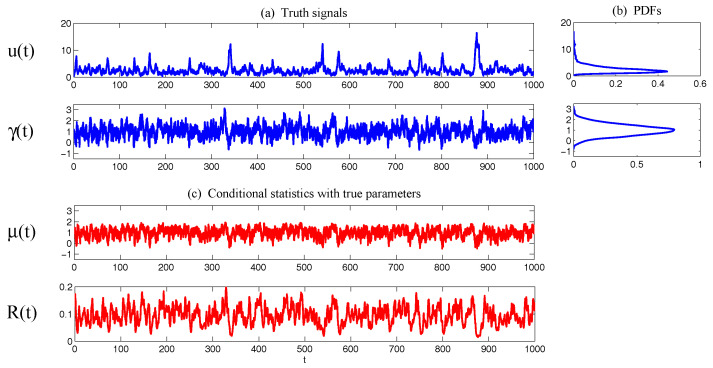
(**a**) a pair of sample trajectories (u(t),γ(t)) of the SPEKF-M model (151) with parameters in (153); (**b**) the corresponding PDFs. Note that the PDF of *u* is high non-Gaussian with an one-side fat tail; (**c**) the conditional mean μ(t) and conditional variance R(t) using the model with perfect parameters.

A pair of sample trajectories (u(t),γ(t)) is shown in Panel (a) of Figure 31 and the corresponding PDFs are shown in Panel (b). Clearly, these parameters result in a highly intermittent time series of u(t) with a non-Gaussian PDF with an one-side fat tail. Panel (c) shows the conditional mean and conditional variance of γ conditioned on the trajectory of *u* with the perfect parameters. The conditional mean does not equal the truth, but the conditional mean μ(t) at the intermittent phases of *u* are close to the truth of γ with a small conditional variance. On the other hand, at quiescent phases, μ(t) differs from the truth while the uncertainty (conditional variance) in the estimation is large.

**Figure 32:**
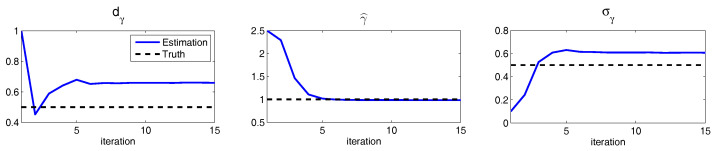
Iteration of the updates of the parameters (blue curve). The initial values $\sigma_{\gamma }^{\left(0\right)}=0.1,d_{\gamma }^{\left(0\right)}=1$ and ${\hat{\gamma }}^{\left(0\right)}=2.5$ are far from the truth (black dashed lines).

**Figure 33:**
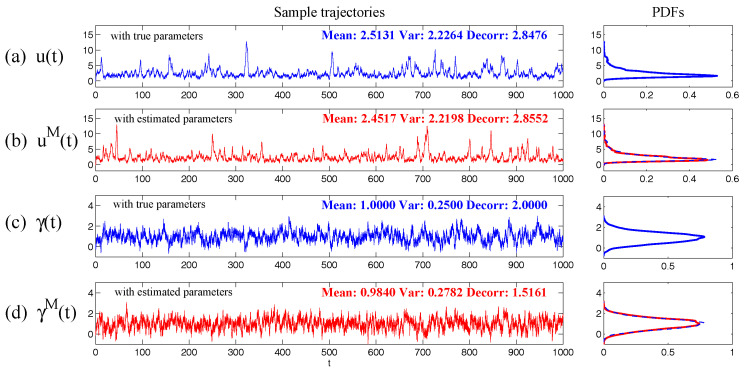
Sample trajectories and the associated PDFs using the true parameters (blue) and the estimated ones (red). (**a**) u(t); (**b**) $u^{M}\left(t\right)$; (**c**) γ(t); (**d**) $\gamma^{M}\left(t\right)$. The PDFs are calculated based on a long trajectory with 10,000 time units but only 1000 time units are shown here. In the panels of the PDFs of $u^{M}$ and $\gamma^{M}$, those of *u* and γ are also illustrated (thin dashed blue curves) for comparison.

Now, we apply the algorithm above to estimate the three parameters $\sigma_{\gamma },d_{\gamma }$ and $\hat{\gamma }$ assuming the other two parameters $\sigma_{u}=0.5$ and F=2 are known. The trajectory of *u* (with the length of 1000 time units) in Panel (a) of Figure 31 is used as the input of the algorithm. The iteration curve of the algorithm is shown in Figure 32, where the initial values $\sigma_{\gamma }^{\left(0\right)}=0.1,d_{\gamma }^{\left(0\right)}=1$ and ${\hat{\gamma }}^{\left(0\right)}=2.5$ are far from the truth. It is clear that, after five iterations, the update of the parameters converges to fixed points. The parameter $\hat{\gamma }$ converges exactly to the truth while both $d_{\gamma }$ and $\sigma_{\gamma }$ are slightly larger than the true values. Nevertheless, the variance of γ using the estimated parameters, namely $\sigma_{\gamma }^{2}/\left(2d_{\gamma }\right)=0.61^{2}/\left(2\cdot 0.66\right)=0.28$, is close to that of the truth, which is $0.5^{2}/\left(2\cdot 0.5\right)=0.25$. The slightly overestimation of the variance compensates the slightly underestimation of the damping time $1/d_{\gamma }$ and therefore the model with the estimated parameters is able to generate essentially the same intermittent signals as in the observed period of *u* in Figure 31. Note that since the input of the time series of *u* is only of finite length (and is actually pretty short here), the estimated parameters reflect the features in the observed signal, which may be slightly different from the true underlying dynamics unless an infinitely long time series is used as the input. Next, the skill of the estimated parameters is examined via the model statistics. In Figure 33, both the trajectories and the associated PDFs of *u* and γ using the true parameters and the estimated parameters are shown. Due to the high skill in estimating both the mean and variance of γ, the PDFs of $\gamma^{M}$ and γ are quite close to each other with only a slight overestimation of the variance in $\gamma^{M}$. The decorrelation time of the trajectory of $\gamma^{M}$ is slightly shorter than that of γ as discussed above. Nevertheless, $\gamma^{M}$ and γ provide quite similar feedback to $u^{M}$ and *u*. Therefore, the statistical features in $u^{M}$, including the decorrelation time, mean, variance and highly non-Gaussian PDF, are almost the same as those in *u*.

A more detailed study of this algorithm, including the application to the full SPEKF model (119) and the convergence rate dependence on different model ingredients, will be included in a follow-up work.

### 6.2. Data Assimilation with Physics-Constrained Forecast Models and Information Theory for Quantifying Model Errors

#### 6.2.1. An Information Theoretical Framework for Data Assimilation and Prediction

The following two traditional path-wise measurements are widely used in assessing the skill in data assimilation and prediction [15,206,207,208,209,210]. Denote $u_{i}$, i=1,…,n the true signal and ${\hat{u}}_{i}$ the filtering/prediction estimate. These measurements are given by

1. The root-mean-square error (RMSE):
  (154) $$RMSE=\sqrt{\frac{\sum_{i=1}^{n}{\left({\hat{u}}_{i}-u_{i}\right)}^{2}}{n}}.$$
2. The pattern correlation (PC):
  (155) $$PC=\frac{\sum_{i=1}^{n}\left({\hat{u}}_{i}-{\bar{\hat{u}}}_{i}\right)\left(u_{i}-{\bar{u}}_{i}\right)}{\sqrt{\sum_{i=1}^{n}{\left({\hat{u}}_{i}-{\bar{\hat{u}}}_{i}\right)}^{2}}\sqrt{\sum_{i=1}^{n}{\left(u_{i}-{\bar{u}}_{i}\right)}^{2}}},$$

where ${\bar{\hat{u}}}_{i}$ and ${\bar{u}}_{i}$ denotes the mean of ${\hat{u}}_{i}$ and $u_{i}$, respectively.

While these two path-wise measurements are easy to implement and are able to quantify the data assimilation and prediction skill to some extent, they have fundamental limitations. It has been shown in [42,211] that these two measurements fail to quantify the skill of capturing the extreme events and other non-Gaussian features, which lead to misleading results. In fact, concrete examples even in the Gaussian models [42,211] showed that two different predictions can have the same RMSE and PC, but one is way more skillful than the other in capturing the extreme events.

Due to the fundamental limitations of assessing the data assimilation and prediction skill based only on these two traditional path-wise measurements, various information measurements have been proposed to improve the quantification of the data assimilation and prediction [16,194,195,212,213,214,215,216,217,218]. In [218,219,220,221,222], an information measurement called Shannon entropy difference was introduced and was used to assess the data assimilation and prediction skill. The Shannon entropy difference takes into account the estimation of the extreme events and improves the insufficiency in the traditional path-wise measurements. However, relying solely on the Shannon entropy difference in assessing the data assimilation and prediction skill is also misleading. In fact, the Shannon entropy difference fails to capturing the model error in the mean state and it computes the uncertainty of the two distributions separately rather than considering the pointwise difference between the two PDFs.

Due to the fundamental limitations in the two classical path-wise measurement, RMSE and PC, as well as those in the Shannon entropy difference, a new information-theoretic framework [186] has developed to assess the data assimilation/prediction skill. Denote π≡π(u) and $\pi^{M}\equiv \pi \left(u^{M}\right)$ the PDFs associated with truth *u* and the data assimilation/prediction estimate $u^{M}$, respectively. Denote $p(u,u^{M})$ the joint PDF of *u* and $u^{M}$. Let $U=u-u^{M}$ be the residual between the truth and the estimate. This information-theoretic framework involves three information measurements:

1. The Shannon entropy residual,
  (156) S(U)=−∫p(U)logp(U).
2. The mutual information,
  (157) $$M\left(\pi ,\pi^{M}\right)=\;\int \int \;p\left(u,u^{M}\right)\log\left(\frac{p(u,u^{M})}{\pi \left(u\right)\pi \left(u^{M}\right)}\right).$$
3. The relative entropy,
  (158) $$R\left(\pi ,\pi^{M}\right)=-\int \pi \log\left(\frac{\pi }{\pi^{M}}\right).$$

The Shannon entropy residual quantifies the uncertainty in the point-wise difference between *u* and $u^{M}$. It is an information surrogate of the RMSE in the Gaussian framework. The mutual information quantifies the dependence between the two processes. It measures the lack of information in the factorized density $\pi \left(u\right)\pi \left(u^{M}\right)$ relative to the joint density $p(u,u^{M})$, which follows the identity,

(159) $$M\left(\pi ,\pi^{M}\right)=P\left(p\left(u,u^{M}\right),\pi \left(u\right)\pi \left(u^{M}\right)\right).$$

The mutual information is an information surrogate of the PC in the Gaussian framework. On the other hand, the relative entropy quantifies the lack of information in $\pi^{M}$ related to π and it is a good indicator of the skill of $u^{M}$ in capturing the peaks and extreme events of *u*. It also takes into account the pointwise discrepancy between π and $\pi^{M}$ rather than only computing the difference between the uncertainties associated with the two individual PDFs (as in the Shannon entropy difference). Therefore, the combination of these three information measurements is able to capture all the features in assessing the data assimilation/prediction skill and overcomes the shortcomings as discussed in the previous subsection. Note that, when $\pi ∼N(\bar{u},R)$ and $\pi^{M}∼N\left({\bar{u}}^{M},R_{M}\right)$ are both Gaussian, then the above three information measurements have explicit expressions [215].

The information-theoretic framework (156)–(158) is usually defined in the super-ensemble sense [215]. Note that in many practical issues only one realization (trajectory) is available. Nevertheless, the information-theoretic framework can also be used in a pathwise way, where the statistics are computed by collecting all the sample points in the given realization. Some realistic applications of the information-theoretic framework for filtering and prediction can be found in [42,63,215].

#### 6.2.2. Important Roles of Physics-Constrained Forecast Models in Data Assimilation

It has been shown in Section 3.3 that the physics-constrained nonlinear stochastic models are the recent development for data-driven statistical models with partial observations. The physics-constrained nonlinear stochastic models overcome the finite-time blowup and the lack of physical meaning issues in various ad hoc multi-layer regression models [31,32]. Here, our goal is to show that the physics-constrained nonlinear stochastic models also play important role in data assimilation (or filtering). Ignoring the energy-conserving nonlinear interactions in the forecast models will result in large model errors.

The test model below is a simple dyad model, which mimics the interactions between resolved large-scale mean flow and unresolved turbulent fluctuations with intermittent instability [37,50,223]. The dyad model reads

(160) $$\begin{array}{cc} du & =\left(-d_{u}u+\gamma uv+F_{u}\right)dt+\sigma_{u}dW_{u}, \end{array}$$

(161) $$\begin{array}{cc} dv & =\left(-d_{v}v-\gamma u^{2}\right)dt+\sigma_{v}dW_{v}. \end{array}$$

In (160) and (161), *u* is regarded as representing one of the resolved modes in a turbulent signal, which interacts with the unresolved mode *v* through quadratic nonlinearities. The conserved energy in the quadratic nonlinear terms in (160) and (161) is easily seen. Below, the physics-constrained dyad model (160) and (161) is utilized to generate true signals of nature. The goal here is to filter the unobserved process *v* given one single realization of the observed process *u*. In addition to adopting the perfect filter (160) and (161), an imperfect filter with no energy-conserving nonlinear interactions is studied for comparison. In this imperfect filter, the nonlinear feedback $-\gamma u^{2}$ in *v* is dropped and the result is a stochastic parameterized filter [20],

(162) $$\begin{array}{cc} du & =\left(-d_{u}u+\gamma uv+F_{u}\right)dt+\sigma_{u}dW_{u}, \end{array}$$

(163) $$\begin{array}{cc} dv & =-d_{v}^{M}\left(v-{\bar{v}}^{M}\right)dt+\sigma_{v}^{M}dW_{v}. \end{array}$$

In the stochastic parameterized filter (162) and (163), the parameters in the resolved variable *u* are assumed to be the same as nature (160) and (161). We further assume the statistics of the unobserved variable *v* of nature (160) and (161) are available. Thus, the parameters $d_{v}^{M},{\bar{v}}^{M}$ and $\sigma_{v}^{M}$ in the unresolved process *v* are calibrated [187,188,224] by matching the mean, variance and decorrelation time of those in (160) and (161). Note that both (160) and (161) and (162) and (163) belong to the conditional Gaussian framework (1) and (2) by denoting $u_{I}=u$ and $u_{\mathrm{II}}=v$ and (4) and (5) is used to efficiently calculate the filter estimates.

Note that in (160) and (161), if $F_{u}=0$, then the fixed point associated with the deterministic part is $u_{c}$ = $v_{c}$ = 0. This leads to an important issue in the state estimation of *v*, namely the observability [207,225]. The coupled system (160) and (161) is said to lose its observability if the observed process *u* provides no information in determining the unobserved variable *v*. Intuitively, this corresponds to u=0 in (160) and (161), in which case *v* disappears in the observed process *u*. Therefore, if $F_{u}=0$, then the filtering skill of *v* is expected to deteriorate especially with a small $\sigma_{u}$. Below, we consider two different dynamical regimes:

(164) $$\begin{array}{cc} \mathrm{Regime}\;I:=\; & d_{u}=0.8,\;d_{v}=0.8,\;\gamma =1.2,\;and\;F_{u}=1,\\ \mathrm{Regime}\;\mathrm{II}:\; & d_{u}=0.8,\;d_{v}=0.8,\;\gamma =1.2,\;and\;F_{u}=0. \end{array}$$

In Regime II, there is no practical observability in the quiescent phase (near the fixed point associated with the deterministic model) while in Regime I the forcing drives the signal out of the value with $u_{c}=0$.

**Figure 34:**
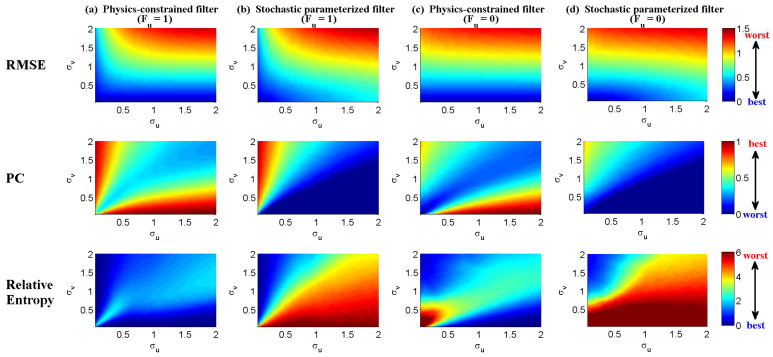
Model error in terms of RMSE, PC and relative entropy as a function of $\sigma_{u}$ and $\sigma_{v}$ in the true signal. (**a**,**b**) Regime I with $F_{u}=1$; (**c**,**d**) Regime I with $F_{u}=0$; (**a**,**c**) show the model error using the perfect physics-constrained forecast model (160) and (161) while (**b**,**d**) uses the stochastic parameterized filter (162) and (163).

Figure 34 shows the model error in terms of RMSE, PC and relative entropy as a function of $\sigma_{u}$ and $\sigma_{v}$. Both the perfect physics-constrained forecast model (160) and (161) and the stochastic parameterized filter (162) and (163) are used. Here, instead of showing the Shannon entropy residual and the mutual information, we still use the RMSE and PC since most readers are familiar with these two measurements. Nevertheless, the readers should keep in mind that the Shannon entropy residual and the mutual information are more suitable measurements in the non-Gaussian framework. On the other hand, the relative entropy is shown here in assessing the model error.

**Figure 35:**
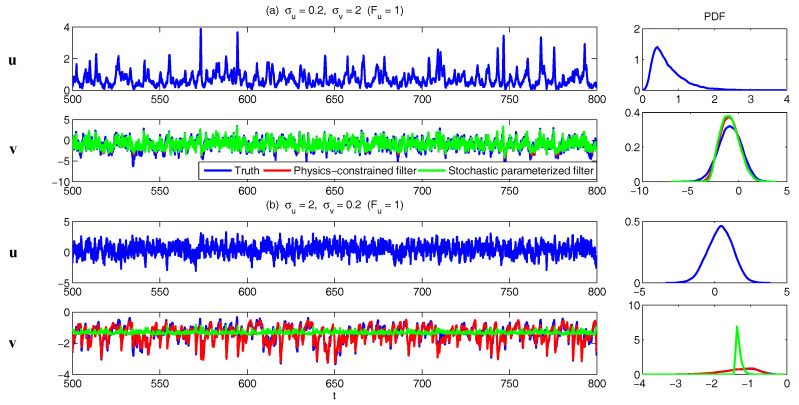
Regime I with $F_{u}=1$. Path-wise trajectories and the PDFs of the true signal (blue) and the filter estimation of the physics-constrained forecast model (160) and (161) (red) and the stochastic parameterized filter (162) and (163) (green). (**a**) $\sigma_{u}=0.2$ and $\sigma_{v}=2$; (**b**) $\sigma_{u}=2$ and $\sigma_{v}=0.2$.

First, in Columns (a,b) of Figure 34, the filtering skill in Regime I with $F_{u}=1$ is illustrated. With a small $\sigma_{u}$, both filters have skillful estimation. This is because when $\sigma_{u}$ is small, the filters trust more towards the observational process, which has a large signal to noise ratio and therefore it provides accurate estimates. However, when $\sigma_{u}$ is large but $\sigma_{v}$ is small in generating the true signal, the stochastic parameterized filter becomes much worse than the perfect filter using physics-constrained forecast model. In fact, a large $\sigma_{u}$ leads to large signals in *u* and it also tells the filter to trust more towards the underlying process of *v*. This implies the filter estimate of *v* is then essentially driven by the feedback term $-\gamma u^{2}$. Since the stochastic parameterized filter has no such feedback mechanism, the error becomes large. See Panel (b) of Figure 35 for an example with path-wise trajectories. It is also important to note that in such a situation the PDF of the filter estimate is completely different from the truth and thus a large model error is found.

**Figure 36:**
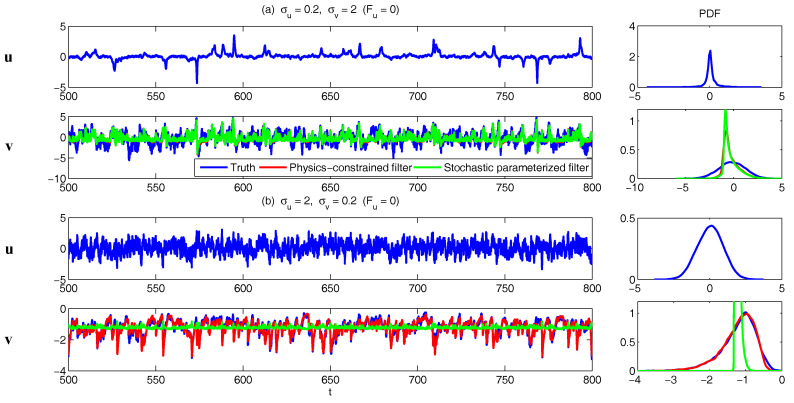
Regime II with $F_{u}=0$. Path-wise trajectories and the PDFs of the true signal (blue) and the filter estimation of the physics-constrained forecast model (160) and (161) (red) and the stochastic parameterized filter (162) ans (163) (green). (**a**) $\sigma_{u}=0.2$ and $\sigma_{v}=2$; (**b**) $\sigma_{u}=2$ and $\sigma_{v}=0.2$.

Columns (c,d) of Figure 34 show the filter estimates in Regime II with $F_{u}=0$. Compared with the results in Regime I, it is clear that when $\sigma_{u}$ is small in generating the true signal, the filter estimates become inaccurate. This is in fact due to the observability issue since small $\sigma_{u}$ means the signal of *u* stays near original. This is clearly seen in Panel (a) of Figure 36, where the filter estimate is accurate only at intermittent phases. One interesting phenomenon is that although the filter estimate using the stochastic parameterized filter in Panel (b) of Figure 35 has a smaller RMSE compared with that in Panel (a) of Figure 36, the relative entropy clearly indicates a much worse filter estimates in the former case since they fail to capture any of the amplitudes. These facts all indicate the importance in including the physics-constrained structure in designing filters especially in the regimes that are dominated by the energy-conserving nonlinear interactions.

Finally, it is important to note that although the stochastic parameterized filter (162) and (163) is not able to recover the signal due to the strong feedback from the physics-constrained nonlinear interactions, the stochastic parameterized filter (162) and (163) is still quite useful in detecting the intermittent phases in turbulent signals. In fact, in Panel (a) of both Figure 35 and Figure 36, the intermittent phases are all accurately recovered by the stochastic parameterized filter. Other works showing the skillful behavior of the stochastic parameterized filter and its advantages over the mean stochastic models can be found in [16,64,65].

### 6.3. Multiscale Data Assimilation with Particles Interacting with Conditional Gaussian Statistics

#### 6.3.1. A General Description

Data assimilation of turbulent signals is an important challenging problem because of the extremely complicated large dimension of the signals and incomplete partial noisy observations which usually mix the large scale mean flow and small scale fluctuations. See Chapter 7 of [20] for examples of new phenomena due to this multiscale coupling through the observations even for linear systems. Due to the limited computing power, it is desirable to use multi-scale forecast models which are cheap and fast to mitigate the curse of dimensionality in turbulent systems. Thus, model errors from imperfect forecast models are unavoidable in the development of a data assimilation method in turbulence.

Among different methods, conventional superparameterization is a multi-scale algorithm that was originally developed for the purpose of parameterizing unresolved cloud process in tropical atmospheric convection [73,226,227]. This conventional superparameterization resolves the large scale mean flow on a coarse grid in a physical domain while the fluctuating parts are resolved using a fine grid high resolution simulation on periodic domains embedded in the coarse grid. A much cheaper version of superparameterization, called stochastic superparameterization [70,71,72,73], replaces the nonlinear eddy terms by quasilinear stochastic processes on formally infinite embedded domains where the stochastic processes are Gaussian conditional to the large scale mean flow. The key ingredient of these multiscale data assimilation methods is the systematic use of conditional Gaussian mixtures which make the methods efficient by filtering a subspace whose dimension is smaller than the full state. This conditional Gaussian closure approximation results in a seamless algorithm without using the high resolution space grid for the small scales and is much cheaper than the conventional superparameterization, with significant success in difficult test problems [71,72,74] including the MMT model [71,75] and ocean turbulence [76,77,78].

The key idea of the multiscale data assimilation method is to use conditional Gaussian mixtures [80,228] whose distributions are compatible with superparameterization. The method uses particle filters (see [79] and Chapter 15 of [20]) or ensemble filters on the large scale part [75,76] whose dimension is small enough so that the non-Gaussian statistics of the large scale part can be calculated from a particle filter, whereas the statistics of the small scale part are conditionally Gaussian given the large scale part. This framework is not restricted to superparameterization as the forecast model and other cheap forecast models can also be employed. See [80] for another multiscale filter with quasilinear Gaussian dynamically orthogonality method as the forecast method in an adaptively evolving low dimensional subspace without using superparameterization. Note that data assimilation using superparameterization has already been discussed in [229] with noisy observations of the large scale part of the signal alone. There it was shown that, even in this restricted setting, ignoring the small scale fluctuations even when they are rapidly decaying can completely degrade the filter performance compared with the high skill using superparameterization. Here, in contrast to [229], we consider multiscale data assimilation methods with noisy observations with contributions from both the large and small scale parts of the signal, which is a more difficult problem than observing only the large scale because it requires accurate estimation of statistical information of the small scales [75,76,230]. In addition, mixed observations of the large and small scale parts occur typically in real applications. For example, in geophysical fluid applications, the observed quantities such as temperature, moisture, and the velocity field necessarily mix both the large and small scale parts of the signal [20,231]. Thus, the multiscale filtering also provides a mathematical framework for representation errors, which are due to the contribution of unresolved scales [81,82] in the observations.

#### 6.3.2. Particle Filters with Superparameterization

Superparameterization retains the large scale variables by resolving them on a coarse grid while the effect of the small scales on the large scales is parameterized by approximating the small scales on local or reduced spaces. Stochastic superparameterization discussed in the previous section uses Gaussian closure for the small scales conditional to the large scale variable $\bar{u}$ with $\bar{u}\in R^{N}$ [70,71,72,73]. Thus, we consider a multi-scale filtering algorithm with forecast prior distributions given by the conditional distribution

(165) $$p^{f}\left(u\right)=p^{f}\left(\bar{u},u^{\prime }\right)=p^{f}\left(\bar{u}\right)p_{G}^{f}\left(u^{\prime }|\bar{u}\right),$$

where $p_{G}^{f}\left(u^{\prime }|\bar{u}\right)$ is a Gaussian distribution conditional to $\bar{u}$

(166) $$p_{G}^{f}\left(u^{\prime }|\bar{u}\right)=N\left(u^{\prime }\left(\bar{u}\right),R^{\prime }\left(\bar{u}\right)\right).$$

Here, we assume that $N_{1}$ is sufficiently small enough that particle filters (see Chapter 15 of [20]) can be applied to the large scales. For a low dimensional space $\bar{u}$, the marginal distribution of $\bar{u}$ can be approximated by Q particles

(167) $$p^{f}\left(\bar{u}\right)=\sum_{j=1}^{Q}p_{j}^{f}\delta \left(\bar{u}-{\bar{u}}_{j}\right),$$

where $p_{j}^{f}\geq 0$ are particle weights such that $\sum_{j}p_{j}^{f}=1$. After the forecast step where superparameterization is applied to each particle member, we have the following general form for the prior distribution $p^{f}\left(u\right)$

(168) $$\begin{array}{cc} p^{f}\left(u\right)=p^{f}\left(\bar{u},u^{\prime }\right) & =\sum_{j=1}^{Q}p_{j}^{f}\delta \left(\bar{u}-{\bar{u}}_{j}\right)p_{G}^{f}\left(u^{\prime }f|{\bar{u}}_{j}\right)\\ & =\sum_{j=1}^{Q}p_{j}^{f}\delta \left(\bar{u}-{\bar{u}}_{j}\right)N\left(u^{\prime }{\left({\bar{u}}_{j}\right)}^{f},R^{\prime }{\left({\bar{u}}_{j}\right)}^{f}\right), \end{array}$$

which is a conditional Gaussian mixture distribution where each summand is a Gaussian distribution conditional to ${\bar{u}}_{j}$. The Gaussian mixture has already been used in data assimilation [232,233,234] but the multi-scale method developed here is different in that conditional Gaussian distributions are applied in the reduced subspace $u^{\prime }$ with particle approximations only in the lower dimensional subspace $\bar{u}$. Thus, the proposed multi-scale data assimilation method can be highly efficient and fast in comparison with conventional data assimilation methods which use the whole space for the filter.

Assume that the prior distribution from the forecast is in the from (168) and that the observations have the following structure:

(169) $$v=G\left(\bar{u},u^{\prime }\right)+\sigma_{\theta }=\bar{G}\bar{u}+G^{\prime }\left(\bar{u}\right)u^{\prime }+\sigma_{\theta },$$

where $G^{\prime }\left({\bar{u}}_{j}\right)$ has rank *M* and $\sigma_{\theta }$ is the observation noise error which is Gaussian. Then, the posterior distribution in the analysis step taking into account the observations (169) is in the form of (168)

(170) $$p^{a}\left(u\right)=p^{a}\left(\bar{u},u^{\prime }\right)=\sum_{j=1}^{Q}p_{j}^{a}\delta \left(\bar{u}-{\bar{u}}_{j}\right)N\left(u^{\prime }{\left({\bar{u}}_{j}\right)}^{a},R^{\prime }{\left({\bar{u}}_{j}\right)}^{a}\right).$$

The new mixture weights are

(171) $$p_{j}^{a}=\frac{p_{j}^{f}I_{j}}{\sum_{k=1}^{Q}p_{k}^{f}I_{k}},$$

where $I_{j}=\int p\left(v|{\bar{u}}_{j},u^{\prime }\right)p\left(u^{\prime }|{\bar{u}}_{j}\right)du^{\prime }$ and for each particle ${\bar{u}}_{j}$, the posterior mean and variance of $u^{\prime }$, $u^{\prime }{\left({\bar{u}}_{j}\right)}^{a}$ and $R^{\prime }{\left({\bar{u}}_{j}\right)}^{a}$, respectively, are

(172) $$\begin{array}{cc} {u^{\prime }\left({\bar{u}}_{j}\right)}^{a} & ={u^{\prime }}^{f}+K^{\prime }\left(v-\bar{G}{\bar{u}}_{j}^{f}-G^{\prime }\left({\bar{u}}_{j}^{f}\right)u^{\prime }\right),\\ R^{\prime }{\left({\bar{u}}_{j}\right)}^{a} & =\left(I-K^{\prime }G^{\prime }\left({\bar{u}}_{j}^{f}\right)\right)R^{\prime }{\left(\bar{u}\right)}^{f}, \end{array}$$

where the Kalman gain matrix $K^{\prime }$ is given by

(173) $$K^{\prime }=R^{\prime f}G^{\prime }{\left({\bar{u}}_{j}^{f}\right)}^{T}{\left(G^{\prime }\left({\bar{u}}_{j}^{f}\right)R^{\prime f}G^{\prime }{\left({\bar{u}}_{j}^{f}\right)}^{T}+r_{\theta }\right)}^{-1}.$$

See the supplementary material of [80] for more details.

#### 6.3.3. Clustered Particle Filters and Mutiscale Data Assimilation

Clustered particle filters (CPFs) [40,235] are a new class of particle filters, introduced for high-dimensional dynamical systems such as geophysical systems. The clustered particle filters use relatively few particles compared with the standard particle filter and capture the non-Gaussian features of the true signal, which are typical in complex nonlinear systems. The method is also robust for the difficult regime of high-quality sparse and infrequent observations and does not show any filter divergence in our tests. In the clustered particle filter, coarse-grained localization is implemented through the clustering of state variables and particles are adjusted to stabilize the filter.

One of the key features of the CPF is particle adjustment. Particle adjustment updates the prior particles closer to the observation instead of reweighing the particles when the prior is too far from the observation likelihood. When observations are sparse, unobserved adjacent state variables must have the same particle weights with the observed variable as they are updated using cross-correlations. For this purpose, the CPF partitions the state variables into nonoverlapping clusters $\left\{C_{l},l=1,2,\ldots ,N_{obs}\right\}$, where each cluster boundary is chosen as the midpoint of two adjacent observations, which is easily applicable to irregularly spaced observations. This yields $N_{obs}$ clusters corresponding to $N_{obs}$ observationlocations. Instead of using different weights for each state variable in the localized particle filter, the CPF uses scalar particle weights $\left\{\omega_{l,k}\right\}$ for the state variables in the same cluster $C_{l}$. For the substate vector $x_{C_{l}}=\left\{x_{i}|x_{i}\in C_{l}\right\}$ corresponding to cluster $C_{l}$, the CPF considers the marginalized PDF,

$$p\left(x_{C_{l}}\right)=\sum_{k}^{K}\omega_{l,k}\delta \left(x_{C_{l}}-x_{C_{l},k}\right)$$

and each observation $y_{j}$ updates only the marginalized PDF of the corresponding cluster that implements coarse-grained localization. Thus, the assimilation of the full state vector is decomposed into $N_{obs}$ independent assimilation problems for each cluster of a dimension smaller than the full state dimension $N_{state}$. Note that, in contrast to the localization using a smoothly varying correlation function with a localization radius parameter, the CPF has no adjustable parameter to tune localization. See [235] for more details.

The CPF can also be applied for multiscale particle filtering [40,235]. As a test example, it is shown below the skill of the multiscale cluster particle filter for the wave turbulence model introduced by Majda, McLaughlin and Tabak (MMT) [236,237] as a computationally tractable model of waveturbulence. The model is described by the following one dimensional partial differential equation for a complex scalar ψ:

(174) $$i\partial_{t}\psi ={\left|\partial_{x}\right|}^{1/2}\psi -{\left|\psi \right|}^{2}\psi +iF+iD\psi .$$

in a periodic domain of length *L* with large-scale forcing set to F=0.0163 sin(4πx/L) and dissipation *D* for both the large and small scales. It has several features of wave turbulence that make it a difficult test problem for data assimilation. The model has a shallow energy spectrum proportional to $k^{-5/6}$ for wavenumber *k* and an inverse cascade of energy from small to large scales. It also has non-Gaussian extreme event statistics caused by intermittent instability and breaking of solitons. Because the unresolved small scales carry more than two-thirds of the total variance, it is a difficult filtering problem to estimate resolved large scales using mixed observations of the large- and small-scale components.

Here, we compare the filtering results of the ensemble-based multiscale data assimilation method [75] and the multiscale CPF for the MMT model. As the forecast model for both filtering methods, we use the stochastic superparameterization multiscale method [73] as discussed in the previous subsection. The forecast model uses only 128 grid points, whereas the full resolution uses 8192 grid points, which yields about 250 times cheaper computational savings. Because the forecast model has a low computational cost compared with the full-resolution model, the forecast model has significant model errors. Observations of the full-scale variables are available at uniformly distributed 64 grid points (which are extremely sparse compared with the full-resolution 8192 grid points) with an observation error variance corresponding to 3% of the total climatological variance at every time interval of 0.25. The ensemble-based method uses the tuned parameters in [75] (i.e., a short localization radius 1 and 2% covariance inflation). For the hard threshold version CPF, the particle adjustment is triggered if either real or imaginary parts are not in the convex hull of the corresponding predicted observations as we observe both parts of the true signal. Both particle and ensemble-based methods use 129 samples.

**Figure 37:**
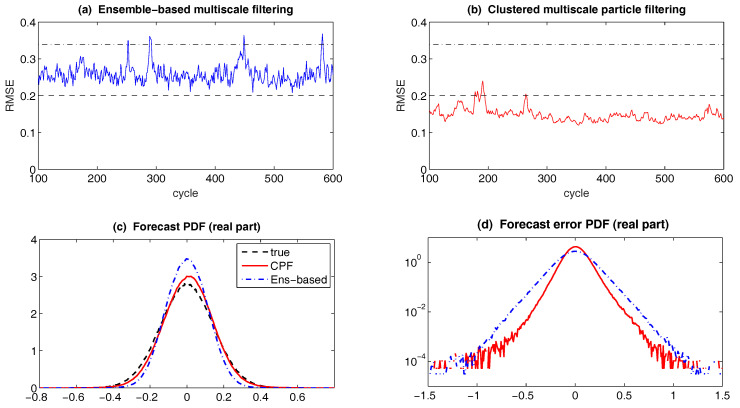
(**a**,**b**) time series of the large-scale estimation RMSE of the ensemble-based multiscale data assimilation method [75] and the CPF with 64 observations for the MMT model. The dashed line is the climatological error 0.20. The dashed and dotted line is the effective observation error 0.34; (**c**,**d**) large-scale forecast PDF and forecast error PDF using 64 observations.

The time series of the large-scale estimation RMSE of the ensemble-based filter and clustered multiscale particle filter are shown in Figure 37. The dashed and dotted line is the effective observation error 0.34, which is defined as $\sqrt{\mathrm{observation}\;\mathrm{error}\;\mathrm{variance}+\mathrm{small-scale}\;\mathrm{variance}}$ by treating the small-scale contribution as an additional error (i.e., a representation error). The dashed line is the climatological error 0.20, which is the standard deviation of the large-scale variables. The ensemble-based method has RMSE smaller than the effective observation error but larger than the climatological error. The CPF, however, shows skillful filter performance with RMSE smaller than the climatological error. The forecast PDFs and forecast error PDFs that show the prediction skill of the method are shown in Figure 37. The CPF has a better forecast PDF fit to the true signal and a narrower peak in the forecast error PDF than the ensemble-based method.

#### 6.3.4. Blended Particle Methods with Adaptive Subspaces for Filtering Turbulent Dynamical Systems

In the multi-scale data assimilation algorithms discussed above based on superparameterization, the subspace of particles defined by $\bar{u}$ is fixed. An attractive idea is to change the subspace with particles adaptively in time to capture the non-Gaussian features as they change in time. Very accurate filtering algorithms based on these ideas for multi-scale filtering utilizing this adaptive strategy have been developed [80,238]. Nonlinear statistical forecast models such as the modified quasilinear Gaussian [17,239] are implemented in the adaptive algorithm. In particular, the paper [238] also contains many detailed numerical experiments and interesting counterexamples to more naive strategies for multi-scale data assimilation.

#### 6.3.5. Extremely Efficient Multi-Scale Filtering Algorithms: SPEKF and Dynamic Stochastic Superresolution (DSS)

The SPEKF models as discussed in Section 4.5.1 are a class of nonlinear filters which are exact statistical equations for the mean and covariance for nonlinear forecast models that learn hidden parameters “on the fly” from the observed data. The parameters represent adaptive additive and multiplicative bias corrections from model error. They explicitly make judicious model error and utilize conditional Gaussian structure as developed in Section 4.5.1 above. The book [20] contains many examples and successful applications of this method.

Dynamical Stochastic Superresolution (DSS) uses the same idea but in addition exploits the aliased information in the observations to super-resolve a multi-scale turbulent signal [103,240]. Nontrivial applications of DSS including recovering geophysical turbulence from surface satellite observations [240] and filtering “black swans” and dispersive wave turbulence [103] with severe judicious model errors. An interesting mathematical problem is to understand the reasons for the skill of these radical methods. Recent progress in conceptual understanding of these methods for the two dimensional Navier–Stokes equations can be found in [104].

## 7. Conclusions

Multiscale nonlinear dynamical systems are ubiquitous in different areas, including geoscience, engineering, neural science and material science. In this article, a conditional Gaussian framework is developed and applied to the prediction, the state estimation and the uncertainty quantification of multiscale nonlinear stochastic systems. Despite the conditional Gaussianity, such systems are nevertheless highly nonlinear and are able to capture the non-Gaussian features of nature. The special structure of the system allows closed analytical formulae for solving the conditional statistics and is thus computationally efficient.

In Section 2, an overview of data, model and data-driven modeling framework is presented. Data and models are combined with each other to improve the understanding of nature and promote the filtering and prediction skill. However, solving the high-dimensional complex multiscale nonlinear dynamical systems in a direct way is computationally unaffordable and sometimes even not necessary. Therefore, cheap and effective approaches are required to efficiently solve the systems. Hybrid strategies can be used to greatly reduce the computational cost while they are able to preserve the key feature of the complex systems. In the hybrid strategies, particle methods are combined with analytically solvable conditional Gaussian statistics to deal with highly non-Gaussian characteristics in a relatively low dimensional subspace and the conditional Gaussian features in the remaining subspace, respectively. This indicates the importance of a systematic study of the conditional Gaussian system. Section 3 summarizes the general mathematical structure of nonlinear conditional Gaussian systems, the physics-constrained nonlinear stochastic models and the application of the MTV strategy to the conditional Gaussian systems. To show the wide application of the conditional Gaussian framework, a rich gallery of examples of conditional Gaussian systems are illustrated in Section 4, which includes data-driven physics-constrained nonlinear stochastic models, stochastically coupled reaction–diffusion models in neuroscience and ecology, large-scale dynamical models in turbulence, fluids and geophysical flow, and other models for filtering and predicting complex multiscale turbulent dynamical systems. Section 5 involves the effective statistically accurate algorithms that beat the curse of dimension for Fokker–Planck equation for conditional Gaussian systems. A hybrid strategy is developed where a conditional Gaussian mixture in a high-dimensional subspace via an extremely efficient parametric method is combined with a judicious non-parametric Gaussian kernel density estimation in the remaining low-dimensional subspace. For even larger dimensional systems, a judicious block decomposition and statistical symmetry are further applied that facilitate an extremely efficient parallel computation and a significant reduction of sample numbers. These algorithms are applied to the statistical prediction of a stochastically coupled FHN model with 1500 dimensions and an inhomogeneous two-layer Lorenz 96 model with 240 dimensions. Significant prediction skill shows the advantages of these algorithms in terms of both accuracy and efficiency. In Section 6, the conditional Gaussian framework is applied to develop extremely cheap multiscale data assimilation schemes, such as the stochastic superparameterization, which use particle filters to capture the non-Gaussian statistics on the large-scale part whose dimension is small whereas the statistics of the small-scale part are conditional Gaussian given the large-scale part. Other topics of the conditional Gaussian systems studied here include designing new parameter estimation schemes and understanding model errors using information theory.

The conditional Gaussian framework can also be used to study many other important topics. For example, the closed analytic formulae in the conditional statistics provide an efficient way to understand the causality between different processes in light of the information theory. The representation error is another important issue that requires a comprehensive study and the conditional Gaussian framework have great potentials to provide both theoretic and applied insights. In addition, model selection, model reduction and more studies on the parameter estimation are all important future works within the conditional Gaussian framework.
